# Benchmarking large language models’ performances for myopia care: a comparative analysis of ChatGPT-3.5, ChatGPT-4.0, and Google Bard

**DOI:** 10.1016/j.ebiom.2023.104770

**Published:** 2023-08-23

**Authors:** Zhi Wei Lim, Krithi Pushpanathan, Samantha Min Er Yew, Yien Lai, Chen-Hsin Sun, Janice Sing Harn Lam, David Ziyou Chen, Jocelyn Hui Lin Goh, Marcus Chun Jin Tan, Bin Sheng, Ching-Yu Cheng, Victor Teck Chang Koh, Yih-Chung Tham

**Affiliations:** a Yong Loo Lin School of Medicine, National University of Singapore, Singapore; b Centre of Innovation and Precision Eye Health, Department of Ophthalmology, Yong Loo Lin School of Medicine, National University of Singapore and National University Health System, Singapore; c Department of Ophthalmology, National University Hospital, Singapore; d Singapore Eye Research Institute, Singapore National Eye Centre, Singapore; e Department of Computer Science and Engineering, Shanghai Jiao Tong University, Shanghai, China; f Department of Endocrinology and Metabolism, Shanghai Jiao Tong University Affiliated Sixth People's Hospital, Shanghai Diabetes Institute, Shanghai Clinical Center for Diabetes, Shanghai, China; g MoE Key Lab of Artificial Intelligence, Artificial Intelligence Institute, Shanghai Jiao Tong University, Shanghai, China; h Eye Academic Clinical Program (Eye ACP), Duke NUS Medical School, Singapore

**Keywords:** ChatGPT-4.0, ChatGPT-3.5, Google Bard, Chatbot, Myopia, Large language models

## Abstract

**Background:**

Large language models (LLMs) are garnering wide interest due to their human-like and contextually relevant responses. However, LLMs’ accuracy across specific medical domains has yet been thoroughly evaluated. Myopia is a frequent topic which patients and parents commonly seek information online. Our study evaluated the performance of three LLMs namely ChatGPT-3.5, ChatGPT-4.0, and Google Bard, in delivering accurate responses to common myopia-related queries.

**Methods:**

We curated thirty-one commonly asked myopia care-related questions, which were categorised into six domains—pathogenesis, risk factors, clinical presentation, diagnosis, treatment and prevention, and prognosis. Each question was posed to the LLMs, and their responses were independently graded by three consultant-level paediatric ophthalmologists on a three-point accuracy scale (poor, borderline, good). A majority consensus approach was used to determine the final rating for each response. ‘Good’ rated responses were further evaluated for comprehensiveness on a five-point scale. Conversely, ‘poor’ rated responses were further prompted for self-correction and then re-evaluated for accuracy.

**Findings:**

ChatGPT-4.0 demonstrated superior accuracy, with 80.6% of responses rated as ‘good’, compared to 61.3% in ChatGPT-3.5 and 54.8% in Google Bard (Pearson's chi-squared test, all p ≤ 0.009). All three LLM-Chatbots showed high mean comprehensiveness scores (Google Bard: 4.35; ChatGPT-4.0: 4.23; ChatGPT-3.5: 4.11, out of a maximum score of 5). All LLM-Chatbots also demonstrated substantial self-correction capabilities: 66.7% (2 in 3) of ChatGPT-4.0's, 40% (2 in 5) of ChatGPT-3.5's, and 60% (3 in 5) of Google Bard's responses improved after self-correction. The LLM-Chatbots performed consistently across domains, except for ‘treatment and prevention’. However, ChatGPT-4.0 still performed superiorly in this domain, receiving 70% ‘good’ ratings, compared to 40% in ChatGPT-3.5 and 45% in Google Bard (Pearson's chi-squared test, all p ≤ 0.001).

**Interpretation:**

Our findings underscore the potential of LLMs, particularly ChatGPT-4.0, for delivering accurate and comprehensive responses to myopia-related queries. Continuous strategies and evaluations to improve LLMs’ accuracy remain crucial.

**Funding:**

Dr Yih-Chung Tham was supported by the 10.13039/501100001349National Medical Research Council of Singapore (NMRC/MOH/HCSAINV21nov-0001).

**Research in context**

**Evidence before this study**

We searched PubMed for articles on the performance evaluation of large language models (LLMs) in answering queries regarding myopia care, with no restrictions on the publication period or language. This was done by combining the search terms pertaining to large language models (“language model”, “natural language processing”, “chatbot”, “ChatGPT”, “Google Bard”) and myopia (“myopia”, “near-sightedness, “refractive error”). We found that previous studies predominantly focused on assessing the competencies of LLMs in answering standardized ophthalmology specialty exams, but with a notable lack of focus on the specific topic of myopia.

**Added value of this study**

Our study presents a comparative analysis of the performance of three LLM-Chatbots in addressing 31 common myopia-related queries by patients and parents. This is a significant departure from previous research, which primarily used standardized ophthalmology exam questions.

To enhance the validity of our findings, we implemented rigorous measures in this study. First, our “ground truth” was established through consensus among three seasoned paediatric ophthalmologists, each with over seven years of sub-specialty experience. Second, prior to presenting the responses to the expert graders, we randomly shuffled the responses from all three Chatbots into three distinct rounds. Third, our experts graded the responses on separate days, allowing for a 48-h wash-out period between each grading round. This meticulous study design served to mask the identity of the Chatbots and mitigate any potential bias from the graders, addressing a significant gap observed in recent LLM-related studies.

Furthermore, beyond the evaluation of accuracy, our study extends its value by assessing the comprehensiveness of LLM-Chatbots. In addition, we also examined their self-correcting capabilities to discern if ‘further prompting’ improved response accuracy. Finally, for ‘poor’ rated responses, we pinpointed the incorrect segments in their answers and provided expert interpretations from a consultant-level paediatric ophthalmologist. Altogether, our comprehensive approach has shed new insights on the performance of LLM-Chatbots in the realm of myopia-related inquiries.

**Implications of all the available evidence**

Our research underscores the valuable role that LLM-Chatbots, specifically ChatGPT-4.0, can play in disseminating clinical information about myopia. Given their wide reach, LLM-Chatbots may potentially help to relieve the strain on healthcare resources. In addition, utilising these platforms to enhance public understanding of myopia prevention can potentially contribute to mitigating the growing myopia pandemic. However, with LLM-Chatbots in their infancy, it is imperative to provide them with tailored, domain-specific training, ensuring accurate information dissemination and averting patient misinformation.

## Introduction

Chatbots, empowered by advancements in Natural Language Processing (NLP), have emerged as promising tools in healthcare sector, demonstrating vast potential across various medical domains, including disease prevention, diagnosis, treatment, monitoring, and patient support.^1^, ^2^, ^3^ Contemporary NLP models, notably Large Language Models (LLMs), have undergone significant evolution from their traditional counterparts. Through the employment of a self-supervised learning approach, and training on an extensive pool of textual data, LLMs have advanced to generate more human-like responses.^4^^,^^5^

In this regard, LLMs have garnered significant interest in the medical landscape and early research has delivered promising results.^6^, ^7^, ^8^, ^9^ ChatGPT, a LLM created by OpenAI, demonstrated performance level at a standard approximate to the passing grade of the United States Medical Licensing Examination (USMLE), indicating its potential as an assistive tool in clinical care.^6^^,^^10^ Given its ability to generate anthropomorphic language, the role of LLMs has also been explored in aiding information provision for patients.^5^^,^^8^^,^^9^

Recently, there has been some explorations of LLMs’ performance in the realm of ophthalmology.^7^^,^^11^, ^12^, ^13^, ^14^, ^15^ Antaki et al. (2023) and Mihalache et al. (2023) evaluated the performance of ChatGPT-3.5 on Ophthalmic Knowledge Assessment Program (OKAP) examination questions and reported an encouraging score of approximately 40–50%.^7^^,^^15^ Both authors reported that ChatGPT-3.5 posted poorer performance in ophthalmology sub-speciality questions as compared with general questions. Conversely, a recent study by Momenaei et al. (2023) assessed the performance of ChatGPT-4.0 in answering questions related to the surgical treatment of retinal diseases.^16^ They reported excellent appropriateness scores, ranging from 80 to 90%, but observed relatively lower scores in terms of readability.

According to a survey in the United States, approximately 2 in 3 adults search for health information on the internet while 1 in 3 adults self-diagnose using online search engines.^17^ This is particularly common in the realm of myopia management, where patients and parents frequently resort to online sources. Given the emergence of LLMs, it is highly plausible that patients and parents will increasingly leverage on LLM-Chatbots to find myopia-related information. However, the accuracy of responses generated by LLM-Chatbots in responding to queries regarding myopia care have yet to be determined.

On the contrary to previous retrieval-based, healthcare-specific chatbots which draw information from specially curated dataset, LLMs such as ChatGPT are trained using a self-supervised approach and a diverse range of internet text.^18^^,^^19^ Although the internet provides an extensive pool of training data, the accuracy of information can be variable.^20^^,^^21^ This is particularly concerning as LLMs lack the ability to evaluate the credibility or reliability of their training data.^5^^,^^22^ Moreover, LLMs might lack domain-specific capabilities, making them susceptible to generating convincing yet potentially inaccurate responses, referred to as ‘hallucinations’.^18^^,^^22^^,^^23^ Despite the rapid advancements in LLMs, their performances within specific medical domains still require further thorough evaluation.

In this study, we aimed to evaluate and compare the performances of three publicly available LLMs, namely OpenAI's ChatGPT-3.5 and GPT-4.0, and Google's Bard, in responding to queries related to myopia care. We rigorously examined the accuracy and comprehensiveness of each LLM-Chatbot's responses. Our findings may provide valuable insights into the potential benefits and risks associated with using information from LLM-Chatbots to answer common myopia care questions.

## Methods

### Ethics

Approval from the ethics committee was not required since no patients were involved in our study.

### Study design

Our study was conducted between May 2nd 2023 and June 19th 2023 at the Ophthalmology Department at National University Hospital, National University Health System (NUHS), Singapore.

Paediatric ophthalmologists (YL, CHS, JSHL) and clinical optometrists (SY, YCT) collaborated to meticulously curate a set of 31 myopia care-related questions.^24^^,^^25^ This process began with sourcing queries from reputable online health information outlets, including the National Eye Institute, the American Academy of Ophthalmology, and the Brien Holden Vision Institute.^26^, ^27^, ^28^ Subsequently, the panel further refined these questions, selecting those they commonly encounter in a clinical setting from patients and their parents. In order to further understand the strengths and weaknesses of the LLM-Chatbots in various subject matters, questions were categorised into 6 domains—pathogenesis, risk factors, clinical presentation, diagnosis, treatment and prevention, and prognosis. From May 10th to June 13th 2023, responses to these queries were generated by using two versions of ChatGPT (version GPT-3.5 and GPT-4.0, OpenAI, California) and Google Bard (Google LLC, Alphabet Inc., California). Both ChatGPT 3.5 and Google Bard are publicly accessible at no charge, whereas ChatGPT-4.0 requires a paid subscription. The ChatGPT-4 model encompasses more parameters and computational power than its predecessor, ChatGPT-3.5.^29^ Consequently, it is conceivable that ChatGPT 4.0 could better manage more intricate queries and tasks. To validate this hypothesis, we incorporated both ChatGPT 3.5 and ChatGPT 4.0 into our evaluation.

**Fig. 1:**
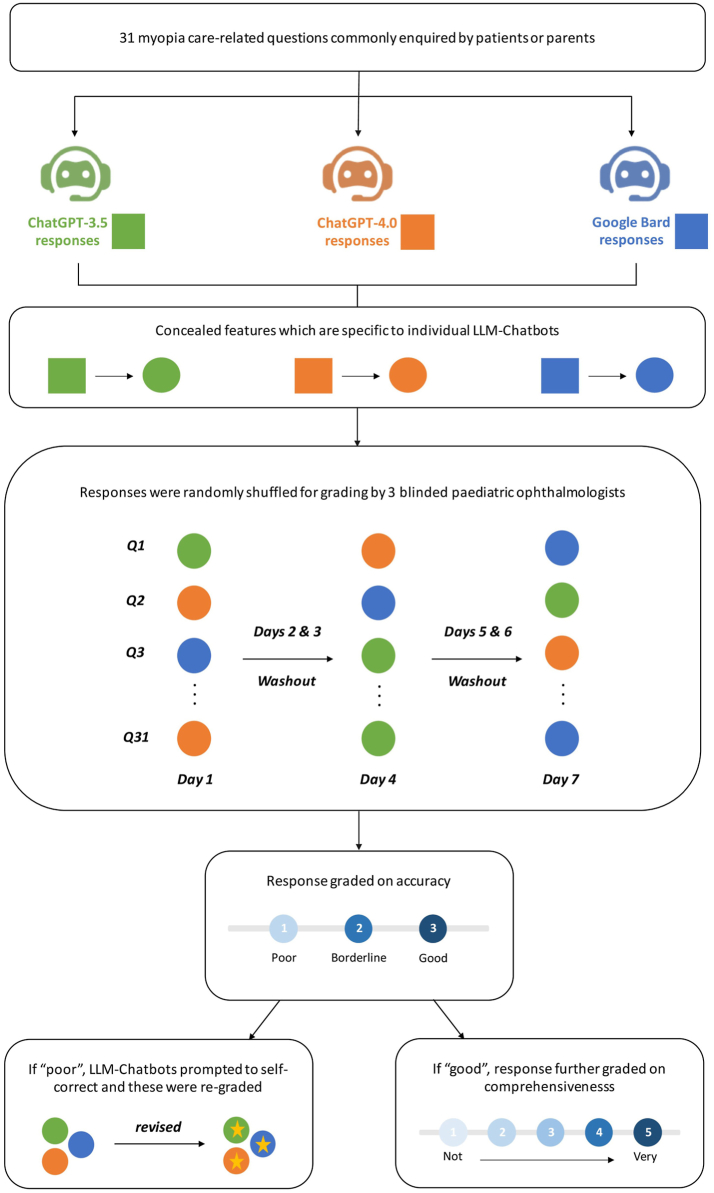
Flowchart of overall study design.

Fig. 1 illustrates the overall study design. First, a general initial prompt was used to set the context—‘I have some questions about myopia’. The 31 selected questions were then input into each LLM-Chatbot, each question was input as a ‘standalone’ query. Across all LLM-Chatbots, after each query input, the conversation was reset so as to minimise memory retention bias by the LLM-Chatbots. To ensure the graders were unable to distinguish between the different LLM-Chatbots, we formatted all generated responses into plain text, concealing any chatbot-specific features. These responses (Supplementary Tables S1a–c) were then randomly shuffled before presentation to three paediatric ophthalmologists for grading. The grading process took place over three separate rounds, each conducted on a different day with a 48-h wash-out interval in between, so as to mitigate carryover effects.

### Accuracy evaluation

The grading panel for this study comprised three experienced paediatric ophthalmologists (YL, CHS, JL), each with a minimum of seven years of practice experience in paediatric ophthalmology. The identities of the LLM-Chatbots were masked from the graders to maintain objectivity. The graders' primary task was to independently assess the accuracy of each response generated by the LLM-Chatbots, using a three-point scale as follows—1) ‘Poor’ for responses containing inaccuracies that could significantly mislead patient and potentially cause harm; 2) ‘Borderline’ for responses with possible factual errors, but unlikely to mislead or harm patient; 3) ‘Good’ for error-free responses. The sum of the scores from the three graders determined the total accuracy score for each LLM-Chatbot response (Supplementary Table S2).

We also utilized a majority consensus approach, determining the final rating for each chatbot response based on the most common grade among the three graders. In instances where a common consensus was not reached amongst the three graders (i.e., each grader provided a different rating), we defaulted to a stringent approach, assigning the lowest score (i.e., ‘poor’) to the chatbot response.

### Comprehensiveness evaluation

For chatbot responses which received a ‘good’ rating by majority consensus, the graders performed an additional evaluation to assess the comprehensiveness of these responses. For this assessment, we employed a five-point scale: 1) ‘not comprehensive’ for responses severely lacking details; 2) ‘slightly comprehensive’ for responses with minimal but basic details; 3) ‘moderately comprehensive’ for responses presenting a fair amount of detail; 4) ‘comprehensive’ for responses covering most necessary aspects; 5) ‘very comprehensive’ for responses providing exhaustive details. The overall mean comprehensiveness score was determined by averaging the scores given by each grader across the total number of ‘good’ rated responses.

### Re-evaluation of accuracy for self-corrected, revised responses from LLM-chatbots

For responses generated by the LLM-Chatbots that received a ‘poor’ rating, the LLM-Chatbots were further prompted to self-correct using this line–‘That does not seem quite right. Could you kindly review?‘. These revised responses were subsequently re-assessed for accuracy by the three graders. This re-evaluation round took place one week after the initial grading rounds. During this re-evaluation round, the graders were not informed that these responses were self-corrected versions and were blinded to the original ‘poor’ rated responses.

Additionally, we further explored ChatGPT-4.0's self-correction capabilities using the beta version of ‘Browse with Bing’ plugin.^30^ This new feature leverages on ChatGPT-4.0's capability to retrieve web-based information. In this investigation, we employed two versions of prompt to initiate self-correction: “That does not seem quite right. Could you kindly review? Please look up the web.” and “That does not seem quite right. Could you kindly review? Please look up the web for evidence-based information,”. These revised responses in response to these prompts were subsequently re-assessed for accuracy by the three graders.

### Detailed qualitative analysis of poorly-rated LLM-chatbot responses

To further shed light on the potential limitations and risks of relying solely on LLM-Chatbot responses for information about myopia, a further detailed analysis was undertaken. LLM-Chatbot responses that were rated as ‘poor’ by at least two graders underwent further scrutiny. An assigned expert (YL) meticulously identified and highlighted erroneous or inaccurate sentences within these responses, while also providing explanations for the erroneous parts.

### Statistical analysis

Statistical analyses were conducted using *R* (Version 4.1.1, R Foundation, Vienna, Austria). For comparing the differences in character count among responses across the three LLM-Chatbots, one-way ANOVA and Tukey's honest significance post-hoc test were used as the samples met parametric assumptions. For examining the differences in word count in responses, total accuracy scores, and comprehensiveness scores among the three LLMs, the Kruskal Wallis Rank Sum test and Dunn's multiple comparison post-hoc test were employed. Finally, to compare the proportions of ‘good’, ‘borderline’, and ‘poor’ ratings across the LLM-chatbots, a two-tailed Pearson's χ2 test was conducted.

When multiple hypotheses tests were conducted, p-values were adjusted using the Bonferroni correction method. A p-value of less than 0.05 was considered statistically significant.

### Role of funders

The funder of the study had no role in study design, data collection, data analysis, data interpretation, or writing of the report. All authors had full access to all the data in the study and had final responsibility for the decision to submit for publication.

## Results

Table 1 presents the length of the LLM-Chatbots’ responses to the 31 selected myopia-related questions. The mean ± standard deviation (SD) of the word count was 181.26 ± 62.05 for ChatGPT-3.5, 209.48 ± 51.35 for ChatGPT-4.0, and 247.06 ± 80.59 for Google Bard. The mean character count was 971.39 ± 345.43 for ChatGPT-3.5, 1221.13 ± 323.32 for ChatGPT-4.0, and 1275.87 ± 393.25 for Google Bard.

**Table 1:** Overview of response length from LLM-Chatbots to myopia care-related questions.

LLM	Response length (words)			Response length (characters)		
	Mean (SD)	Minimum	Maximum	Mean (SD)	Minimum	Maximum
ChatGPT-3.5	181.26 (62.05)	79	286	971.39 (345.43)	362	1555
ChatGPT-4.0	209.48 (51.35)	94	312	1221.13 (323.32)	508	1914
Google Bard	247.06 (80.59)	136	469	1275.87 (393.25)	684	2378

**Fig. 2:**
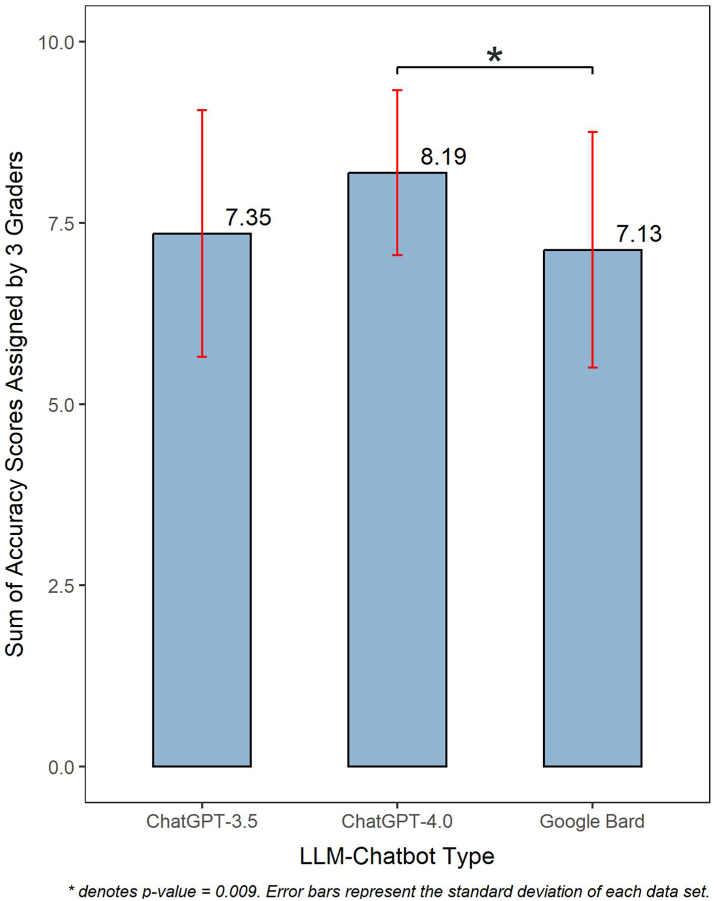
Average total accuracy scores of LLM-Chatbot responses to myopia care-related questions, as assessed by three paediatric ophthalmologists.

Fig. 2 illustrates the average total accuracy scores of LLM-Chatbots’ responses to myopia-related questions, as assessed by the three paediatric ophthalmologists. ChatGPT-4.0 demonstrated a superior average total accuracy score of 8.19 ± 1.14, surpassing both ChatGPT-3.5 (7.35 ± 1.70; Dunn's post-hoc test, p = 0.082) and Google Bard (7.13 ± 1.63; Dunn's post-hoc test, p = 0.009). Supplementary Table S2 details the total accuracy score of each LLM-Chatbot's response, attained for each question.

**Fig. 3:**
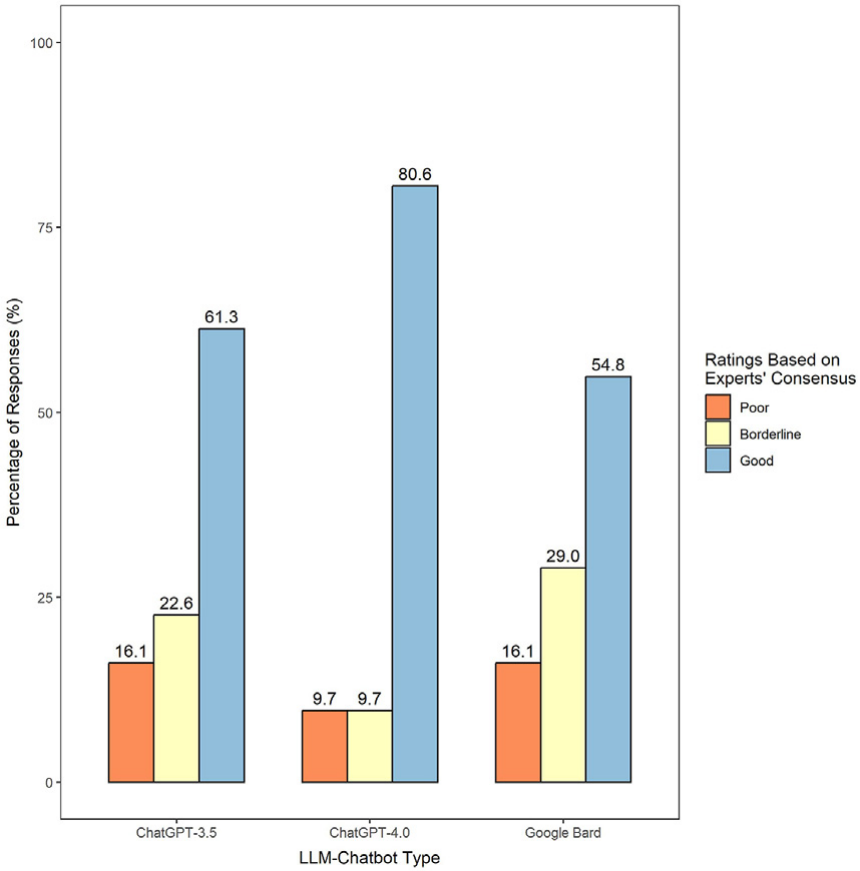
Consensus-based accuracy ratings of LLM-Chatbot responses to myopia care-related questions, as determined by three paediatric ophthalmologists.

Fig. 3 illustrates the consensus-based accuracy ratings of the three LLM-Chatbots. In ChatGPT-4.0, 80.6% of the responses were rated as ‘good’, which was significantly higher compared to 61.3% in ChatGPT-3.5 and 54.8% in Google Bard (Pearson's chi-squared test, all p ≤ 0.009). Furthermore, ChatGPT-4.0 exhibited lower proportions of responses with a ‘poor’ rating (9.7%), compared with ChatGPT-3.5 (16.1%) and Google Bard (16.1%). Supplementary Table S2 details the rating of each LLM-Chatbot's response, attained for each question.

Table 2 provides a detailed sub-analysis of the consensus-based accuracy ratings across the six myopia care domains. Overall, all three LLM-Chatbots performed consistently well in the domains of ‘clinical presentation’ and ‘prognosis’ achieving 100% ‘good’ ratings. In the ‘pathogenesis’ and ‘diagnosis’ domain, ChatGPT-3.5 and 4.0 achieved 100% ‘good’ ratings. However, Google Bard received a ‘poor’ rating for one response in each of these two domains. In the ‘treatment and prevention’ domain, all three LLM-Chatbots performed less optimally, receiving greater proportions of ‘borderline and ‘poor’ ratings in this domain. Nevertheless, ChatGPT-4.0 still performed superiorly in this domain, receiving 70% of ‘good’ ratings, compared to 40% in ChatGPT-3.5 and 45% in Google Bard (Pearson's chi-squared test, both p ≤ 0.001).

**Table 2:** Consensus-based accuracy ratings of LLM-Chatbot responses across six myopia care domains.

Domain	No. of questions	ChatGPT-3.5, n (%)			ChatGPT-4.0, n (%)			Google Bard, n (%)		
		Poor	Borderline	Good	Poor	Borderline	Good	Poor	Borderline	Good
Pathogenesis	3	0 (0)	0 (0)	3 (100)	0 (0)	0 (0)	3 (100)	1 (33)	0 (0)	2 (67)
Risk factors	2	0 (0)	0 (0)	2 (100)	0 (0)	0 (0)	2 (100)	0 (0)	1 (50)	1 (50)
Clinical presentation	1	0 (0)	0 (0)	1 (100)	0 (0)	0 (0)	1 (100)	0 (0)	0 (0)	1 (100)
Diagnosis	2	0 (0)	0 (0)	2 (100)	0 (0)	0 (0)	2 (100)	1 (50)	0 (0)	1 (50)
Treatment and prevention	20	5 (25)	7 (35)	8 (40)	3 (15)	3 (15)	14 (70)	3 (15)	8 (40)	9 (45)
Prognosis	3	0 (0)	0 (0)	3 (100)	0 (0)	0 (0)	3 (100)	0 (0)	0 (0)	3 (100)

Table 3 provides a summary of the comprehensiveness scores for ‘good’ rated responses. All three LLM-Chatbots demonstrated optimal performance in this regard. ChatGPT-3.5 scored an overall mean score of 4.11, ChatGPT-4.0 scored 4.23, and Google Bard scored 4.35 out of a maximum possible score of 5. In addition, when comparing comprehensiveness scores based on common questions responded by the three LLM-Chatbots (Table 4), similar performance was observed and there was no statistical difference across the three LLM-Chatbots (Kruskal Wallis Rank Sum test, p = 0.940).

**Table 3:** Comprehensiveness assessment for all LLM-Chatbot responses that received ‘good’ accuracy rating.

LLM	Response comprehensiveness		
	n	Mean (SD)	Median
ChatGPT-3.5	19	4.11 (0.72)	4.00
ChatGPT-4.0	25	4.23 (0.74)	4.50
Google Bard	17	4.35 (0.70)	4.50

**Table 4:** Comprehensiveness assessment for common questions answered by the three LLM-Chatbots, with responses that received ‘good’ accuracy rating.

LLM	Response comprehensiveness		
	n	Mean (SD)	Median
ChatGPT-3.5	14	4.21 (0.76)	4.42
ChatGPT-4.0	14	4.20 (0.84)	4.50
Google Bard	14	4.31 (0.77)	4.50

Table 5, Table 6, Table 7 demonstrate the LLM-Chatbots’ ability to self-correct when prompted. Overall, all LLM-Chatbots exhibited substantial self-correction capabilities. ChatGPT-3.5 improved 60% (3 out of 5) of its responses, ChatGPT-4.0 improved 66.7% (2 out of 3), and Google Bard improved 60% (3 out of 5) after self-correction prompts. Notably, each LLM-Chatbot had one response that improved from a ‘poor’ to a ‘good’ accuracy rating after self-correction. Supplementary Table S3a–c detail the original responses and the corresponding self-corrected responses for each LLM-Chatbot.

**Table 5:** Demonstration of ChatGPT-3.5's ability to self-correct when prompted.

Topic	Question	Summed score		Consensus-based rating	
		Initial	Self-Corrected	Initial	Self-Corrected
Treatment and prevention	5. How effective is MiSight/MiyoSmart/Stellest/Abiliti/orthokeratology lenses at helping to prevent myopia/myopia progression?	3	4	Poor	Poor
	6. How does MiSight/MiyoSmart/Stellest/Abiliti/orthokeratology lenses help to prevent myopia/myopia progression?	3	3	Poor	Poor
	11. What type of diet/vitamin supplementation can help to prevent myopia/myopia progression?	6	8	Poor^a^	Good
	18. How does atropine help to prevent myopia/myopia progression?	6	7	Poor^a^	Borderline
	19. My child has not developed myopia, should he/she start using atropine?	6	7	Poor^a^	Borderline

a Where consensus on final accuracy rating was not reached (i.e. each grader provided a different rating), the lowest score (‘*poor*’) was assigned.

**Table 6:** Demonstration of ChatGPT-4.0's ability to self-correct when prompted.

Topic	Question	Summed score		Consensus-based rating	
		Initial	Self-Corrected	Initial	Self-Corrected
Treatment and prevention	4. What are the spectacles/contact lenses available to prevent myopia/myopia progression?	6	5	Poor^a^	Borderline
	11. What type of diet/vitamin supplementation can help to prevent myopia/myopia progression?	5	9	Poor	Good
	19. My child has not developed myopia, should he/she start using atropine?	6	6	Poor^a^	Poor^a^

a Where consensus on final accuracy rating was not reached (i.e. each grader provided a different rating), the lowest score (*‘poor’)* was assigned.

**Table 7:** Demonstration of Google Bard's ability to self-correct when prompted.

Topic	Question	Summed score		Consensus-based rating	
		Initial	Self-Corrected	Initial	Self-Corrected
Pathogenesis	1. What is myopia?	4	7	Poor	Borderline
Diagnosis	2. What should I do if I suspect my child has myopia?	6	8	Poor^a^	Good
Treatment and prevention	11. What type of diet/vitamin supplementation can help to prevent myopia/myopia progression?	5	6	Poor	Borderline
	12. Are there any available vision therapy/eye exercises that can help to prevent myopia/myopia progression?	4	3	Poor	Poor
	13. What medications are available to prevent myopia/myopia progression?	4	4	Poor	Poor

a Where consensus on final accuracy rating was not reached (i.e. each grader provided a different rating), the lowest score (‘*poor*’) was assigned.

Supplementary Table S4a–c feature examples of erroneous responses generated by the LLM-Chatbots. The specific portions of the responses that contain errors are highlighted in yellow. Additionally, these tables also provide further explanations for the identified errors, with inputs contributed by a paediatric ophthalmologist (YL).

## Discussion

Our study presents a rigorous evaluation of ChatGPT-3.5, ChatGPT-4.0, and Google Bard in addressing myopia-related queries frequently asked by patients or parents. By employing a robust study design with appropriate masking and randomisation, and meticulous reviews by three experienced, consultant-level paediatric ophthalmologists, we further strengthened the integrity of our assessment. Our findings revealed that LLM-Chatbots, particularly ChatGPT-4.0, have the potential to deliver accurate and comprehensive responses to myopia-related queries. Furthermore, we obtained unique insights into LLMs' self-correcting abilities to improve accuracy in their responses when prompted. To the best of our knowledge, our study is among the few that have evaluated this aspect to date. However, it is noteworthy that these LLMs demonstrated weaker performance when handling inquiries pertaining to myopia treatment and prevention. Our study pioneers the exploration of LLM utility in the field of ophthalmology, specially focussing on common inquiries related to myopia care. Unlike previous research that largely focused on evaluations through standardized exams,^7^^,^^15^^,^^31^ our study delves into realistic scenarios where concerned parents may seek assistance through these emerging resources. This underscores the importance of assessing the accuracy and validity of responses delivered by LLM-Chatbots in such real-world context. Taken together, our findings have profound implications, possibly paving the way for incorporating LLM-Chatbots into myopia care management.

Among the three evaluated LLM-Chatbots, ChatGPT-4.0 emerged as the most proficient in addressing myopia-related queries. It achieved the highest average accuracy score and received considerably higher proportions of ‘good’ ratings compared to the other two LLMs (Fig. 2, Fig. 3). This was also evident across all six domains (Table 2). Our findings echo prior studies by Ali et al. (2023) and Raimondi et al. (2023) that underlined the edge of ChatGPT-4.0 over other LLM counterparts in neurosurgery and ophthalmology exams, respectively.^31^^,^^32^ Its superior performance may be attributed to several factors unique to ChatGPT-4.0, such as its hugely expansive parameter set, substantial users and collaborating experts providing ongoing feedback to inform its training, its advanced reasoning and instruction-following capabilities, more recent training data, and integrating insights gained from practical application of those previous models into GPT-4.0's safety research and monitoring system, all of which likely resulted in ChatGPT-4.0 delivering more accurate responses.^33^^,^^34^ Interestingly, however, all three LLM-Chatbots were similarly competent in providing comprehensive responses. Supplementary Table S5 illustrates an example where all three Chatbots provided comprehensive answers when responding to the query “How much outdoor time does my child require to prevent myopia/myopia progression?”. This further attested to the LLM-Chatbots’ abilities to offer pertinent and detailed information.

Across the six question domains, when responding to inquiries concerning other well-established information, such as signs and symptoms, as well as disease outcomes, all LLM-Chatbots exhibited stellar performance, achieving perfect scores in domains such as ‘clinical presentation’ and 'prognosis' (Supplementary Table S2). However, it was notable that all three LLM-Chatbots exhibited the least robust performance when addressing queries related to the ‘treatment and prevention’ domain (Table 2). This finding may be attributed to the evolving landscape of myopia treatment and the potential limitations of the LLM-Chatbots’ training data, which might not be fully aligned with the latest advancements in this field. Consequently, the accuracy of the LLM-Chatbots' responses was notably diminished in this domain.

An illustrative example was the failure of LLM-Chatbots to consider recent findings by Yam et al. (2023) supporting the administration of atropine for myopia prophylaxis^35^ when answering the question, ‘My child has not developed myopia, should he/she start using atropine?’ (Question 19, ‘Treatment and Prevention’ domain, Supplementary Tables S1a–c). Consequently, all three LLM-Chatbots received final accuracy scores of either ‘poor’ or ‘borderline’ (Supplementary Table S2).

There were also other instances where all three LLM-Chatbots performed suboptimally, earning either ‘poor’ or ‘borderline’ accuracy scores due to the dissemination of misinformation. This was notably the case with the question, ‘What type of diet/vitamin supplementation can help prevent myopia/myopia progression?’ (Question 11, ‘Treatment and Prevention’ domain, Supplementary Tables S1a–c). Despite ongoing research yielding inconclusive evidence about the correlation between diet, supplements, and the prevention of myopia or myopia progression,^36^, ^37^, ^38^, ^39^ all three LLM-Chatbots recommended the consumption of supplements such as omega-3 fatty acids (Supplementary Tables 1a–c, 4b and c). These observations highlight the risk of LLM-Chatbots providing misinformation to unsuspecting patients, while indicating their limited ability to identify and rectify such instances.

Expanding on the issue of misinformation, we further present a significant example. In assessing the effectiveness of various optical interventions for myopia management (Question 5, ‘Treatment and Prevention’ domain), ChatGPT-3.5 poorly advised and received a ‘poor’ rating from the graders. This is because the optical interventions, such as HOYA MiyoSmart and Essilor Stellest, are well-validated through randomized controlled trials with substantial efficacy data available.^40^^,^^41^ However, ChatGPT-3.5 inaccurately presented MiyoSmart as a soft contact lens with a concentric ring design and provided an incorrect description of Stellest's visual capabilities (Supplementary Table S4a). In truth, MiyoSmart is a spectacle lens with multiple defocus segments, and Stellest features a highly aspherical lenslet design in its periphery.^42^^,^^43^ Furthermore, ChatGPT-3.5 erroneously claimed that Abiliti is an implantable device requiring surgical intervention, while in fact, Abiliti is a contact lens for myopia control available in soft and hard (orthokeratology) forms, without requiring surgical implantation.^44^^,^^45^ The spread of such misinformation by LLM-Chatbots can mislead users, potentially hindering effective myopia management. This further highlights the importance of accurate and reliable information dissemination from LLM-Chatbots.

Interestingly, however, ChatGPT-4.0 still outperformed ChatGPT-3.5 and Google Bard even in the domain of ‘treatment and prevention’, obtaining 70% ‘good’ ratings. This underlines ChatGPT-4.0's consistent superiority across a broad spectrum of evaluated questions. For instance, in response to the question “What medications are available to prevent myopia/myopia progression?” (Question 13, treatment and prevention domain), ChatGPT-4.0 received unanimous ‘good’ ratings from all three graders (Supplementary Table S2). Conversely, ChatGPT-3.5 was deemed ‘borderline’ due to its erroneous statement that “the use of atropine eyedrop is relatively new and that the optimal dosage has yet to be established” (Supplementary Table S1a). On the other hand, Google Bard received a ‘poor’ rating for providing inaccurate and potentially harmful advice by indicating “the use of pilocarpine eye drops to control myopia” (Supplementary Table S1c). It is important to note that, compared to other domains, the ‘treatment and prevention’ domain likely demands more recent training data, considering the fast-evolving landscape of myopia treatment. In this context, ChatGPT-4.0 has demonstrated superior capacity to manage complex queries as such relative to its counterparts.

Across the other five question domains, Google Bard showed a noticeable underperformance relative to the other LLM models, especially in the ‘pathogenesis,’ ‘risk factors,’ and ‘diagnosis’ domains. In these domains, Google Bard garnered more ‘borderline’ or ‘poor’ scores for several queries, while the other two LLM models consistently delivered ‘good’ responses (Supplementary Table S2). Noteworthy, the queries within these domains largely required straightforward factual recall, such as the query defining myopia. However, Google Bard inaccurately defined axial myopia and omitted mention of potential complications associated with refractive surgeries when proposing them as myopia correction solutions (Supplementary Table S4c). Such misinformation could misguide patients and potentially lead to adverse outcomes.

Each LLM-Chatbot has demonstrated the ability to self-correct, notably improving the accuracy of responses initially deemed ‘poor’ in some cases. These revisions were achieved solely through a straightforward prompt, without explicit guidance towards the correct answer. To the best of our knowledge, this study is the first to systematically evaluate the self-correction capabilities of LLM-Chatbots within the context of myopia care. While the observed improvements in the transition of responses from ‘poor’ to ‘good’ (with one such example in each LLM-Chatbot) may not be significant, they underline the present capacity of LLMs to acknowledge potential inaccuracies when prompted and make attempts at self-correction (Table 5, Table 6, Table 7). We anticipate that these ‘self-correction’ capabilities will enhance over time as user feedback continues to inform the evolvement of these LLMs. However, the dependence on user feedback introduces risks, as this naturally places considerable amount of reliance on the users' integrity, knowledge, and potential biases.^46^ Additionally, the lack of an automatic disclaimer from these LLM-Chatbots, even when responses remained ‘poor’ after self-correction, emerged as a significant concern (Supplementary Tables S3a–c). This implies that despite attempts at self-correction, LLM-Chatbots could still potentially disseminate inaccurate medical information without acknowledging its inherent uncertainty.

Despite the integration of the Bing search engine in ChatGPT-4.0 for web search functionality, we observed minimal improvement in the self-corrected responses (Question 4 and 19, ‘Treatment and Prevention’ domain, Supplementary Table S6). Regardless of the prompt's nature, whether requesting for simple web search or asking for evidence-based answers—the beta version of ChatGPT-4.0's ‘Browse with Bing’ consistently scored ‘borderline’ accuracy for both questions. Notably, the LLM-Chatbot consistently substantiated its answers with peer-reviewed articles (Supplementary Table S7). However, the relevance of the chosen articles fell short, resulting in no significant improvement in response accuracy. For instance, while responding to the question, “Should my non-myopic child start using atropine?”, the LLM-Chatbot referred to an outdated, small-scale study by Fang et al. (year 2010),^47^ neglecting to cite more relevant, recent research like Yam et al.^35^ Similarly, another response cited a report by the American Academy of Ophthalmology^48^ regarding treatment trials in myopic children, but was irrelevant to the original which pertained to initiating atropine in pre-myopic children. While the ability of ChatGPT-4.0 to conduct web searches and provide transparent links to supporting articles is interesting, the parameters governing the selection of these online articles remain unclear and necessitate further investigation.

Our findings highlight the potential utility of LLM-Chatbots in the provision of information clinically. Given the rapidly growing exploration and use of LLM-Chatbot worldwide, these could serve as essential platforms for information dissemination. This is further strengthened by recent advancements in ChatGPT-4.0, which now offers API access.^49^ Utilising this API-enabled integration, users can seamlessly integrate ChatGPT's natural language processing capabilities into diverse online services. This sets the stage for the prospective creation of a myopia-focused chatbot, grounded in the advanced architecture of ChatGPT-4.0. The increasing accessibility and availability of information through LLM-Chatbots regarding myopia prevention could help mitigate the growing myopia pandemic, however, one critical concern that needs to be addressed with LLMs is their limited capacity to recognise and prevent potential misinformation. Nonetheless, until LLM-Chatbots develop more sophisticated critical analysis skills, their use is a double-edged sword and must be approached with caution.

Importantly, the role of LLMs potentially extend beyond Ophthalmology to other medical specialities.^50^, ^51^, ^52^, ^53^, ^54^, ^55^, ^56^, ^57^, ^58^ However, the weaknesses and strengths of LLMs may differ across different medical specialities. For instance, Rasmussen et al. (2023) demonstrated poorer ChatGPT-3.5 performance with treatment- and prevention-related questions pertaining to vernal keratoconjunctivitis.^50^ On the other hand, Lahat et al. (2023) observed poorer performance of ChatGPT-3.5 in diagnosis-related questions pertaining to gastrointestinal health.^57^ The varying performance of LLMs may be attributed to the differing depth of information available on each topic on the internet. Given that ChatGPT was trained on internet data available up until September 2021, the model's proficiency is reflective of the knowledge, perspectives, and biases found within the dataset. Nonetheless, LLMs are progressing highly rapidly. This is demonstrated by Johnson et al. (2023), who reported a significant increase in the accuracy scores of cancer-related information within a mere two-week interval between evaluations.^56^ Taken together, the performance and pitfalls of LLMs still require thorough evaluation across different medical topics.

The strengths of our study lie in its robust design which included masking LLM-specific features in responses, random shuffling of responses before presentation to graders, and implementing wash-out periods between grading days. These measures helped to minimise bias from graders and further strengthened the validity of our conclusions. However, this study is not without caveats. Firstly, the subjectivity by individual graders when assigning ratings for accuracy and comprehensiveness cannot be overlooked. However, we mitigated this by selecting three highly experienced consultant-level paediatric ophthalmologists (>7 years of expertise), and by adopting the consensus-based rating approach. Second, across the 6 domains, there was an unequal distribution of queries across the categories, with 62.5% (20 out of all 31 questions) pertaining to ‘treatment and prevention’. Therefore, caution must be exercised when interpreting the performance of LLM-Chatbots on domains encompassing a limited number of questions. Lastly, it is imperative to consider that as LLMs constantly adapt and evolve via user feedback and iterative training set updates, these results should be interpreted within the scope of their respective time frames. Consequently, future investigations might yield varied outcomes.

In conclusion, our study revealed that ChatGPT-4.0 outperformed both ChatGPT-3.5 and Google Bard in responding to common myopia-related queries. This comparative analysis provided a nuanced understanding of the accuracy across different LLM-Chatbots and underscored the promising potential of ChatGPT-4.0 in delivering accurate and comprehensive information regarding myopia care. Continuous exploration of strategies and evaluations to further refine and ascertain the efficacy of these tools will be paramount moving forward.

## Contributors

ZWL, KP, SMEY, and Y-CT contributed to conception of the study.

ZWL, KP, SMEY, and Y-CT contributed to study design.

ZWL, KP, SMEY, and Y-CT contributed to acquisition of the data.

KP contributed to statistical analysis of data.

ZWL, KP, SMEY, and Y-CT contributed to analysis and interpretation of the data.

KP, SMEY and Y-CT accessed and verified each dataset during the course of the study.

Supervision of this research which includes responsibility for the research activity planning and execution was oversighted by Y-CT.

KP contributed to visualisation which includes figure, charts and tables of the data.

All authors had access to all the data and ZWL, KP, SMEY and Y-CT were responsible for the decision to submit the manuscript.

ZWL, KP, SMEY and Y-CT drafted the manuscript.

All authors read and approved the final version of the manuscript.

## Data sharing statement

We have ensured that all the essential data necessary for replicating our results is included in our Supplementary file. The only exception is the raw scores assigned by individual graders, which can be provided upon request.

## Declaration of generative AI and AI-assisted technologies in the writing process

During the preparation of this work, the authors used ChatGPT to edit and proofread the manuscript for improved readability. After using this tool/service, the authors reviewed and edited the content as needed and take full responsibility for the content of the publication.

## Declaration of interests

All authors declare no competing interests.
