# Supplementary material for: Benchmarking large language models’ performances for myopia care: a comparative analysis of ChatGPT-3.5, ChatGPT-4.0, and Google Bard
Source: eBioMedicine. 2023 Aug 23;95:104770. doi: 10.1016/j.ebiom.2023.104770 (PMC10470220; doi:10.1016/j.ebiom.2023.104770)
Supplement: Supplementary Tables [file mmc1.docx]

Supplementary File for

Benchmarking Chatbot Performance for Myopia Care: A Comparative Analysis of ChatGPT-3.5, ChatGPT-4.0, and Google Bard

**This PDF file includes:**

- Supplementary Tables 1 to 7

# Supplementary Table 1a: ChatGPT-3.5 Responses to Myopia-Related Questions

| **Question** | **ChatGPT-3.5 Response** |
| --- | --- |
| **Topic: Pathogenesis** | |
| 1. **What is myopia?** | Myopia, also known as nearsightedness, is a common refractive error of the eye that causes distant objects to appear blurry. In myopia, the eyeball is typically longer than normal, which causes light to focus in front of the retina instead of directly on it. This can result in blurred vision when looking at objects in the distance, while objects that are closer may still appear clear. Myopia can occur in both children and adults and may worsen over time if left untreated. |
| 1. **At what age does myopia commonly develop?** | Myopia can develop at any age, but it usually starts in childhood and tends to progress until the late teenage years or early adulthood. The onset of myopia typically occurs between 6 and 14 years of age, with most cases appearing between the ages of 8 and 12. However, myopia can also develop in adulthood, although this is less common. It's important to note that the age of onset and progression of myopia can vary from person to person. |
| 1. **What causes myopia?** | The exact cause of myopia is not fully understood, but research suggests that both genetic and environmental factors can contribute to its development.   1. **Genetic factors**: There is strong evidence that myopia has a genetic component, meaning it can run in families. Children with one myopic parent have a three-fold increased risk of developing myopia, while those with two myopic parents have a six-fold increased risk. 2. **Environmental factors**: Environmental factors that are associated with the development of myopia include spending long periods of time on near work (such as reading or using a computer), insufficient outdoor exposure, and certain lifestyle factors such as lack of physical activity or inadequate sleep.   Additionally, factors such as ethnicity and urbanization have also been linked to an increased risk of myopia. |
| **Topic: Risk Factors** | |
| 1. **What puts my child at risk of myopia?** | Several factors can put a child at an increased risk of developing myopia, including:   1. **Family history**: Children with one myopic parent have a three-fold increased risk of developing myopia, while those with two myopic parents have a six-fold increased risk. 2. **Ethnicity**: Certain ethnic groups, such as East Asians, have a higher prevalence of myopia compared to other ethnic groups. 3. **Age**: Myopia tends to develop and progress during childhood and adolescence, so younger children are at a higher risk. 4. **Near work and lack of outdoor activity**: Spending long periods of time on near work (such as reading or using electronic devices) and insufficient outdoor exposure are associated with an increased risk of myopia. 5. **Urbanization**: Children who live in urban areas may have a higher risk of myopia than those who live in rural areas. 6. **Low birth weight**: Some studies have suggested that low birth weight and premature birth may be associated with an increased risk of myopia.   It is important to note that having one or more of these risk factors does not necessarily mean that a child will develop myopia, but they may increase the likelihood. |
| 1. **Is myopia hereditary? My spouse and I have myopia, how likely is it for my child to get myopia?** | Yes, myopia has a strong genetic component, which means that it can be hereditary. If both you and your spouse have myopia, your child is more likely to develop myopia compared to a child whose parents do not have myopia.  The risk of developing myopia is higher if both parents have myopia compared to if only one parent has it. For example, if one parent is myopic, the child has a 25% chance of developing myopia. If both parents are myopic, the child's risk increases to 50-60%.  However, it is important to note that myopia is a complex condition that is influenced by both genetic and environmental factors. Even if a child has a genetic predisposition to myopia, there are still steps that can be taken to reduce the risk of developing myopia or slow down its progression. This includes spending more time outdoors, limiting near work activities, and using appropriate eyewear when needed. |
| **Topic: Clinical Presentation** | |
| 1. **What are signs and symptoms that suggests my child has myopia?** | Some common signs and symptoms that may suggest that a child has myopia include:   1. **Difficulty seeing objects in the distance**: Children with myopia may have trouble seeing objects such as road signs or the chalkboard in school when they are far away. 2. **Squinting**: Squinting can help to temporarily improve vision in people with myopia, so your child may squint when trying to see things in the distance. 3. **Sitting too close to the TV or computer screen**: Children with myopia may sit too close to the TV or computer screen to see things more clearly. 4. **Headaches or eye strain**: Straining the eyes to see things in the distance can cause headaches or eye strain, which may be a sign of myopia. 5. **Rubbing the eyes**: Children with myopia may rub their eyes frequently due to eye strain or fatigue. 6. **Difficulty seeing in low light**: Myopia can make it harder to see in low light conditions, such as in a dimly lit room.   If you notice any of these signs or symptoms in your child, it is important to schedule an eye exam with an optometrist or ophthalmologist. Early detection and management of myopia can help to prevent its progression and reduce the risk of associated complications. |
| **Topic: Diagnosis** | |
| 1. **How is myopia diagnosed?** | Myopia is typically diagnosed through a comprehensive eye exam conducted by an optometrist or ophthalmologist. The exam will usually include several tests to assess the child's vision and eye health, including:   1. **Visual acuity test**: This measures how well the child can see letters or numbers on an eye chart at a specific distance. 2. **Refraction test**: This helps to determine the degree of myopia by measuring how the child's eyes focus light. 3. **Retinal exam**: This involves examining the back of the eye to check for signs of myopia-related complications, such as retinal detachment or macular degeneration. 4. **Eye alignment and eye movement testing**: This assesses how well the eyes work together and how well they move and track objects.   If the eye exam indicates that your child has myopia, the optometrist or ophthalmologist will usually prescribe corrective eyeglasses or contact lenses to help improve vision. The prescription may need to be updated regularly as the child's myopia progresses. In some cases, other treatments such as orthokeratology, atropine eye drops, or multifocal contact lenses may also be recommended to slow down the progression of myopia. |
| 1. **What should I do if I suspect my child has myopia?** | If you suspect that your child has myopia, it is important to schedule an eye exam with an optometrist or ophthalmologist as soon as possible. Early detection and management of myopia can help to prevent its progression and reduce the risk of associated complications.  During the eye exam, the eye doctor will assess your child's vision and eye health and may perform tests such as a visual acuity test, refraction test, and retinal exam. If myopia is diagnosed, the eye doctor will prescribe corrective eyeglasses or contact lenses to help improve your child's vision. They may also provide recommendations on lifestyle changes or other treatments to slow down the progression of myopia.  In addition to scheduling regular eye exams for your child, there are several steps you can take to help prevent or slow down the progression of myopia, including:   1. **Encourage outdoor activities**: Spending time outdoors has been shown to be effective in reducing the risk of myopia development and progression in children. 2. **Limit screen time**: Excessive screen time, particularly on handheld devices, has been linked to an increased risk of myopia. Encourage your child to take frequent breaks from screen time. 3. **Ensure proper lighting**: Make sure that your child's study area and other spaces they spend time in are well-lit to reduce eye strain. 4. **Encourage good posture**: Encourage your child to maintain good posture when reading or doing other near work to reduce the risk of eye strain.   Follow your eye doctor's recommendations: Work with your child's eye doctor to determine the best course of treatment and follow their recommendations for managing myopia. |
| **Topic: Treatment and Prevention** | |
| 1. **What can I do to prevent myopia?** | While myopia cannot be completely prevented, there are several steps you can take to reduce the risk of its development or progression:   1. **Encourage outdoor activities**: Spending time outdoors has been shown to be effective in reducing the risk of myopia development and progression in children. 2. **Limit screen time**: Excessive screen time, particularly on handheld devices, has been linked to an increased risk of myopia. Encourage your child to take frequent breaks from screen time. 3. **Ensure proper lighting**: Make sure that your child's study area and other spaces they spend time in are well-lit to reduce eye strain. 4. **Encourage good posture**: Encourage your child to maintain good posture when reading or doing other near work to reduce the risk of eye strain. 5. **Regular eye exams**: Schedule regular eye exams for your child to detect and manage myopia early on. 6. **Proper nutrition**: Eating a balanced diet that includes foods rich in vitamin A, such as leafy greens, carrots, and sweet potatoes, may help to maintain good eye health. 7. **Control near work activities**: Encourage children to take breaks during near work activities and maintain an appropriate reading distance.   It's important to note that these steps may not completely prevent myopia, but they can help to reduce the risk of its development or slow down its progression. If you suspect that your child has myopia, schedule an eye exam with an optometrist or ophthalmologist as soon as possible. |
| 1. **My child has myopia, what are the available treatments to slow down the progression of myopia?** | There are several treatments available that can help to slow down the progression of myopia in children. These include:   1. **Orthokeratology**: Also known as ortho-k, this is a non-surgical treatment that involves wearing specially designed gas permeable contact lenses overnight. These lenses reshape the cornea temporarily, providing clear vision during the day without the need for corrective lenses. Ortho-k has been shown to be effective in slowing down the progression of myopia in children. 2. **Multifocal contact lenses**: These are specialized contact lenses that have different prescriptions in different areas of the lens. This can help to reduce the strain on the eyes during close work and slow down the progression of myopia. 3. **Atropine eye drops**: Atropine is a medication that can be applied as eye drops to dilate the pupils and relax the focusing mechanism of the eyes. This can help to reduce the progression of myopia in children. The side effects of atropine, such as light sensitivity and blurred vision, are typically mild and temporary. 4. **Prescription eyeglasses or contact lenses**: These are the most common treatments for myopia and are used to correct the child's vision. However, they do not slow down the progression of myopia.   It's important to note that the most appropriate treatment option for your child will depend on several factors, such as the severity of their myopia, their age, and their lifestyle. Talk to an optometrist or ophthalmologist to determine the best course of treatment for your child. They can provide you with more information about the available options and help you make an informed decision. |
| 1. **How can I prevent myopia/ myopia progression without the use of medications?** | There are several non-medication strategies that can be used to prevent myopia progression in children:   1. **Spend time outdoors**: Studies have shown that spending more time outdoors can reduce the risk of myopia development and progression in children. Encourage your child to spend at least two hours per day outdoors. 2. **Limit screen time**: Excessive screen time, particularly on handheld devices, has been linked to an increased risk of myopia. Encourage your child to take frequent breaks from screen time. 3. **Good lighting and proper posture:** Ensure proper lighting when your child is reading or doing close work to reduce eye strain. Encourage good posture when reading or doing other near work to reduce the risk of eye strain. 4. **Maintain an appropriate reading distance**: Encourage your child to maintain a distance of at least 30 cm between their eyes and the reading material. 5. **Control near work activities**: Encourage children to take breaks during near work activities, such as reading or studying, to reduce eye strain. 6. **Proper nutrition**: Eating a balanced diet that includes foods rich in vitamin A, such as leafy greens, carrots, and sweet potatoes, may help to maintain good eye health. 7. **Regular eye exams**: Schedule regular eye exams for your child to detect and manage myopia early on.   It's important to note that these strategies may not completely prevent myopia or stop its progression, but they can help to reduce the risk of its development or slow down its progression. Consult with an optometrist or ophthalmologist for more information on the appropriate strategies to use for your child based on their specific situation. |
| 1. **What are the spectacles/contact lenses available to prevent myopia/ myopia progression?** | There are several types of eyeglasses and contact lenses that can be used to correct vision and potentially slow down the progression of myopia in children. These include:   1. **Multifocal lenses**: These eyeglasses or contact lenses have different prescriptions in different areas of the lens. The lens is designed to correct distance vision, while also providing some near vision correction. Studies have shown that multifocal lenses can be effective in slowing down the progression of myopia in children. 2. **Orthokeratology (Ortho-k) lenses**: These are specialized gas permeable contact lenses that are worn overnight to temporarily reshape the cornea. The lenses correct vision during the day without the need for eyeglasses or contact lenses. Ortho-k lenses have been shown to be effective in slowing down the progression of myopia in children. 3. **Soft multifocal contact lenses**: These are contact lenses that have different prescriptions in different areas of the lens. Like multifocal eyeglasses, they can help to reduce eye strain during close work and slow down the progression of myopia. 4. **Single-vision lenses**: These are traditional eyeglasses or contact lenses that have the same prescription throughout the lens. While they do not slow down the progression of myopia, they are still an effective way to correct vision.   It's important to note that the most appropriate type of eyeglasses or contact lenses for your child will depend on several factors, such as the severity of their myopia, their age, and their lifestyle. Consult with an optometrist or ophthalmologist to determine the best course of treatment for your child. They can provide you with more information about the available options and help you make an informed decision. |
| 1. **How effective is MiSight/ MiyoSmart/ Stellest/ Abiliti/ orthokeratology lenses at helping to prevent myopia/ myopia progression?** | MiSight, MiyoSmart, Stellest, Abiliti, and orthokeratology lenses are all options that have been shown to be effective in slowing down the progression of myopia in children.  **MiSight**: MiSight is a soft contact lens that is specifically designed to correct myopia and slow down its progression in children. Clinical studies have shown that it can slow the progression of myopia by up to 59% over three years.  **MiyoSmart**: MiyoSmart is another soft contact lens designed to correct myopia and slow down its progression. It features a unique concentric ring design that has been shown to be effective in slowing down myopia progression by up to 60%.  **Stellest**: Stellest is a spectacle lens that has a central area for distance vision and a peripheral area for near vision. Clinical studies have shown that it can slow down myopia progression by up to 67% over two years.  **Abiliti**: Abiliti is an implantable device that is surgically implanted in the eye. It uses low-level electrical pulses to help slow down the progression of myopia. Clinical studies have shown that it can reduce myopia progression by up to 59% over two years.  **Orthokeratology lenses**: Orthokeratology (Ortho-k) lenses are gas permeable contact lenses that are worn overnight to temporarily reshape the cornea. Studies have shown that they can reduce myopia progression by up to 50%.  It's important to note that the effectiveness of these treatments can vary depending on several factors, such as the severity of the myopia, the age of the child, and the length of treatment. Consult with an optometrist or ophthalmologist to determine the most appropriate treatment option for your child based on their specific situation. They can provide you with more information about the available options and help you make an informed decision. |
| 1. **How does MiSight/ MiyoSmart/ Stellest/ Abiliti/ orthokeratology lenses help to prevent myopia/ myopia progression?** | MiSight, MiyoSmart, Stellest, Abiliti, and orthokeratology lenses all work in different ways to help prevent myopia or slow down its progression.  **MiSight and MiyoSmart**: These are soft contact lenses that have a unique concentric ring design. The rings create a distance center and a peripheral zone that provides myopic defocus, which has been shown to slow down the elongation of the eye that causes myopia progression. The lenses also correct the child's vision while they wear them.  **Stellest**: Stellest is a spectacle lens that also uses myopic defocus to slow down myopia progression. The lens has a central area for distance vision and a peripheral area for near vision. The peripheral area is designed to create myopic defocus, which has been shown to slow down the elongation of the eye that causes myopia progression.  **Abiliti**: Abiliti is an implantable device that is surgically implanted in the eye. It uses low-level electrical pulses to stimulate the ciliary muscle, which controls the shape of the lens in the eye. This stimulation can help to reduce the elongation of the eye that causes myopia progression.  **Orthokeratology lenses**: Orthokeratology (Ortho-k) lenses are gas permeable contact lenses that are worn overnight to temporarily reshape the cornea. The reshaping of the cornea can help to correct myopia and slow down its progression. The lenses are worn overnight and removed in the morning, providing clear vision during the day without the need for eyeglasses or contact lenses.  Overall, these treatments all aim to provide clear vision while also creating myopic defocus, which has been shown to slow down the elongation of the eye that causes myopia progression. Consult with an optometrist or ophthalmologist to determine the most appropriate treatment option for your child based on their specific situation. |
| 1. **Is it safe for my child to be using myopia control contact lens at such a young age?** | Myopia control contact lenses such as MiSight, MiyoSmart, and orthokeratology lenses have been shown to be safe and effective for children. However, it is important to note that all contact lenses, including those used for myopia control, carry some risks.  The safety of contact lenses depends on proper fitting, hygiene, and use. Children may need additional education and supervision to ensure proper handling, cleaning, and storage of the lenses. It is essential to follow the instructions of the eye care professional regarding the use and care of the lenses.  If you are considering myopia control contact lenses for your child, consult with an optometrist or ophthalmologist who specializes in pediatric eye care. They will assess your child's eye health, determine the best treatment option for their specific situation, and provide guidance on proper use and care of the lenses to ensure their safety. |
| 1. **How effective is reducing screen time/ increasing outdoor time at helping to prevent myopia/ myopia progression?** | Reducing screen time and increasing outdoor time have been suggested as possible ways to help prevent myopia or slow down its progression, but the evidence on their effectiveness is not conclusive.  Some studies have suggested that spending more time outdoors, particularly in natural light, may help to prevent myopia or slow down its progression in children. The exact reasons for this are not fully understood, but it may be related to the increased levels of natural light, exposure to different visual stimuli, and the relaxation of the eye muscles that occurs when looking at distant objects.  On the other hand, there is limited evidence to support the effectiveness of reducing screen time in preventing myopia or slowing down its progression. However, some eye care professionals recommend taking regular breaks from screen time to reduce eye strain and fatigue.  It is important to note that myopia is a complex condition that is influenced by both genetic and environmental factors, and there is no guaranteed way to prevent it. While reducing screen time and increasing outdoor time may have some benefits, they should not be relied on as the sole method for preventing myopia or slowing down its progression. It is essential to consult with an optometrist or ophthalmologist who specializes in pediatric eye care to determine the most appropriate treatment plan for your child based on their specific situation. |
| 1. **What is the maximum duration of screen time/ near work per day before my child is at risk of myopia/ myopia progression?** | There is no clear-cut maximum duration of screen time or near work that puts a child at risk for myopia or myopia progression. Myopia is a complex condition that is influenced by both genetic and environmental factors, and the relationship between screen time or near work and myopia is not fully understood.  While there is some evidence to suggest that excessive near work or screen time may increase the risk of myopia or myopia progression, the exact duration or threshold at which this occurs is not well defined. Additionally, other factors such as outdoor time and genetics may also play a role in the development and progression of myopia.  Rather than focusing on a specific duration of screen time or near work, it is recommended to encourage healthy habits such as taking regular breaks from screen time, practicing good posture, and maintaining proper lighting when doing close work. Additionally, spending time outdoors, particularly in natural light, has been suggested as a potential way to reduce the risk of myopia or myopia progression.  It is important to consult with an optometrist or ophthalmologist who specializes in pediatric eye care to determine the most appropriate treatment plan for your child based on their specific situation. |
| 1. **How much outdoor time does my child require to prevent myopia/ myopia progression?** | The amount of outdoor time required to prevent myopia or myopia progression is not well defined, and there is no specific guideline or recommendation on how much outdoor time is needed. However, some studies suggest that spending at least 2 hours per day outdoors, particularly in natural light, may be beneficial in reducing the risk of myopia or slowing down its progression in children.  The exact mechanism by which outdoor time may help prevent myopia is not fully understood, but it is thought to be related to the increased levels of natural light, exposure to different visual stimuli, and the relaxation of the eye muscles that occurs when looking at distant objects.  It is important to note that outdoor time alone may not be enough to prevent myopia, and other factors such as genetics and screen time also play a role in the development and progression of myopia. Additionally, the optimal duration and frequency of outdoor time may vary depending on individual factors such as age, ethnicity, and geographic location.  It is recommended to consult with an optometrist or ophthalmologist who specializes in pediatric eye care to determine the most appropriate treatment plan for your child based on their specific situation. |
| 1. **What type of diet/ vitamin supplementation can help to prevent myopia/ myopia progression?** | There is some evidence to suggest that certain nutrients and dietary factors may play a role in preventing myopia or slowing its progression, but more research is needed to fully understand the relationship between diet and myopia. Some of the nutrients that have been studied in relation to myopia include:   1. **Vitamin D**: Low levels of vitamin D have been associated with an increased risk of myopia, and some studies suggest that supplementation with vitamin D may help prevent myopia or slow its progression. 2. **Omega-3 fatty acids**: Omega-3 fatty acids, particularly DHA and EPA, have been shown to have anti-inflammatory properties and may help reduce the risk of myopia or slow its progression. 3. **Lutein and zeaxanthin**: These are carotenoids found in leafy green vegetables and other foods, and have been suggested to have a protective effect against myopia. 4. **Zinc**: Zinc is an essential mineral that is involved in many cellular processes, and has been shown to play a role in regulating eye growth and development. 5. **Antioxidants**: Antioxidants such as vitamin C and vitamin E may help protect against oxidative stress in the eye, which is thought to contribute to the development and progression of myopia.   It is important to note that while these nutrients may have potential benefits in preventing myopia, they should not be relied upon as a sole means of prevention. It is always best to obtain nutrients from a balanced and varied diet that includes a wide range of fruits, vegetables, whole grains, lean proteins, and healthy fats. If you are considering vitamin supplementation for your child, it is recommended to consult with a healthcare provider first to determine if it is appropriate and to discuss the appropriate dosage. |
| 1. **Are there any available vision therapy/ eye exercises that can help to prevent myopia/ myopia progression?** | There is currently no strong evidence to suggest that vision therapy or eye exercises can effectively prevent myopia or slow its progression. While some studies have investigated the use of vision therapy and eye exercises in managing myopia, the results have been mixed and there is not enough evidence to support their widespread use for myopia prevention.  However, there are some eye exercises that may be helpful in reducing eye strain and fatigue associated with prolonged near work, which can be a contributing factor to myopia progression. These exercises include:   1. **The 20-20-20 rule**: Every 20 minutes, look away from your screen or book and focus on an object at least 20 feet away for at least 20 seconds. 2. **Blinking exercises**: Regularly blinking your eyes helps to lubricate them and reduce dryness and strain. 3. **Near-far focusing**: Alternate focusing on a nearby object and then on a faraway object to exercise the eye muscles and reduce strain.   While these exercises may be helpful in reducing eye strain, they should not be relied upon as a sole means of preventing myopia or slowing its progression. It is still important to follow other recommended strategies such as taking frequent breaks, maintaining proper posture, and increasing outdoor time to reduce the risk of myopia. |
| 1. **What medications are available to prevent myopia/ myopia progression?** | At the moment, there are no medications specifically approved for the prevention or treatment of myopia. Some medications, such as atropine eye drops, have been used off-label to slow the progression of myopia, but their long-term safety and efficacy in children is still being studied.  Atropine eye drops work by dilating the pupil and temporarily paralyzing the focusing mechanism of the eye, which may help to slow down myopia progression. Low-dose atropine eye drops (0.01%) have been found to be effective in slowing myopia progression in children, with minimal side effects. However, the use of atropine eye drops for myopia control is still relatively new, and more research is needed to fully understand its long-term effects and optimal dosages.  It is important to note that the use of any medication for myopia control should be done under the supervision of an eye care professional. |
| 1. **Is atropine safe in children?** | Low-dose atropine eye drops (0.01%) have been found to be safe and effective for myopia control in children. At this dosage, atropine is well-tolerated and has minimal side effects, which may include temporary blurred vision, increased sensitivity to light, and difficulty with near vision.  However, it is important to note that higher doses of atropine can cause more significant side effects such as dilated pupils, dry mouth, and increased heart rate, which may be of concern in young children. Therefore, the use of atropine for myopia control should always be done under the supervision of an eye care professional.  Additionally, the long-term safety of using atropine for myopia control is still being studied. While low-dose atropine has been found to be effective in slowing myopia progression, it is still not clear whether it has any long-term effects on the eye or overall health. |
| 1. **What are the side effects of atropine?** | Low-dose atropine eye drops (0.01%) used for myopia control have minimal side effects, which may include temporary blurred vision, increased sensitivity to light, and difficulty with near vision. These side effects are generally mild and temporary, and typically resolve after a few weeks of use.  However, higher doses of atropine can cause more significant side effects such as dilated pupils, dry mouth, constipation, urinary retention, and increased heart rate. These side effects may be of concern in young children, especially at doses higher than 0.01%.  It is important to note that the risk of side effects is generally low when atropine is used as prescribed under the supervision of an eye care professional. If you have any concerns about the use of atropine for myopia control, you should discuss them with your child's eye doctor. |
| 1. **How effective is atropine at helping to prevent myopia/ myopia progression?** | Atropine eye drops have been found to be effective in slowing the progression of myopia in children. Studies have shown that using low-dose atropine eye drops (0.01%) can reduce myopia progression by up to 50% over two years compared to a placebo.  Atropine works by relaxing the focusing mechanism of the eye, which reduces the strain on the eye muscles and may help to slow down the growth of the eyeball. However, the exact mechanism of how atropine works for myopia control is still not fully understood.  It is important to note that while atropine is effective in slowing myopia progression, it does not cure myopia or reverse the damage that has already been done. Atropine eye drops are typically used in combination with other myopia control methods, such as orthokeratology lenses or multifocal contact lenses, to provide the best possible outcome for myopia control. |
| 1. **What concentration of atropine is available, and which is the most effective?** | There are different concentrations of atropine eye drops that have been used for myopia control. The most common concentrations used are 0.01%, 0.025%, and 0.05%.  Research studies have found that low-dose atropine eye drops (0.01%) are effective in slowing the progression of myopia while having minimal side effects. In contrast, higher concentrations of atropine (0.025% and 0.05%) have been found to be more effective in reducing myopia progression, but they may also have more side effects such as photophobia (sensitivity to light), glare, and difficulty with near vision.  Therefore, most eye care professionals prefer to use low-dose atropine eye drops (0.01%) for myopia control as they are effective in slowing the progression of myopia while minimizing side effects. |
| 1. **How does atropine help to prevent myopia/ myopia progression?** | The exact mechanism of how atropine works for myopia control is not fully understood, but it is believed to work by relaxing the focusing mechanism of the eye, which reduces the strain on the eye muscles and may help to slow down the growth of the eyeball.  In people with myopia, the eyeball grows too long, causing light to focus in front of the retina instead of on it, resulting in blurry vision. Atropine eye drops work by temporarily paralyzing the focusing mechanism of the eye, which reduces the demand for the eye muscles to focus, allowing the eye to relax and reducing the strain on the eye muscles.  Atropine eye drops also affect the part of the eye responsible for regulating eye growth, called the retina pigment epithelium (RPE). Atropine eye drops are believed to alter the signals that the RPE sends to the eye, which can help to slow down the growth of the eyeball and prevent myopia progression.  It's important to note that while atropine is effective in slowing myopia progression, it does not cure myopia or reverse the damage that has already been done. Atropine eye drops are typically used in combination with other myopia control methods, such as orthokeratology lenses or multifocal contact lenses, to provide the best possible outcome for myopia control. |
| 1. **My child has not developed myopia, should he/ she start using atropine?** | No, your child should not start using atropine eye drops unless prescribed by an eye doctor for myopia management. Atropine eye drops are a medication used to slow down the progression of myopia in children who have already developed myopia. They are not recommended for children who do not have myopia or for those with mild myopia.  If your child has not developed myopia, there are other preventative measures that can be taken to reduce the risk of myopia development, such as spending time outdoors, reducing screen time, and taking frequent breaks during near work activities. It's also important for your child to have regular eye exams to monitor for any changes in vision and to ensure that any potential vision problems are detected and treated early. |
| 1. **My child is on atropine for myopia control, when can it be stopped?** | The duration of atropine treatment for myopia control depends on various factors such as the age of the child, the severity of myopia, and the rate of myopia progression. Generally, atropine treatment is continued until the child's myopia stabilizes, which usually occurs in the late teenage years.  Your child's eye doctor will regularly monitor your child's myopia progression and the effectiveness of the atropine treatment. If the myopia progression slows down and stabilizes, the doctor may recommend reducing the dosage of atropine or stopping the treatment altogether. However, it is important to follow the doctor's instructions and not to stop the treatment on your own without consulting the doctor.  If you have any concerns about your child's myopia treatment, you should consult your eye doctor. |
| **Topic: Prognosis** | |
| 1. **Is myopia reversible?** | Myopia is not generally considered reversible, as the elongation of the eye that causes myopia is a permanent change. However, the progression of myopia can sometimes be slowed or even stopped with appropriate treatment, which can help prevent further worsening of the condition.  In some cases, myopia may temporarily improve with the use of certain treatments, such as orthokeratology lenses or atropine eye drops, but this improvement is usually not permanent and requires ongoing treatment to maintain.  It's important to note that early detection and management of myopia can help prevent complications and reduce the risk of severe myopia later in life. |
| 1. **Why is myopia a concern? What are the potential complications of myopia?** | Myopia, or nearsightedness, is a concern because it can significantly affect a person's vision and quality of life. Some potential complications of myopia include:   1. **Reduced vision**: Myopia can cause blurry vision when looking at distant objects, which can affect a person's ability to drive, play sports, or perform other activities that require good distance vision. 2. **Strain on the eyes**: People with myopia may need to squint or strain to see distant objects, which can cause eye fatigue, headaches, and neck and shoulder pain. 3. **Higher risk of eye diseases**: People with myopia are at a higher risk of developing other eye conditions, such as cataracts, glaucoma, and retinal detachment, which can lead to vision loss or blindness. 4. **Social and emotional effects**: Children with myopia may struggle in school or with social activities due to difficulty seeing the board or recognizing faces at a distance, which can affect their self-esteem and quality of life. 5. **Increased risk of severe myopia**: High levels of myopia, or severe myopia, can increase the risk of vision-threatening complications such as retinal detachment, myopic maculopathy, and glaucoma.   It's important to detect and manage myopia early to reduce the risk of complications and ensure good vision and quality of life. |
| 1. **Can myopia cause blindness?** | In most cases, myopia by itself does not cause blindness. However, severe myopia (high myopia) can increase the risk of certain eye conditions that can potentially cause vision loss, such as retinal detachment, myopic maculopathy, and glaucoma. These conditions occur when the eyeball elongates excessively, causing stretching and thinning of the retina, which is the layer of cells that senses light at the back of the eye. Over time, this stretching and thinning can lead to damage to the retina, optic nerve, and other structures of the eye, resulting in vision loss or even blindness if left untreated. Therefore, it is important to manage myopia properly and monitor for any potential complications. |

# Supplementary Table 1b: ChatGPT-4.0 Responses to Myopia-Related Questions

| **Question** | **ChatGPT-4.0 Response** |
| --- | --- |
| **Topic: Pathogenesis** | |
| 1. **What is myopia?** | Myopia, also known as nearsightedness or shortsightedness, is a common refractive error in the eye. It occurs when the shape of the eye causes light rays to focus incorrectly, resulting in distant objects appearing blurry while near objects remain clear.  In a myopic eye, the eyeball is usually elongated, or the cornea (the clear front surface of the eye) is too curved. As a result, light entering the eye focuses in front of the retina (the light-sensitive tissue at the back of the eye) rather than directly on it. This causes distant objects to appear out of focus.  Myopia can develop gradually or rapidly, often worsening during childhood and adolescence. It typically stabilizes in early adulthood but can continue to progress in some individuals. Myopia is a global public health concern, as its prevalence has been increasing over the past few decades, particularly in urbanized regions. |
| 1. **At what age does myopia commonly develop?** | Myopia commonly develops during childhood and adolescence. It often starts between the ages of 6 and 14, with the most rapid progression usually occurring between the ages of 8 and 12. The progression of myopia tends to slow down as a child gets older, and it typically stabilizes in early adulthood. However, the age of onset and the rate of progression can vary significantly among individuals.  It is essential to monitor children's vision regularly, as early detection of myopia can help initiate appropriate interventions to manage its progression and reduce the risk of associated complications. |
| 1. **What causes myopia?** | Myopia is caused by a combination of genetic and environmental factors. The main factors contributing to the development and progression of myopia are:   1. **Genetics**: A family history of myopia increases the likelihood of a person developing the condition. If one or both parents are myopic, their child is at a higher risk of developing myopia. 2. **Environmental factors**: Increased near-work activities, such as reading, writing, and using digital devices for extended periods, have been associated with a higher risk of developing myopia. Additionally, spending less time outdoors and engaging in fewer outdoor activities has been linked to a higher prevalence of myopia in children. 3. **Eye structure**: Myopia occurs when the eyeball is elongated or when the cornea is too curved. These structural abnormalities cause light to focus in front of the retina instead of directly on it, resulting in blurry vision for distant objects.   It's important to note that the specific cause of myopia can vary among individuals, and the interaction between genetic and environmental factors is complex. Ongoing research aims to further elucidate the underlying mechanisms contributing to the development and progression of myopia. |
| **Topic: Risk Factors** | |
| 1. **What puts my child at risk of myopia?** | Several factors can put a child at risk of developing myopia. These risk factors include:   1. **Genetics**: If one or both parents have myopia, the child is at a higher risk of developing the condition, as there is a genetic predisposition for myopia. 2. **Prolonged near work**: Spending excessive time on activities that require focusing on close objects, such as reading, writing, or using digital devices (smartphones, tablets, computers), can increase the risk of myopia development in children. 3. **Insufficient outdoor time**: Studies have shown that children who spend more time outdoors have a lower risk of developing myopia. Exposure to natural sunlight and engaging in outdoor activities might have a protective effect against myopia. 4. **Ethnicity**: Some studies suggest that certain ethnicities, such as individuals of East Asian descent, have a higher prevalence of myopia compared to other populations.   It's essential to monitor children's vision regularly and encourage healthy habits, such as taking breaks from near work, engaging in outdoor activities, and maintaining a balanced lifestyle, to minimize the risk of myopia development and progression. |
| 1. **Is myopia hereditary? My spouse and I have myopia, how likely is it for my child to get myopia?** | Myopia has a hereditary component, meaning that it can be passed down from parents to their children through genes. If both you and your spouse have myopia, your child has a higher risk of developing the condition compared to a child with no family history of myopia.  While it's difficult to provide an exact likelihood, studies have shown that children with one or both parents who are myopic are at an increased risk. However, genetics is only one factor influencing myopia development; environmental factors, such as prolonged near work and lack of outdoor exposure, also play a role.  To minimize the risk of myopia development and progression in your child, it's essential to encourage healthy habits like regular breaks from near work, spending time outdoors, and maintaining a balanced lifestyle. Additionally, regular eye checkups can help detect the early onset of myopia, allowing for timely intervention and management. |
| **Topic: Clinical Presentation** | |
| 1. **What are signs and symptoms that suggests my child has myopia?** | Some signs and symptoms that may suggest your child has myopia include:   1. **Squinting**: Children with myopia may squint when trying to see distant objects more clearly, as squinting can temporarily improve focus. 2. **Sitting close to screens or the front of the classroom**: Myopic children might have difficulty seeing distant objects and may prefer to sit closer to screens or the front of the classroom to see more clearly. 3. **Frequent blinking or eye rubbing**: These behaviors may indicate eye strain or discomfort, which can be caused by uncorrected vision problems like myopia. 4. **Headaches**: Frequent headaches can be a sign of eye strain, which may result from uncorrected vision issues, including myopia. 5. **Difficulty seeing distant objects**: Children with myopia might struggle to see objects or read signs at a distance, even if they can see things up close without any issues.   If you notice any of these signs in your child, it is essential to consult an eye care professional for a comprehensive eye exam. Early detection of myopia allows for timely intervention and management, which can help reduce the risk of complications associated with high myopia later in life. |
| **Topic: Diagnosis** | |
| 1. **How is myopia diagnosed?** | Myopia is diagnosed through a comprehensive eye examination conducted by an eye care professional, such as an optometrist or ophthalmologist. The eye exam typically consists of several tests and assessments to evaluate the overall health of the eyes and determine the presence of any refractive errors, including myopia. Some common tests used to diagnose myopia include:   1. **Visual acuity test**: This test measures the sharpness of vision using a standardized eye chart with rows of letters, numbers, or symbols that decrease in size. The patient is asked to read the chart from a specific distance to determine the clarity of their distance vision. 2. **Retinoscopy**: The eye care professional shines a light into the patient's eyes and observes the reflection off the retina. By moving a series of lenses in front of the eyes, the practitioner can estimate the refractive error and determine the appropriate corrective lenses needed. 3. **Refraction assessment**: This test fine-tunes the prescription for corrective lenses. The patient looks through a device called a phoropter, which holds various lenses in front of the eyes. The eye care professional will switch between lenses and ask the patient to compare which lens provides clearer vision. 4. **Eye health examination**: The eye care professional may also assess the overall health of the eyes, including the structures within the eye, to rule out any other eye conditions that could affect vision.   If myopia is diagnosed, the eye care professional will provide a prescription for corrective lenses (glasses or contact lenses) to help the patient see distant objects more clearly. Early diagnosis and intervention are crucial for managing myopia progression and minimizing the risk of complications associated with high myopia later in life. |
| 1. **What should I do if I suspect my child has myopia?** | If you suspect your child has myopia or is experiencing vision problems, it's essential to take the following steps:   1. **Schedule an eye examination**: Make an appointment with an eye care professional, such as an optometrist or ophthalmologist, for a comprehensive eye exam. The eye care professional will evaluate your child's vision and eye health, diagnose any refractive errors, including myopia, and provide appropriate recommendations. 2. **Share your observations**: During the appointment, discuss the signs and symptoms you have noticed in your child with the eye care professional. This information will help them better understand your child's vision concerns and provide more accurate guidance. 3. **Follow the recommendations**: If your child is diagnosed with myopia or another vision problem, follow the eye care professional's recommendations regarding corrective lenses (glasses or contact lenses), vision therapy, or any other interventions they may suggest. 4. **Monitor your child's vision**: Keep an eye on your child's vision and note any changes or concerns. Regular eye checkups are essential to track myopia progression and adjust the prescription as needed. 5. **Encourage healthy habits**: Teach your child the importance of taking breaks from near work, spending time outdoors, and maintaining a balanced lifestyle. These habits can help minimize the risk of myopia progression and promote overall eye health.   By taking these steps, you can ensure early detection and management of myopia, helping to reduce the risk of complications associated with high myopia later in life. |
| **Topic: Treatment and Prevention** | |
| 1. **What can I do to prevent myopia?** | While it may not be possible to completely prevent myopia, especially in cases where genetics play a significant role, there are steps you can take to reduce the risk of myopia development or slow down its progression:   1. **Spend time outdoors**: Encourage outdoor activities and ensure children have ample exposure to natural sunlight. Studies have shown that spending more time outdoors is associated with a reduced risk of developing myopia. 2. **Take breaks from near work**: Prolonged near work, such as reading, writing, or using digital devices, can contribute to myopia development. Make sure to take regular breaks when doing near work, following the 20-20-20 rule: every 20 minutes, take a 20-second break and look at something 20 feet away. 3. **Maintain a balanced lifestyle**: Encourage a well-rounded lifestyle, including a mix of physical activities, outdoor play, and social interactions, to minimize the risk of myopia development. 4. **Proper ergonomics**: Ensure proper posture and distance when using digital devices or doing other near work tasks. Maintain a comfortable reading distance and avoid holding devices too close to the eyes. 5. **Regular eye checkups**: Regular eye exams can help detect myopia and other vision problems early on, allowing for timely intervention and management.   It's essential to understand that while these strategies can help reduce the risk of myopia development or slow its progression, they may not be completely effective in preventing myopia, particularly in individuals with a strong genetic predisposition. However, following these recommendations can still promote overall eye health and well-being. |
| 1. **My child has myopia, what are the available treatments to slow down the progression of myopia?** | There are several treatment options available to slow down the progression of myopia in children. These treatments, also known as myopia control strategies, can help reduce the risk of developing high myopia and its associated complications later in life. Some common myopia control methods include:   1. **Orthokeratology (Ortho-K)**: Ortho-K involves wearing specially designed rigid gas permeable contact lenses overnight, which temporarily reshape the cornea to correct myopia. The lenses are removed during the day, allowing for clear vision without the need for glasses or contact lenses. Ortho-K has been shown to slow down myopia progression in some children. 2. **Multifocal contact lenses**: These contact lenses have multiple prescriptions within a single lens, providing clear vision at different distances. Studies have shown that multifocal contact lenses can help slow down myopia progression in children by reducing peripheral hyperopic defocus. 3. **Multifocal eyeglasses**: Similar to multifocal contact lenses, multifocal eyeglasses have multiple prescriptions in a single lens. While they may be less effective than multifocal contact lenses or Ortho-K, they can still provide some myopia control benefits. 4. **Atropine eye drops**: Low-dose atropine eye drops have been shown to slow down myopia progression in children. Atropine is thought to work by relaxing the eye's focusing mechanism, reducing strain on the eyes during near work. 5. **Behavioral changes**: Encouraging outdoor activities, taking breaks from near work, and maintaining a balanced lifestyle can also help slow down myopia progression.   It's important to consult an eye care professional to determine the most suitable myopia control strategy for your child. They will evaluate your child's specific needs, monitor their vision, and adjust the treatment plan as necessary. |
| 1. **How can I prevent myopia/ myopia progression without the use of medications?** | While it may not be possible to completely prevent myopia, especially when genetics play a significant role, you can take some steps to reduce the risk of myopia development or slow down its progression without using medications:   1. **Spend time outdoors**: Encourage outdoor activities and ensure children have ample exposure to natural sunlight. Studies have shown that spending more time outdoors is associated with a reduced risk of developing myopia and can help slow down its progression. 2. **Take breaks from near work**: Prolonged near work, such as reading, writing, or using digital devices, can contribute to myopia development. Make sure to take regular breaks when doing near work, following the 20-20-20 rule: every 20 minutes, take a 20-second break and look at something 20 feet away. 3. **Maintain a balanced lifestyle**: Encourage a well-rounded lifestyle, including a mix of physical activities, outdoor play, and social interactions, to minimize the risk of myopia development and progression. 4. **Proper ergonomics**: Ensure proper posture and distance when using digital devices or doing other near work tasks. Maintain a comfortable reading distance and avoid holding devices too close to the eyes. 5. **Regular eye checkups**: Regular eye exams can help detect myopia and other vision problems early on, allowing for timely intervention and management.   Keep in mind that these strategies may not completely prevent myopia or its progression, particularly in individuals with a strong genetic predisposition. However, following these recommendations can still promote overall eye health and well-being. |
| 1. **What are the spectacles/contact lenses available to prevent myopia/ myopia progression?** | Several types of spectacles and contact lenses are specifically designed to help prevent or slow down myopia progression. Some of the options include:   1. **Multifocal eyeglasses**: These glasses have multiple prescriptions within a single lens, providing clear vision at different distances. By addressing peripheral hyperopic defocus, multifocal eyeglasses can help slow down myopia progression, although their effectiveness may be less than other options. 2. **Multifocal contact lenses**: Like multifocal eyeglasses, multifocal contact lenses have multiple prescriptions in a single lens. They have been shown to be more effective in slowing down myopia progression than single-vision lenses by reducing peripheral hyperopic defocus. 3. **Orthokeratology (Ortho-K)**: Ortho-K involves wearing specially designed rigid gas permeable contact lenses overnight, which temporarily reshape the cornea to correct myopia. The lenses are removed during the day, allowing for clear vision without the need for glasses or contact lenses. Ortho-K has been shown to slow down myopia progression in some children. 4. **Soft bifocal or multifocal contact lenses**: Soft bifocal or multifocal contact lenses provide a more comfortable alternative to Ortho-K and may be more suitable for some individuals. These lenses have been shown to be effective in slowing down myopia progression by reducing peripheral hyperopic defocus. 5. **Myopia control spectacle lenses**: Some specially designed spectacle lenses, like Hoya's MiYOSMART lenses or Zeiss's MyoVision lenses, have been developed to slow down myopia progression. These lenses utilize advanced technologies, like Defocus Incorporated Multiple Segments (DIMS) or peripheral defocus management, to reduce myopia progression.   It's important to consult an eye care professional to determine the most suitable myopia control option for you or your child. They will evaluate the specific needs, monitor vision, and adjust the treatment plan as necessary. |
| 1. **How effective is MiSight/ MiyoSmart/ Stellest/ Abiliti/ orthokeratology lenses at helping to prevent myopia/ myopia progression?** | MiSight, MiyoSmart, Stellest, Abiliti, and orthokeratology lenses are all designed to help slow down myopia progression, and they have shown varying degrees of effectiveness in clinical trials and studies. Here's a brief overview of their effectiveness:  **MiSight**: MiSight contact lenses are daily disposable soft multifocal lenses. A 3-year clinical trial demonstrated that MiSight lenses slowed myopia progression by an average of 59% compared to single-vision lenses, with some variation among individual participants.  **MiyoSmart**: MiyoSmart lenses, developed by Hoya, utilize Defocus Incorporated Multiple Segments (DIMS) technology. A 2-year clinical trial found that MiyoSmart lenses slowed myopia progression by an average of 60% in terms of myopic refractive error and 50% in terms of axial elongation, compared to single-vision lenses.  **Stellest**: Essilor's Stellest lenses employ H.A.L.T. (Highly Aspherical Lenslet Target) technology, which provides myopia control by creating multiple focal points on the retina. A 1-year clinical trial showed that Stellest lenses slowed myopia progression by an average of 67% in terms of myopic refractive error and 62% in terms of axial elongation, compared to single-vision lenses.  **Abiliti**: Abiliti Overnight Therapeutic Lenses by CooperVision are orthokeratology lenses designed for overnight wear. While specific data on Abiliti lenses is limited, orthokeratology lenses, in general, have been shown to slow down myopia progression by 30-50% in various studies.  It's essential to understand that individual results may vary, and the effectiveness of these lenses can depend on factors such as the degree of myopia, age, and adherence to the prescribed treatment plan. Consult an eye care professional to determine the most suitable myopia control option for you or your child. They will evaluate specific needs, monitor vision, and adjust the treatment plan as necessary. |
| 1. **How does MiSight/ MiyoSmart/ Stellest/ Abiliti/ orthokeratology lenses help to prevent myopia/ myopia progression?** | MiSight, MiyoSmart, Stellest, Abiliti, and orthokeratology lenses have different mechanisms of action, but they all aim to slow down myopia progression by addressing the peripheral hyperopic defocus, which is believed to contribute to myopia development. Here's how each of them works:  **MiSight**: MiSight lenses are daily disposable soft multifocal contact lenses. They feature concentric rings with alternating distance and near powers. These lenses provide clear central vision while simultaneously creating myopic defocus in the peripheral retina, reducing the stimulus for eye growth and thus slowing down myopia progression.  **MiyoSmart**: MiyoSmart lenses by Hoya utilize Defocus Incorporated Multiple Segments (DIMS) technology. The lens has a central optical zone for clear vision, surrounded by multiple small lenslets with a slightly increased power. These lenslets create myopic defocus in the peripheral retina, counteracting the stimulus for eye growth and thereby slowing myopia progression.  **Stellest**: Essilor's Stellest lenses employ H.A.L.T. (Highly Aspherical Lenslet Target) technology. The lenses create multiple focal points on the retina, with the central zone providing clear vision and the surrounding aspherical lenslets creating myopic defocus in the peripheral retina. This defocus reduces the stimulus for eye growth and slows down myopia progression.  **Abiliti/Orthokeratology**: Abiliti Overnight Therapeutic Lenses by CooperVision and other orthokeratology lenses are rigid gas permeable lenses designed to be worn overnight. They temporarily reshape the cornea, flattening its central area and steepening the mid-peripheral zone. This reshaping results in clear central vision and myopic defocus in the peripheral retina, reducing the stimulus for eye growth and slowing down myopia progression.  It's important to note that individual results may vary, and the effectiveness of these lenses can depend on factors such as the degree of myopia, age, and adherence to the prescribed treatment plan. Consult an eye care professional to determine the most suitable myopia control option for you or your child. They will evaluate specific needs, monitor vision, and adjust the treatment plan as necessary. |
| 1. **Is it safe for my child to be using myopia control contact lens at such a young age?** | Yes, it is generally safe for children to use myopia control contact lenses at a young age. Many studies have shown that these lenses are both safe and effective in slowing down myopia progression in children. However, it is crucial to consult an eye care professional to determine if contact lenses are the right choice for your child.  An eye care professional will assess your child's eye health, maturity, and ability to handle contact lenses properly. They will also provide guidance on hygiene and lens care to minimize the risk of eye infections or complications. Parental supervision and support in lens care routines are essential to ensure that children adhere to the recommended guidelines.  Your child's eye care professional will closely monitor their eye health, vision, and contact lens use, making any necessary adjustments to the prescription or treatment plan. If contact lenses are not suitable for your child, alternative myopia control options, such as specially designed spectacle lenses, may be recommended. |
| 1. **How effective is reducing screen time/ increasing outdoor time at helping to prevent myopia/ myopia progression?** | Increasing outdoor time and reducing screen time have been associated with a lower risk of developing myopia and slowing down its progression. Research suggests that spending more time outdoors can have a protective effect against myopia development in children.  A meta-analysis of multiple studies found that for each additional hour spent outdoors per week, the risk of myopia decreased by approximately 2%. The exact mechanism behind the protective effect of outdoor time is not yet fully understood, but it may be related to increased exposure to natural sunlight, which can stimulate the release of dopamine in the retina, reducing eye growth and slowing down myopia progression.  On the other hand, excessive screen time and near work activities have been linked to a higher risk of developing myopia. Close-up tasks such as reading, writing, or using electronic devices may contribute to eye strain and increased eye growth, leading to myopia development or progression. Encouraging children to take regular breaks from near work activities and maintaining a balanced lifestyle with a mix of outdoor activities and screen time can help reduce the risk of myopia.  While increasing outdoor time and reducing screen time can be beneficial, it's important to note that these factors alone may not entirely prevent myopia or its progression. A comprehensive approach that considers genetics, lifestyle factors, and appropriate myopia management strategies under the guidance of an eye care professional is essential for optimal outcomes. |
| 1. **What is the maximum duration of screen time/ near work per day before my child is at risk of myopia/ myopia progression?** | There is no specific "maximum duration" of screen time or near work per day that applies to all children, as individual risk factors for myopia and myopia progression can vary. However, it is generally recommended to practice moderation and balance when it comes to screen time and near work activities.  The American Academy of Pediatrics (AAP) recommends the following guidelines for screen time, although these guidelines primarily focus on overall child development rather than specifically addressing myopia risk:   1. **Children under 18 months**: No screen time, except for video chatting 2. **Children aged 18 to 24 months**: Limited and supervised high-quality programming 3. **Children aged 2 to 5 years**: No more than one hour per day of high-quality programming 4. **Children aged 6 years and older**: Consistent limits on screen time and ensuring that it doesn't interfere with sleep, physical activity, and other essential aspects of a child's life   To minimize the risk of myopia and myopia progression, it is essential to:   1. **Encourage regular breaks from near work activities**: The 20-20-20 rule is a helpful guideline, suggesting that every 20 minutes, take a 20-second break to look at something 20 feet (6 meters) away. 2. **Ensure proper ergonomics** when using screens or engaging in near work, such as maintaining an appropriate distance from the screen (around 40-60 cm or 16-24 inches) and using good lighting. 3. **Promote a balanced lifestyle** that includes outdoor activities, physical exercise, and sufficient sleep.   It is important to consult an eye care professional to discuss your child's specific needs and receive personalized advice on myopia prevention and management strategies. |
| 1. **How much outdoor time does my child require to prevent myopia/ myopia progression?** | There is no specific amount of outdoor time that can guarantee the prevention of myopia or myopia progression in every child, as individual factors can vary. However, research suggests that spending more time outdoors is associated with a lower risk of developing myopia and may help slow down its progression.  A general recommendation, based on multiple studies, is to encourage children to spend at least 90 minutes to 2 hours per day outdoors. This can help reduce the risk of myopia onset and may slow down its progression. The protective effect of outdoor time is thought to be related to increased exposure to natural sunlight, which can stimulate the release of dopamine in the retina, reducing eye growth and the progression of myopia.  It is essential to remember that while increasing outdoor time can be beneficial, it may not entirely prevent myopia or its progression on its own. A comprehensive approach that considers genetic factors, lifestyle choices, and appropriate myopia management strategies under the guidance of an eye care professional is necessary for optimal outcomes. |
| 1. **What type of diet/ vitamin supplementation can help to prevent myopia/ myopia progression?** | While the relationship between diet and myopia is not yet fully understood, some studies have suggested that certain dietary factors and vitamin supplements may have a potential role in preventing myopia or slowing down its progression.  1. **Omega-3 fatty acids**: These essential fatty acids, found in fish, nuts, and seeds, are known to support eye health. Some studies suggest that a higher intake of omega-3 fatty acids may be associated with a lower risk of myopia development.  2. **Antioxidants and vitamins**: Vitamins A, C, and E, along with antioxidants like lutein and zeaxanthin, have been associated with better eye health. These nutrients can be found in various fruits and vegetables, such as leafy greens, carrots, and bell peppers.  3. **Vitamin D**: Some studies have suggested a potential link between vitamin D deficiency and a higher risk of myopia. Sun exposure, fortified foods, and supplements are sources of vitamin D.  However, the evidence linking specific dietary factors or supplements with myopia prevention or management is still limited and inconclusive. It is crucial to consult a healthcare professional before starting any vitamin supplementation or making significant dietary changes.  Maintaining a balanced, nutrient-rich diet that supports overall health and eye health is essential. Encouraging a healthy lifestyle with a mix of outdoor activities, regular eye exams, and consultation with an eye care professional can help ensure a comprehensive approach to myopia prevention and management. |
| 1. **Are there any available vision therapy/ eye exercises that can help to prevent myopia/ myopia progression?** | There is limited and inconclusive evidence to support the effectiveness of vision therapy or eye exercises in preventing myopia or slowing down its progression. Some eye exercises and vision therapies may help improve visual skills or alleviate eye strain, but their impact on myopia control is not well-established.  Currently, the most effective methods for myopia control are:   1. **Prescription eyewear**: Specialized spectacle lenses, such as bifocal or progressive lenses, designed for myopia control. 2. **Contact lenses**: Myopia control contact lenses, like orthokeratology lenses or soft multifocal lenses, have been shown to slow down myopia progression. 3. **Pharmacological intervention**: Atropine eye drops, in low concentrations, have been proven effective in slowing down myopia progression in children.   In addition to these interventions, promoting a balanced lifestyle with outdoor activities, limiting excessive near work or screen time, and maintaining good eye care practices can help reduce the risk of myopia onset or progression.  It is crucial to consult an eye care professional for personalized recommendations and a comprehensive approach to myopia prevention and management. |
| 1. **What medications are available to prevent myopia/ myopia progression?** | Atropine eye drops are currently the most widely researched and used medication for preventing myopia progression. Atropine is a muscarinic antagonist that has been shown to slow down myopia progression in children when used in low concentrations, typically between 0.01% and 0.05%.  The exact mechanism by which atropine slows myopia progression is not yet fully understood. However, it is believed to affect the biochemical processes in the eye, which influence eye growth and elongation, thus slowing down the progression of myopia.  It is essential to consult an eye care professional before starting any medication for myopia control. They will assess your child's specific needs, consider potential side effects, and determine the most appropriate treatment plan. In addition to pharmacological interventions, other myopia control strategies, such as specialized prescription eyewear or contact lenses, should also be considered. |
| 1. **Is atropine safe in children?** | Atropine eye drops, when used in low concentrations (typically between 0.01% and 0.05%), have been shown to be generally safe and well-tolerated in children for myopia control. Numerous studies have demonstrated the effectiveness of low-dose atropine in slowing down myopia progression with minimal side effects.  However, atropine at higher concentrations can cause side effects, such as increased sensitivity to light (photophobia), blurred near vision, and dilation of the pupils (mydriasis), which could lead to difficulty focusing on near objects. These side effects are typically less common and less severe with lower concentrations of atropine.  It is important to consult an eye care professional before starting atropine treatment for myopia control in children. The eye care professional will carefully assess the child's individual needs, discuss potential side effects, and determine the most appropriate treatment plan.  If your child is prescribed atropine eye drops, it is essential to follow the eye care professional's instructions regarding dosage and frequency, and to attend regular follow-up appointments to monitor the child's eye health and response to treatment. |
| 1. **What are the side effects of atropine?** | Atropine eye drops, when used for myopia control, can cause some side effects. These side effects are typically more common and more severe with higher concentrations of atropine, but they tend to be milder and less frequent when using low concentrations (0.01% to 0.05%). Some possible side effects of atropine include:  1. **Pupil dilation (mydriasis)**: Atropine can cause the pupils to dilate, which may lead to difficulty focusing on near objects and a temporary decrease in near vision.  2. **Light sensitivity (photophobia)**: Dilated pupils may let in more light, causing increased sensitivity to bright lights and glare. This can be managed by wearing sunglasses or photochromic lenses when outdoors.  3. **Blurred near vision**: Atropine can affect the eye's ability to focus on near objects, leading to temporary blurry vision for close-up tasks.  4. **Eye irritation or redness**: Some people may experience mild eye irritation, redness, or discomfort after using atropine eye drops.  It is essential to consult an eye care professional before starting atropine treatment for myopia control. They will carefully assess the individual's needs, discuss potential side effects, and determine the most appropriate treatment plan.  If side effects occur, it is important to communicate with the eye care professional, as they may adjust the dosage or recommend alternative myopia control strategies. |
| 1. **How effective is atropine at helping to prevent myopia/ myopia progression?** | Atropine eye drops have been shown to be effective in slowing down the progression of myopia in children. Numerous studies, including the Atropine for the Treatment of Myopia (ATOM) and Low-concentration Atropine for Myopia Progression (LAMP) trials, have demonstrated the effectiveness of low-dose atropine (0.01% to 0.05%) in reducing myopia progression.  The degree of effectiveness can vary depending on the concentration of atropine used, the individual response to treatment, and other factors. In general, low-dose atropine has been found to reduce the progression of myopia by approximately 50% to 60% compared to no treatment.  It is essential to consult an eye care professional to determine the most appropriate treatment plan for a child with myopia. The eye care professional will carefully assess the child's individual needs, discuss potential side effects, and consider other myopia control strategies, such as specialized prescription eyewear or contact lenses, in addition to atropine treatment.  Regular follow-up appointments with the eye care professional are crucial to monitor the child's eye health, myopia progression, and response to treatment. |
| 1. **What concentration of atropine is available, and which is the most effective?** | Atropine eye drops come in various concentrations, ranging from 0.01% to 1%. For myopia control, low-concentration atropine has been found to be effective while minimizing side effects.  The most commonly used concentrations for myopia control are:   1. **0.01% atropine**: This concentration has been shown to be effective in slowing down myopia progression with minimal side effects. In the Atropine for the Treatment of Myopia (ATOM) 2 study, 0.01% atropine reduced myopia progression by about 50% compared to a placebo. 2. **0.025% and 0.05% atropine**: The Low-concentration Atropine for Myopia Progression (LAMP) study found that both 0.025% and 0.05% atropine concentrations were more effective in slowing down myopia progression than 0.01% atropine, with only slightly increased side effects. 3. **Higher concentrations of atropine**, such as 0.5% or 1%, have also been studied for myopia control. However, these higher concentrations are associated with a higher risk of side effects, such as photophobia, pupil dilation, and blurred near vision, and are generally not recommended for myopia control.   The most effective concentration of atropine for an individual child depends on their specific needs and the balance between effectiveness and side effects. An eye care professional will assess the child's needs and recommend the most suitable concentration of atropine, taking into account factors such as the child's age, the degree of myopia, and the rate of progression. |
| 1. **How does atropine help to prevent myopia/ myopia progression?** | Atropine eye drops help to slow down the progression of myopia, but the exact mechanism of how it works is not yet fully understood. There are several theories about how atropine might exert its effects on myopia progression:  Relaxation of the ciliary muscle: Atropine is an anticholinergic agent that blocks the action of acetylcholine, leading to the relaxation of the ciliary muscle in the eye. This relaxation might reduce accommodative stress and strain on the eye, which could slow down the elongation of the eyeball and, subsequently, the progression of myopia.  Changes in scleral remodeling: Atropine is thought to influence the scleral remodeling process by affecting the release of certain neurotransmitters and growth factors. By altering the biochemistry of the sclera (the white outer layer of the eye), atropine might help prevent excessive elongation of the eye, which is associated with myopia progression.  Modulation of retinal signaling: Some researchers propose that atropine may have a direct effect on the retina, modulating the signaling pathways that regulate eye growth and development.  It is important to note that atropine does not "cure" myopia or eliminate the need for corrective lenses. Instead, it helps to slow down the progression of myopia, reducing the risk of high myopia and associated complications. Regular eye exams and follow-up appointments with an eye care professional are essential to monitor myopia progression and adjust treatment strategies as needed. |
| 1. **My child has not developed myopia, should he/ she start using atropine?** | Atropine eye drops are typically used for slowing down the progression of myopia in children who have already developed the condition. If your child has not developed myopia, it is generally not recommended to start using atropine as a preventive measure.  Instead, you should focus on preventive strategies that promote eye health and reduce the risk of developing myopia, such as:   1. **Encouraging outdoor activities**: Spending more time outdoors has been associated with a reduced risk of myopia development. Aim for at least 1-2 hours of outdoor time per day. 2. **Taking breaks from near work and screen time**: Encourage your child to follow the 20-20-20 rule – every 20 minutes, take a 20-second break and look at something 20 feet away. This can help reduce eye strain associated with near work. 3. **Ensuring proper lighting**: Good lighting is essential for reducing eye strain when your child is reading or engaging in near work activities. 4. **Monitoring your child's vision**: Regular eye examinations are important for early detection of any vision problems, including myopia. Make sure your child receives an eye exam at the recommended intervals or if you notice any signs of vision difficulties.   If you have concerns about your child's risk of developing myopia, consult an eye care professional for personalized advice and recommendations. They can assess your child's vision and provide guidance on preventive measures and early intervention strategies if necessary. |
| 1. **My child is on atropine for myopia control, when can it be stopped?** | The decision to stop atropine treatment for myopia control in your child should be made in consultation with their eye care professional. The duration of atropine treatment can vary depending on several factors, such as the severity of myopia, the rate of progression, the age of the child, and the effectiveness of the treatment.  Typically, atropine is prescribed for a period of time, and then the child's eye care professional will assess the progression of myopia and the effectiveness of the treatment. If the progression has slowed down significantly, the eye care professional may consider reducing the dosage, changing the treatment plan, or stopping the atropine treatment altogether.  It is important to continue regular eye examinations and follow-up appointments to monitor the progression of myopia and make adjustments to the treatment plan as necessary. If myopia progression resumes after stopping atropine treatment, the eye care professional may recommend resuming or modifying the treatment.  Always consult your child's eye care professional before making any changes to their myopia management plan. They can provide personalized advice based on your child's specific needs and circumstances. |
| **Topic: Prognosis** | |
| 1. **Is myopia reversible?** | Myopia, also known as nearsightedness, is generally not considered reversible in the sense that it can be completely cured or eliminated. Myopia occurs when the eyeball is elongated or the cornea is too curved, causing light entering the eye to focus in front of the retina instead of directly on it. This results in blurred vision for distant objects.  Although myopia cannot be reversed, there are several ways to correct or manage the condition:   1. **Eyeglasses or contact lenses**: Corrective lenses are the most common method for managing myopia. They work by refocusing light onto the retina, providing clearer vision for distant objects. 2. **Orthokeratology (Ortho-K)**: Ortho-K involves wearing specially designed gas-permeable contact lenses overnight to temporarily reshape the cornea, which can improve vision during the day. However, this treatment does not provide a permanent solution, and myopia may return if Ortho-K lenses are discontinued. 3. **Refractive surgery**: Procedures like LASIK and PRK can reshape the cornea, permanently improving vision in some cases. However, refractive surgery is not suitable for everyone and carries certain risks, so it's essential to consult an eye care professional to determine if it's the right option for you. 4. **Myopia control**: While it doesn't reverse myopia, myopia control aims to slow down its progression, especially in children. Treatments like atropine eye drops, multifocal contact lenses, or Ortho-K lenses can be effective in slowing the progression of myopia, reducing the risk of complications associated with high myopia.   It's essential to consult an eye care professional to determine the most appropriate course of action for managing your or your child's myopia, as individual needs and circumstances may vary. |
| 1. **Why is myopia a concern? What are the potential complications of myopia?** | Myopia, or nearsightedness, is a concern because it can affect the quality of life by impairing clear distance vision. Additionally, as the degree of myopia increases, the risk of developing associated eye complications also rises. These complications can potentially lead to vision loss or even blindness in severe cases. Some of the potential complications of myopia include:   1. **Retinal detachment**: High myopia can cause the retina, the light-sensitive tissue at the back of the eye, to thin and stretch. This increases the risk of retinal detachment, a serious condition where the retina separates from the underlying supportive tissue. Retinal detachment can lead to permanent vision loss if not treated promptly. 2. **Myopic macular degeneration**: In high myopia, the stretched and thinned retina can lead to degeneration of the macula, the central part of the retina responsible for sharp, central vision. This can result in a gradual loss of central vision. 3. **Glaucoma**: Myopia, especially high myopia, is associated with an increased risk of developing glaucoma, a group of eye conditions characterized by damage to the optic nerve, often due to increased pressure within the eye. Glaucoma can lead to vision loss and blindness if not treated. 4. **Cataracts**: Myopia may increase the risk of developing cataracts, a clouding of the eye's natural lens, which can cause vision impairment. Cataracts can be surgically removed and replaced with artificial lenses. 5. **Strabismus**: Myopia can be associated with strabismus, a condition in which the eyes do not align properly, leading to one eye turning inward, outward, upward, or downward.   It is essential to have regular eye exams, especially for those with myopia, to monitor eye health and detect any complications early. Early detection and appropriate treatment can help prevent or minimize the impact of these complications on vision. |
| 1. **Can myopia cause blindness?** | Myopia itself does not directly cause blindness. However, high myopia (severe nearsightedness) can increase the risk of developing serious eye complications that can potentially lead to vision loss or even blindness if not treated appropriately. Some of the complications associated with high myopia that can contribute to vision loss include:   1. **Retinal detachment**: High myopia can cause the retina to thin and stretch, increasing the risk of retinal detachment. If not treated promptly, retinal detachment can lead to permanent vision loss. 2. **Myopic macular degeneration**: In high myopia, the stretched and thinned retina can lead to degeneration of the macula, the central part of the retina responsible for sharp, central vision. This can result in a gradual loss of central vision, severely impairing vision and, in extreme cases, causing legal blindness. 3. **Glaucoma**: Myopia, especially high myopia, is associated with an increased risk of developing glaucoma. Glaucoma damages the optic nerve, often due to increased pressure within the eye. If left untreated, glaucoma can lead to vision loss and blindness. 4. **Cataracts**: Although cataracts are not directly related to blindness, myopia may increase the risk of developing cataracts, which can impair vision. Cataracts can be surgically removed and replaced with artificial lenses.   It is essential to have regular eye exams, especially for those with high myopia, to monitor eye health and detect any complications early. Early detection and appropriate treatment can help prevent or minimize the impact of these complications on vision. |

# Supplementary Table 1c: Google Bard Responses to Myopia-Related Questions

| **Question** | **Google Bard Response** |
| --- | --- |
| **Topic: Pathogenesis** | |
| 1. **What is myopia?** | Myopia, also known as nearsightedness, is a common eye condition that affects your vision. People with myopia can see objects that are close to them clearly, but objects that are far away appear blurry. This is because the light rays entering the eye are not focused correctly on the retina, the back part of the eye that sends visual signals to the brain.  There are two main types of myopia:   1. **Simple myopia** is the most common type of myopia. It is caused by the eyeball being too long from front to back. 2. **Axial myopia** is caused by the cornea, the clear front part of the eye, being too curved.   Myopia is a complex condition with many causes, including:   1. **Genetics:** Myopia is often inherited from parents who have the condition. 2. **Environmental factors**: Spending a lot of time indoors, especially doing close-up activities such as reading, writing, or using electronic devices, can increase the risk of developing myopia. 3. **Age**: Myopia is most common in children and young adults. It usually stabilizes in the early 20s, but it can continue to worsen in some people.   Myopia can be treated with glasses, contact lenses, or surgery. Glasses and contact lenses help to bend the light rays entering the eye so that they are focused correctly on the retina. Surgery can reshape the cornea or the lens to correct myopia.  If you have myopia, it is important to see an eye doctor regularly to have your vision checked and to monitor the progression of your condition. Early detection and treatment can help to prevent serious eye problems such as glaucoma and cataracts. |
| 1. **At what age does myopia commonly develop?** | Myopia, also known as nearsightedness, is a common vision condition that affects the ability to see distant objects clearly. It is caused by the eye growing too long from front to back, which causes light to focus in front of the retina instead of on it. This results in blurry vision.  Myopia typically develops between the ages of 6 and 14. However, it can also develop in younger children or adults. In some cases, myopia may be present at birth.  There are a number of factors that can contribute to the development of myopia, including:   1. **Genetics**: Myopia is more common in people who have a family history of the condition. 2. **Environment**: Spending a lot of time indoors doing close-up activities, such as reading, writing, or using electronic devices, can increase the risk of developing myopia. 3. **Lifestyle**: Other factors that may contribute to the development of myopia include lack of outdoor activity, poor diet, and obesity.   If you are concerned that your child may have myopia, it is important to schedule an eye exam with an eye doctor. Early detection and treatment can help to prevent or slow the progression of myopia. |
| 1. **What causes myopia?** | The exact cause of myopia is unknown, but it is thought to be caused by a combination of genetic and environmental factors.  **Genetic factors:** Myopia is more common in people who have family members with the condition. This suggests that there is a genetic component to myopia.  **Environmental factors:** There are a number of environmental factors that have been linked to an increased risk of myopia, including:   1. Spending long periods of time doing close-up work, such as reading or using electronic devices 2. Lack of outdoor activity 3. Being born in an urban area 4. Having a high body mass index (BMI)   Myopia is a common condition that affects millions of people around the world. It is usually diagnosed in childhood and can worsen over time. There is no cure for myopia, but there are a number of treatments that can help to improve vision, such as glasses, contact lenses, and refractive surgery. |
| **Topic: Risk Factors** | |
| 1. **What puts my child at risk of myopia?** | There are many risk factors for myopia, including:   1. **Genetics:** If you or your child's other parent has myopia, your child is more likely to develop it. 2. **Ethnicity:** Myopia is more common in certain ethnic groups, such as East Asians and Southeast Asians. 3. **Age:** Myopia is more common in children and young adults. 4. **Near work:** Spending a lot of time doing near work, such as reading, writing, or using electronic devices, can increase the risk of myopia. 5. **Lack of outdoor time:** Spending less time outdoors can also increase the risk of myopia.   **Other factors:** Other factors that may increase the risk of myopia include:   1. **Low levels of vitamin D** 2. **Poor diet** 3. **Lack of sleep** 4. **Certain medical conditions**   If you are concerned that your child may be at risk of myopia, it is important to talk to your doctor. There are a number of things you can do to help reduce your child's risk of developing myopia, such as:   1. **Encourage your child to spend more time outdoors.** The more time your child spends outdoors, the less likely they are to develop myopia. 2. **Limit your child's near work time.** Encourage your child to take breaks from near work every 20-30 minutes. 3. **Make sure your child gets enough sleep.** Children and adolescents need 8-10 hours of sleep per night. 4. **Feed your child a healthy diet.** A healthy diet that is rich in fruits, vegetables, and whole grains can help reduce your child's risk of myopia. 5. **Get your child's eyes checked regularly.** Your child should have their eyes checked by an eye doctor at least once a year. |
| 1. **Is myopia hereditary? My spouse and I have myopia, how likely is it for my child to get myopia?** | Yes, myopia is hereditary. It is estimated that if both parents have myopia, their child has a 60-70% chance of developing myopia. If only one parent has myopia, the child's risk is still about 30-40%.  There are a number of genes that have been linked to myopia, but the exact way in which they work is not fully understood. It is thought that myopia is caused by a combination of genetic and environmental factors.  Environmental factors that may increase the risk of myopia include:   1. **Spending a lot of time indoors**, doing close-up work such as reading or using electronic devices 2. **Not spending enough time outdoors** 3. **Having a family history of myopia**   If you and your spouse both have myopia, there are a number of things you can do to help reduce your child's risk of developing the condition, including:   1. Encourage your child to spend at least two hours outdoors each day 2. Limit the amount of time your child spends doing close-up work 3. Make sure your child's diet is healthy and includes plenty of fruits and vegetables 4. Get your child's eyes checked regularly by an eye doctor   If your child does develop myopia, there are a number of treatments available to help them see clearly. These include glasses, contact lenses, and refractive surgery. |
| **Topic: Clinical Presentation** | |
| 1. **What are signs and symptoms that suggests my child has myopia?** | Myopia, also known as nearsightedness, is a common eye condition that makes it difficult to see objects that are far away. It is caused by the eye's inability to focus light properly on the retina, which is the light-sensitive layer at the back of the eye.  The most common signs and symptoms of myopia in children include:   1. Blurred vision when looking at distant objects 2. The need to squint or partially close the eyelids to see clearly 3. Headaches 4. Eyestrain 5. Sitting close to the television or computer 6. Difficulty seeing the chalkboard or whiteboard at school 7. Holding books or other objects close to the face 8. Frequent blinking or rubbing of the eyes   If you notice any of these signs or symptoms in your child, it is important to take them to an eye doctor for an evaluation. Myopia can be treated with eyeglasses, contact lenses, or surgery. Early treatment is important to prevent the condition from worsening.  Here are some tips to help prevent myopia in children:   1. **Encourage outdoor activities**: Spending time outdoors helps to reduce the risk of myopia by exposing the eyes to natural light. 2. **Limit screen time**: Children should limit their screen time to two hours per day. 3. **Get regular eye exams**: Children should have their eyes examined by an eye doctor every year.   If you have any concerns about your child's vision, please talk to their doctor. |
| **Topic: Diagnosis** | |
| 1. **How is myopia diagnosed?** | Myopia, also known as nearsightedness, is a common eye condition that causes distant objects to appear blurry. It is caused by the eye's inability to focus light properly on the retina, the light-sensitive layer at the back of the eye.  Myopia is diagnosed during a comprehensive eye exam. The eye doctor will perform a number of tests to determine the extent of your myopia and the best way to correct it. These tests may include:   1. **Visual acuity test:** This test measures your ability to see clearly at different distances. You will be asked to read letters or symbols on a chart placed at different distances from you. 2. **Refraction test:** This test measures the curvature of your cornea and lens, and the amount of refractive error you have. Refractive error is the difference between the shape of your eye and the way light is focused on your retina. 3. **Slit-lamp exam:** This test uses a microscope to examine the front of your eye, including your cornea, lens, and iris. 4. **Ophthalmoscopy:** This test uses a special light to examine the back of your eye, including your retina and optic nerve.   If you are diagnosed with myopia, your eye doctor will recommend the best treatment for you. This may include wearing glasses or contact lenses, or undergoing refractive surgery. |
| 1. **What should I do if I suspect my child has myopia?** | If you suspect that your child has myopia, it is important to take them to see an eye doctor as soon as possible. Myopia is a condition that causes blurred vision, and it can be difficult for children to tell if they have it. An eye doctor can perform a simple test to determine if your child has myopia, and they can also prescribe glasses or contact lenses to correct the vision.  There are a few things that you can do to help prevent myopia in your child. First, make sure that they spend plenty of time outdoors. Studies have shown that children who spend more time outdoors are less likely to develop myopia. Second, limit your child's screen time. Excessive screen time can contribute to the development of myopia. Finally, encourage your child to focus on objects that are far away. This can help to strengthen the muscles in their eyes and prevent myopia from developing.  If your child is diagnosed with myopia, there are a few things that you can do to help slow the progression of the condition. First, make sure that they wear their glasses or contact lenses as prescribed by their eye doctor. Second, encourage them to spend plenty of time outdoors. Finally, limit their screen time. By following these tips, you can help to keep your child's vision healthy.  Here are some additional tips for helping your child with myopia:   1. Make sure that your child's glasses or contact lenses fit properly and are up to date. 2. Encourage your child to read in a well-lit area. 3. Help your child to develop good study habits, such as taking breaks every 20 minutes to look at something far away. 4. If your child plays sports, make sure that they wear protective eyewear. 5. Talk to your child's eye doctor about other ways to slow the progression of myopia.   With your help, your child can live a healthy and active life with myopia. |
| **Topic: Treatment and Prevention** | |
| 1. **What can I do to prevent myopia?** | Myopia, also known as nearsightedness, is a common eye condition in which people can see objects clearly up close but have difficulty seeing objects that are far away. It is caused by the eye growing too long from front to back, which causes light to focus in front of the retina instead of on it.  There is no cure for myopia, but there are things you can do to slow its progression. Here are some tips:   1. **Spend more time outdoors:** Studies have shown that spending time outdoors, especially during childhood, can help to prevent or slow the progression of myopia. The exact reason for this is not fully understood, but it may be due to the fact that sunlight helps to regulate the growth of the eye. 2. **Reduce your near work:** If you spend a lot of time doing near work, such as reading, writing, or using electronic devices, take breaks every 20 minutes to look at something that is 20 feet away for 20 seconds. This will help to give your eyes a rest. 3. **Get regular eye exams:** is important to get regular eye exams, even if you do not have any symptoms of myopia. This is because myopia can develop gradually, and early detection and treatment can help to slow its progression. 4. **Wear glasses or contact lenses:** If you have myopia, wearing glasses or contact lenses can help you to see clearly. There are also some special types of glasses and contact lenses that can help to slow the progression of myopia.   If you are concerned about myopia, talk to your doctor. They can help you to develop a plan to slow the progression of this condition and protect your vision. |
| 1. **My child has myopia, what are the available treatments to slow down the progression of myopia?** | There are a number of treatments available to slow down the progression of myopia in children. These include:   1. **Atropine eye drops:** Atropine is a medication that can be used to dilate the pupils. It is also thought to slow down the progression of myopia by reducing the amount of time that the eye spends focused on near objects. Atropine eye drops are typically prescribed for children who are between the ages of 6 and 12 years old. 2. **Multifocal contact lenses:** Multifocal contact lenses are designed to provide clear vision at both near and far distances. They are thought to slow down the progression of myopia by reducing the amount of time that the eye spends focused on near objects. Multifocal contact lenses are typically prescribed for children who are between the ages of 8 and 12 years old. 3. **Orthokeratology (Ortho-K):** Ortho-K is a type of contact lens therapy that can be used to reshape the cornea, the clear front part of the eye. This can help to improve vision without the need for glasses or contact lenses. Ortho-K is typically prescribed for children who are between the ages of 8 and 12 years old. 4. **Reduced near work:** Children who spend a lot of time doing near work, such as reading, writing, or using electronic devices, are at an increased risk of developing myopia. Reducing the amount of time that children spend doing near work can help to slow down the progression of myopia. 5. **Increased outdoor activity:** Children who spend more time outdoors are less likely to develop myopia than children who spend less time outdoors. This is thought to be due to the fact that sunlight contains certain wavelengths of light that may help to protect the eyes from developing myopia.   It is important to talk to your child's eye doctor about the best treatment options for slowing down the progression of myopia. The best treatment for your child will depend on a number of factors, including the severity of their myopia, their age, and their lifestyle. |
| 1. **How can I prevent myopia/ myopia progression without the use of medications?** | There are a number of things you can do to prevent myopia or slow its progression without the use of medications. These include:   1. **Spend more time outdoors:** Studies have shown that spending time outdoors, especially during the day, can help to reduce the risk of developing myopia. The exact mechanism is not fully understood, but it is thought that exposure to sunlight may help to regulate the growth of the eye. 2. **Reduce screen time:** Excessive screen time has been linked to an increased risk of myopia. If you spend a lot of time looking at screens, try to take breaks every 20 minutes to look at something at least 20 feet away. 3. **Get regular eye exams:** If you have myopia, it is important to get regular eye exams so that your prescription can be updated as needed. Early intervention can help to slow the progression of myopia. 4. **Wear glasses or contact lenses:** Wearing glasses or contact lenses can help to improve your vision and reduce the strain on your eyes. This can help to slow the progression of myopia. 5. **Consider other treatments:** There are a number of other treatments that may be effective in preventing or slowing the progression of myopia. These include orthokeratology (a type of contact lens that reshapes the cornea) and atropine eye drops.   If you are concerned about myopia, talk to your doctor about the best way to prevent or slow its progression. |
| 1. **What are the spectacles/contact lenses available to prevent myopia/ myopia progression?** | There are a number of spectacles and contact lenses available to help prevent myopia or slow its progression. These include:   1. **Multifocal lenses:** These lenses have different powers in different areas, which helps to reduce the amount of time that the eye spends focusing on near objects. This can help to slow the growth of the eye and prevent myopia from worsening. 2. **Orthokeratology (ortho-K) lenses:** These lenses are worn overnight to reshape the cornea, which is the clear front part of the eye. This can help to improve vision and slow the progression of myopia. 3. **Atropine eye drops:** These drops can slow the growth of the eye by reducing the amount of light that reaches the retina. 4. **Contact lenses with defocus incorporated multiple segments (DIMS) technology:** These lenses have small areas of blur that are distributed across the lens surface. This helps to slow the growth of the eye and prevent myopia from worsening.   It is important to note that there is no one-size-fits-all solution for myopia prevention. The best option for you will depend on your individual needs and circumstances. It is important to talk to your eye doctor to find out which option is right for you.  Here are some additional tips for preventing myopia:   1. **Spend time outdoors**: Exposure to sunlight has been shown to slow the progression of myopia. Limit the amount of time that you spend indoors, especially in low-light conditions. 2. **Take regular breaks from near work**: Every 20 minutes, look at something that is at least 20 feet away for 20 seconds. 3. **Get enough sleep**: Sleep is important for eye growth and development. 4. **Eat a healthy diet**: A diet that is rich in fruits, vegetables, and whole grains can help to improve overall health and well-being, including eye health. |
| 1. **How effective is MiSight/ MiyoSmart/ Stellest/ Abiliti/ orthokeratology lenses at helping to prevent myopia/ myopia progression?** | The effectiveness of MiSight/ MiyoSmart/ Stellest/ Abiliti/ orthokeratology lenses at helping to prevent myopia/ myopia progression varies depending on the type of lens and the study. However, in general, these lenses have been shown to be effective in slowing the progression of myopia by about 30-60%.  **MiSight (CooperVision)**: A multifocal soft contact lens that has been shown to slow the progression of myopia by up to 60% in children and adolescents.  **MiyoSmart (Hoya)**: A spectacle lens that uses a diffractive optical technology to slow the progression of myopia by up to 59% in children and adolescents.  **Stellest (Essilor)**: A spectacle lens that uses a holographic optical element to slow the progression of myopia by up to 65% in children and adolescents.  **Abiliti (Johnson & Johnson)**: A soft contact lens that uses a combination of atropine and a defocus-inducing design to slow the progression of myopia by up to 59% in children and adolescents.  **Orthokeratology**: A non-surgical procedure that uses specially designed contact lenses to reshape the cornea and slow the progression of myopia.  It is important to note that these lenses are not a cure for myopia, and they will not stop the progression of myopia completely. However, they can be an effective way to slow the progression of myopia and help to reduce the risk of long-term eye health problems associated with myopia, such as glaucoma and cataracts.  If you are concerned about your child's myopia, talk to your eye doctor about the different options available to help slow the progression of myopia. |
| 1. **How does MiSight/ MiyoSmart/ Stellest/ Abiliti/ orthokeratology lenses help to prevent myopia/ myopia progression?** | Myopia, also known as nearsightedness, is a common eye condition that affects the way the eye focuses light. In people with myopia, the eye is too long from front to back, or the cornea (the clear front part of the eye) is too curved. This causes light to focus in front of the retina, rather than directly on it. As a result, objects that are far away appear blurry.  Myopia is a progressive condition, which means that it can get worse over time. The risk of myopia progression is highest in children and adolescents. There are a number of factors that can contribute to myopia progression, including genetics, environment, and lifestyle.  There are a number of ways to help prevent myopia progression. One of the most effective methods is to use myopia control lenses. Myopia control lenses are designed to slow the growth of the eye and prevent myopia from getting worse.  There are a number of different types of myopia control lenses available. Some of the most common types include:   1. **Defocus Incorporated Multiple Segments (DIMS) lenses:** DIMS lenses are spectacle lenses that have a series of small, ring-shaped areas that create a blur in front of the retina. This blur helps to slow the growth of the eye and prevent myopia progression. 2. **Orthokeratology (Ortho-K) lenses:** Ortho-K lenses are contact lenses that are worn overnight. They reshape the cornea, which helps to improve vision and slow the growth of the eye. 3. **Atropine eye drops:** Atropine is a medication that is used to dilate the pupils. It can be used to slow the growth of the eye and prevent myopia progression.   If you are concerned about your child's risk of myopia progression, talk to your eye doctor. They can recommend the best treatment option for your child.  Here are some additional details about the different types of myopia control lenses:   1. **DIMS lenses:** DIMS lenses are the most common type of myopia control lens. They are available in both single-vision and progressive designs. DIMS lenses have been shown to be effective in slowing the progression of myopia in children and adolescents. 2. **Orthokeratology (Ortho-K) lenses:** Ortho-K lenses are a type of contact lens that is worn overnight. They reshape the cornea, which helps to improve vision and slow the growth of the eye. Ortho-K lenses have been shown to be effective in slowing the progression of myopia in children and adolescents. 3. **Atropine eye drops:** Atropine is a medication that is used to dilate the pupils. It can be used to slow the growth of the eye and prevent myopia progression. Atropine eye drops are less effective than DIMS lenses or Ortho-K lenses, but they may be a good option for children who are not able to wear contact lenses.   If you are concerned about your child's risk of myopia progression, talk to your eye doctor. They can recommend the best treatment option for your child. |
| 1. **Is it safe for my child to be using contact lens at such a young age?** | There is no minimum age for wearing contact lenses, but eye care providers generally recommend that children be at least 8 years old before getting them. This is because younger children may not be able to handle the responsibility of taking care of their contact lenses properly. Contact lenses can be a safe and effective way to correct vision problems in children, but it is important to make sure that your child is ready for them before getting them.  Here are some things to consider when deciding if your child is ready for contact lenses:   1. **Their age and maturity level.** Children need to be old enough and responsible enough to handle the daily care of their contact lenses. This includes being able to put them in and take them out correctly, as well as cleaning and storing them properly. 2. **Their eye health.** Children with certain eye conditions, such as dry eyes or allergies, may not be good candidates for contact lenses. 3. **Their lifestyle.** Children who are active or participate in sports may be more likely to damage their contact lenses.   If you are considering getting contact lenses for your child, it is important to talk to your eye care provider. They can help you determine if your child is a good candidate for contact lenses and can provide you with instructions on how to care for them properly.  Here are some additional tips for keeping your child's contact lenses safe:   1. **Wash your hands thoroughly before handling contact lenses.** 2. **Use only the cleaning solution and storage case that your eye care provider recommends.** 3. **Never reuse contact lens solution.** 4. **Replace your contact lenses according to your eye care provider's instructions.** 5. **See your eye care provider for regular eye exams.**   By following these tips, you can help keep your child's eyes healthy and safe while they wear contact lenses. |
| 1. **How effective is reducing screen time/ increasing outdoor time at helping to prevent myopia/ myopia progression?** | There is a growing body of evidence that suggests that reducing screen time and increasing outdoor time can help to prevent myopia and myopia progression. Myopia is a condition in which the eye is too long from front to back, causing light to focus in front of the retina instead of on it. This results in blurred vision, especially for objects that are far away. Myopia is a common condition, affecting about 30% of the world's population. It is more common in East Asia, where up to 90% of people are myopic.  There are a number of factors that are thought to contribute to the development of myopia, including genetics, environment, and lifestyle. One of the most important environmental factors is the amount of time spent indoors, looking at near objects, such as screens and books. This is because when we focus on near objects, our eyes have to work harder to see clearly. This extra work can cause the eye to grow longer, leading to myopia.  On the other hand, spending time outdoors has been shown to protect against myopia. This is thought to be because sunlight helps to stimulate the production of dopamine, a neurotransmitter that helps to keep the eye from growing too long.  A number of studies have shown that reducing screen time and increasing outdoor time can help to prevent myopia. For example, a study of children in Singapore found that those who spent at least two hours per day outdoors were 30% less likely to develop myopia than those who spent less than one hour per day outdoors. Another study of children in China found that those who spent at least two hours per day outdoors had a 23% slower progression of myopia than those who spent less than one hour per day outdoors.  The amount of time that children need to spend outdoors to protect against myopia is not yet known. However, it is thought that at least two hours per day is beneficial. It is also important to note that spending time outdoors is not a cure for myopia. If a child already has myopia, spending time outdoors may help to slow the progression of the condition, but it will not reverse it.  There are a number of other things that parents can do to help prevent myopia in their children, such as:   1. Encourage them to spend time outdoors. 2. Limit the amount of time they spend looking at near objects, such as screens and books. 3. Make sure they have good lighting when they are reading or doing other activities that require them to look at near objects. 4. Get them regular eye exams so that any signs of myopia can be detected early.   By following these tips, parents can help to protect their children from myopia. |
| 1. **What is the maximum duration of screen time/ near work per day before my child is at risk of myopia/ myopia progression?** | There is no one-size-fits-all answer to this question, as the risk of myopia and myopia progression varies from child to child. However, the American Academy of Ophthalmology (AAO) recommends that children limit their screen time to two hours per day. The AAO also recommends that children take breaks from screen time every 20 minutes to look at something at least 20 feet away.  Here are some other things that parents can do to help reduce their child's risk of myopia:   1. **Encourage outdoor activities**: Children who spend more time outdoors are less likely to develop myopia. 2. **Make sure your child's eyes are healthy**: Have your child's eyes checked by an eye doctor regularly. 3. **Provide your child with a healthy diet**: Eating a healthy diet can help to reduce the risk of myopia. 4. **Get regular exercise**: Exercise can help to reduce the risk of myopia.   If you are concerned about your child's risk of myopia, talk to your child's eye doctor. |
| 1. **How much outdoor time does my child require to prevent myopia/ myopia progression?** | Studies have shown that spending at least 13 hours per week (just under 2 hours per day) outdoors can help to prevent myopia in children. This is thought to be due to the fact that bright sunlight helps to stimulate the production of dopamine, a neurotransmitter that is involved in eye growth.  For children who are already myopic, spending more time outdoors can help to slow the progression of the condition. A study published in the journal Ophthalmology found that children who spent an average of 76 minutes per day outdoors had a 50% lower risk of developing myopia than those who spent less time outdoors.  It is important to note that spending time outdoors is not a guarantee that your child will not develop myopia. However, it is a safe and effective way to reduce the risk of the condition.  Here are some tips for encouraging your child to spend more time outdoors:   1. Make outdoor activities a part of your family's routine. Go for walks, bike rides, or play catch in the yard. 2. Send your child to outdoor playdates or camps. 3. Encourage your child to spend time outdoors during the summer months. 4. Make sure your child wears sunglasses and sunscreen when they are outdoors.   By following these tips, you can help to keep your child's eyes healthy and prevent myopia. |
| 1. **What type of diet/ vitamin supplementation can help to prevent myopia/ myopia progression?** | There is no one-size-fits-all answer to this question, as the best diet and vitamin supplementation for myopia prevention will vary depending on the individual's specific needs and circumstances. However, some general dietary and vitamin supplementation tips that may help to prevent myopia include:   1. Eating a healthy diet that is rich in fruits, vegetables, and whole grains. These foods are packed with essential nutrients that are important for eye health, including vitamins A, C, E, zinc, and omega-3 fatty acids. 2. Getting regular exercise. Exercise has been shown to help improve eye health and reduce the risk of myopia. 3. Spending less time indoors and more time outdoors. Exposure to sunlight helps to protect the eyes from harmful UV rays, which can contribute to the development of myopia. 4. Wearing sunglasses when outdoors to protect the eyes from UV rays. 5. Getting regular eye exams. This is important for early detection of myopia and other eye problems. 6. In addition to following a healthy diet and lifestyle, some people may also benefit from taking vitamin supplements. Some studies have shown that certain vitamins, such as lutein, zeaxanthin, and omega-3 fatty acids, may help to slow the progression of myopia. However, more research is needed to confirm these findings.   If you are concerned about myopia, it is important to talk to your doctor. They can help you develop a personalized plan to prevent or slow the progression of this condition. |
| 1. **Are there any available vision therapy/ eye exercises that can help to prevent myopia/ myopia progression?** | Yes, there are a number of vision therapy exercises that can help to prevent or slow the progression of myopia. These exercises typically focus on improving the following visual skills:   1. **Accommodation**: This is the ability of the eyes to focus on objects at different distances. 2. **Convergence**: This is the ability of the eyes to work together to focus on an object. 3. **Vergence**: This is the ability of the eyes to move in opposite directions to maintain focus on an object. 4. **Accommodative-convergence reflex (ACR)**: This is the reflex that helps to keep the eyes focused on an object as they move closer or further away. 5. **Peripheral awareness**: This is the ability to see objects in your peripheral vision.   Some of the most common vision therapy exercises for myopia include:   1. **Pencil push-ups**: This exercise helps to improve accommodation. Hold a pencil at arm's length and focus on the tip of the pencil. Slowly bring the pencil closer to your face, keeping your eyes focused on the tip. When the pencil is about 4 inches from your face, stop and rest your eyes for a few seconds. Repeat this exercise 10-15 times. 2. **Palming**: This exercise helps to improve convergence. Place your palms over your closed eyes and rest for 30 seconds. Repeat this exercise 3-5 times. 3. **Bilateral eye exercises**: These exercises help to improve vergence. There are a variety of bilateral eye exercises, but some common ones include alternating eye patching, pencil push-ups with both eyes open, and following a moving object with both eyes. 4. **Peripheral awareness exercises**: These exercises help to improve peripheral awareness. There are a variety of peripheral awareness exercises, but some common ones include looking around a room while keeping your head still, and reading a book while covering your peripheral vision with your hands.   It is important to note that vision therapy is not a cure for myopia. However, it can help to slow the progression of myopia and improve visual function. If you are concerned about myopia, talk to your eye doctor about vision therapy. |
| 1. **What medications are available to prevent myopia/ myopia progression?** | There are a number of medications that are available to help prevent or slow the progression of myopia. These include:   1. **Atropine eye drops:** Atropine is a medication that is used to dilate the pupils. It is also effective in slowing the progression of myopia. Atropine eye drops are typically applied once a day at night. 2. **Orthokeratology (Ortho-K) lenses:** Ortho-K lenses are a type of contact lens that is worn overnight. They reshape the cornea, which helps to improve vision during the day. Ortho-K lenses can be effective in slowing the progression of myopia. 3. **Multifocal contact lenses:** Multifocal contact lenses have different powers in different parts of the lens. This helps to improve vision at both near and far distances. Multifocal contact lenses can be effective in slowing the progression of myopia. 4. **Pilocarpine eye drops:** Pilocarpine is a medication that is used to treat glaucoma. It can also be effective in slowing the progression of myopia. Pilocarpine eye drops are typically applied twice a day.   It is important to talk to your doctor about the best medication for you. The best medication for you will depend on your individual needs and preferences. |
| 1. **Is atropine safe in children?** | Yes, atropine eye drops are generally safe for children when used as prescribed by a doctor. Atropine is a medication that is used to dilate the pupils and relax the muscles of the eye. It is often used to treat myopia (nearsightedness) in children.  Atropine eye drops are typically given once a day at bedtime. The drops will cause the pupils to dilate, which will make it more difficult for the child to see in the dark. This is why it is important to give the drops at bedtime.  The most common side effects of atropine eye drops in children are:   1. Blurred vision 2. Dry eyes 3. Sensitivity to light 4. Headache 5. Itching or redness in the eye   In rare cases, atropine eye drops can cause more serious side effects, such as:   1. Allergic reaction 2. Glaucoma 3. Cataracts   If you notice any side effects after your child starts using atropine eye drops, be sure to talk to your doctor.  It is important to note that atropine eye drops are not a cure for myopia. They will help to slow the progression of the condition, but they will not stop it altogether. If your child is diagnosed with myopia, it is important to talk to your doctor about the best treatment options for them. |
| 1. **What are the side effects of atropine?** | Atropine is a medication that is used to treat a variety of conditions, including bradycardia, stomach cramps, and motion sickness. It can also be used to dilate the pupils before eye exams or surgery. Atropine is a member of a class of drugs called anticholinergics. These drugs work by blocking the effects of acetylcholine, a neurotransmitter that is involved in many bodily functions.  The most common side effects of atropine are:   1. Dry mouth 2. Blurred vision 3. Flushing 4. Increased heart rate 5. Sweating 6. Difficulty urinating 7. Constipation 8. Headache 9. Dizziness 10. Nervousness 11. Restlessness 12. Hallucinations   In some cases, atropine can cause more serious side effects, such as:   1. High fever 2. Seizures 3. Coma 4. Heart attack 5. Stroke   If you experience any of these side effects, it is important to seek medical attention immediately.  Here are some additional things to keep in mind about the side effects of atropine:   1. The risk of side effects increases with the dose of atropine. 2. Older adults and people with certain medical conditions, such as heart disease or glaucoma, are more likely to experience side effects from atropine. 3. Side effects from atropine are usually temporary and go away on their own within a few hours. 4. If you experience any bothersome side effects, talk to your doctor about ways to reduce them.   It is important to note that this is not a complete list of side effects for atropine. If you have any concerns, be sure to talk to your doctor. |
| 1. **How effective is atropine at helping to prevent myopia/ myopia progression?** | Atropine is a medication that is used to treat a variety of conditions, including myopia. Myopia is a condition in which the eye is too long from front to back, causing light to focus in front of the retina instead of on it. This results in blurry vision, especially for objects that are far away.  Atropine works by relaxing the ciliary muscle, which is responsible for focusing the eye. This helps to reduce the amount of myopia that develops over time. Atropine is most effective when it is started in children who are at high risk for developing myopia.  Studies have shown that atropine can be effective in slowing the progression of myopia in children. In one study, children who used atropine eye drops 0.01% twice a day for one year had a 50% reduction in myopia progression compared to children who used a placebo.  Atropine is generally considered to be safe and well-tolerated. However, some side effects may occur, such as blurred vision, light sensitivity, and dry eyes. These side effects are usually mild and go away on their own.  If you are considering using atropine to help prevent myopia, talk to your doctor about the risks and benefits. |
| 1. **What concentration of atropine is available, and which is the most effective?** | Atropine is available in a variety of concentrations, including 0.01%, 0.025%, 0.05%, and 1%. The most effective concentration for myopia control is still being studied, but 0.05% atropine appears to be the most effective, with the fewest side effects.  A study published in the journal Ophthalmology found that 0.05% atropine was more effective than 0.01% atropine at slowing the progression of myopia in children. The study also found that 0.05% atropine was associated with fewer side effects than 0.01% atropine.  Another study, published in the journal Investigative Ophthalmology & Visual Science, found that 0.05% atropine was more effective than 1% atropine at slowing the progression of myopia in children. The study also found that 0.05% atropine was associated with fewer side effects than 1% atropine.  Based on these studies, it appears that 0.05% atropine is the most effective concentration for myopia control. It is also associated with the fewest side effects. However, more research is needed to confirm these findings.  It is important to note that atropine eye drops are not a cure for myopia. They can only slow the progression of the condition. If you are considering using atropine eye drops, it is important to talk to your doctor about the risks and benefits. |
| 1. **How does atropine help to prevent myopia/ myopia progression?** | Atropine is a medication that is used to relax the muscles in the eye. It is commonly used to treat eye conditions such as glaucoma and iritis. Atropine can also be used to prevent myopia, or nearsightedness.  Myopia is a condition in which the eye is too long from front to back. This causes light to focus in front of the retina, instead of directly on it. This results in blurred vision, especially for objects that are far away.  Atropine works to prevent myopia by slowing down the growth of the eye. It does this by blocking the effects of acetylcholine, a neurotransmitter that is involved in eye growth. Atropine can also help to reduce the amount of time that children spend squinting, which can also contribute to the development of myopia.  Atropine is a safe and effective treatment for myopia. It is typically used in eye drops that are applied once or twice a day. The most common side effects of atropine are blurred vision, light sensitivity, and dry eyes. These side effects are usually mild and go away on their own.  Atropine is a good option for children who are at risk for developing myopia. It can help to slow down the progression of myopia and prevent vision problems later in life. |
| 1. **My child has not developed myopia, should he/ she start using atropine?** | No, your child should not start using atropine if they have not developed myopia. Atropine is a medication that is used to slow the progression of myopia, but it is not a cure. If your child does not have myopia, there is no need to give them atropine.  There are a number of things that you can do to help prevent your child from developing myopia, such as:   1. Ensuring that they get enough outdoor time. 2. Limiting their close-up activities, such as reading, playing video games, and using electronic devices. 3. Ensuring that they have good vision habits, such as sitting up straight and focusing on objects that are at a distance.   If you are concerned about your child's risk of developing myopia, you should talk to their doctor. They can assess your child's risk factors and recommend ways to help prevent myopia. |
| 1. **My child is on atropine for myopia control, when can it be stopped?** | The decision of when to stop atropine for myopia control in children is a complex one that should be made on a case-by-case basis. There is no one-size-fits-all answer, as the decision will depend on a number of factors, including the child's age, the severity of their myopia, and the response to treatment.  In general, atropine eye drops are most effective in slowing the progression of myopia in children between the ages of 6 and 12. After this age, the effectiveness of atropine eye drops decreases.  If your child is responding well to atropine eye drops and their myopia is not progressing rapidly, you may want to continue treatment for as long as possible. However, if your child is experiencing side effects from the eye drops, or if their myopia is progressing rapidly, you may want to consider stopping treatment.  It is important to talk to your child's eye doctor about the best time to stop atropine eye drops for myopia control. They will be able to assess your child's individual situation and make the best recommendation for your child.  Here are some additional things to consider when deciding when to stop atropine eye drops:   1. The child's age and maturity level. Younger children may have difficulty remembering to apply the eye drops on their own, and they may also be more likely to experience side effects. 2. The severity of the child's myopia. The more severe the myopia, the more likely it is to progress rapidly. 3. The response to treatment. If the child is not responding well to atropine eye drops, or if their myopia is progressing rapidly, you may want to consider stopping treatment. 4. The child's lifestyle. If the child is very active and spends a lot of time outdoors, they may be at a lower risk of developing myopia.   Ultimately, the decision of when to stop atropine eye drops is a personal one. You should weigh the risks and benefits of treatment and make the decision that is best for your child. |
| **Topic: Prognosis** | |
| 1. **Is myopia reversible?** | No, myopia cannot be reversed. It is a condition in which the eye is too long from front to back, causing light to focus in front of the retina instead of on it. This results in blurred vision at a distance. Myopia is caused by a combination of genetic and environmental factors, and it is most common in children and young adults.  There are a number of ways to manage myopia, including:   1. Wearing glasses or contact lenses to correct the vision. 2. Using atropine eye drops to slow the progression of myopia. 3. Participating in a myopia control program, which may include wearing special contact lenses or doing vision therapy exercises. 4. Having laser eye surgery, such as LASIK or PRK, to correct the vision.   However, none of these treatments can reverse myopia. If you have myopia, it is important to see an eye doctor regularly to monitor your vision and make sure that your treatment plan is working. |
| 1. **Why is myopia a concern? What are the potential complications of myopia?** | Myopia, also known as nearsightedness, is a common eye condition that causes distant objects to appear blurry. It occurs when the eye is too long from front to back, or the cornea (the clear front part of the eye) is too curved. This causes light to focus in front of the retina, instead of directly on it. The retina is a light-sensitive layer of tissue at the back of the eye that sends signals to the brain to create vision.  Myopia is a concern because it can lead to a number of serious eye problems, including:   1. **Myopic macular degeneration (MMD)**: This is a condition that damages the macula, the part of the retina responsible for central vision. MMD can cause blurry vision, distorted vision, and blind spots. 2. **Retinal detachment:** This is a serious condition that occurs when the retina pulls away from the back of the eye. If not treated promptly, retinal detachment can lead to permanent blindness. 3. **Cataracts:** Cataracts are cloudy areas that develop in the lens of the eye. They can cause blurry vision, glare, and halos around lights. 4. **Open-angle glaucoma:** This is a condition that occurs when the pressure inside the eye is too high. It can damage the optic nerve, the nerve that carries visual information from the eye to the brain. If left untreated, glaucoma can lead to blindness.   The risk of developing these complications increases with the severity of myopia. For example, people with high myopia (-5 diopters or more) are at a much higher risk of developing MMD, retinal detachment, and cataracts than people with mild myopia.  There is no cure for myopia, but there are a number of things that can be done to manage the condition and prevent complications. These include:   1. Wearing glasses or contact lenses to correct the vision. 2. Getting regular eye exams to monitor the condition for signs of complications. 3. Reducing the risk factors for myopia, such as spending too much time indoors and not getting enough outdoor activity.   If you have myopia, it is important to talk to your eye doctor about how to manage the condition and prevent complications. |
| 1. **Can myopia cause blindness?** | In most cases, myopia (nearsightedness) does not cause blindness. However, in some cases, high myopia (myopia that is severe) can lead to serious eye conditions that can cause blindness. These conditions include:   1. **Macular degeneration:** This is a condition that damages the macula, the part of the retina responsible for central vision. Macular degeneration can cause blurry vision, blind spots, and eventually, legal blindness. 2. **Retinal detachment:** This is a condition that occurs when the retina, the light-sensitive layer at the back of the eye, tears or pulls away from the underlying tissue. Retinal detachment can cause sudden, severe vision loss. 3. **Glaucoma:** This is a condition that damages the optic nerve, the nerve that carries visual information from the eye to the brain. Glaucoma can cause blurry vision, tunnel vision, and eventually, blindness. 4. **Cataracts:** This is a clouding of the lens of the eye. Cataracts can cause blurry vision, glare, and halos.   If you have high myopia, it is important to see an eye doctor regularly for eye exams. This will help your doctor to detect any eye conditions early on and treat them before they cause permanent vision loss.  There are a number of things that you can do to reduce your risk of developing serious eye conditions from myopia, including:   1. **Get regular eye exams.** 2. **Wear glasses or contact lenses to correct your vision.** 3. **Spend time outdoors.** 4. **Eat a healthy diet.** 5. **Quit smoking.** 6. **Control your blood pressure.**   If you have any concerns about your vision, be sure to see an eye doctor. |

# Supplementary Table 2: Raw Score and Rating of each LLM-Chatbot, Attained for Each Question

| Domain | Question No. | ChatGPT-3.5 | | | ChatGPT-4.0 | | | Google Bard | | |
| --- | --- | --- | --- | --- | --- | --- | --- | --- | --- | --- |
|  |  | ***Score Sum (Acc) Max: 9*** | ***Majority Consensus (Acc)*** | ***Average Score (Comp) Max: 5*** | ***Score Sum (Acc) Max: 9*** | ***Majority Consensus (Acc)*** | ***Average Score (Comp) Max: 5*** | ***Score Sum (Acc) Max: 9*** | ***Majority Consensus (Acc)*** | ***Average Score (Comp) Max: 5*** |
| Pathogenesis | 1 | 8 | Good | 4.00 | 9 | Good | 3.33 | 4 | Poor | - |
|  | 2 | 9 | Good | 3.67 | 9 | Good | 4.00 | 9 | Good | 3.67 |
|  | 3 | 8 | Good | 3.00 | 9 | Good | 3.33 | 8 | Good | 4.00 |
| Risk Factors | 1 | 8 | Good | 4.50 | 9 | Good | 4.00 | 7 | Borderline | - |
|  | 2 | 8 | Good | 5.00 | 8 | Good | 4.50 | 8 | Good | 4.50 |
| Clinical Presentation | 1 | 9 | Good | 4.67 | 9 | Good | 4.67 | 8 | Good | 5.00 |
| Diagnosis | 1 | 8 | Good | 4.50 | 8 | Good | 4.50 | 9 | Good | 4.33 |
|  | 2 | 9 | Good | 4.00 | 8 | Good | 5.00 | 6 | Poor* | - |

| Treatment and Prevention | 1 | 6 | Borderline | - | 9 | Good | 4.00 | 8 | Good | 4.50 |
| --- | --- | --- | --- | --- | --- | --- | --- | --- | --- | --- |
|  | 2 | 5 | Borderline | - | 7 | Borderline | - | 7 | Borderline | - |
|  | 3 | 7 | Borderline | - | 9 | Good | 3.33 | 7 | Borderline | - |
|  | 4 | 6 | Borderline | - | 6 | Poor* | - | 5 | Borderline | - |
|  | 5 | 3 | Poor | - | 8 | Good | 4.50 | 9 | Good | 4.67 |
|  | 6 | 3 | Poor | - | 6 | Borderline | - | 7 | Borderline |  |
|  | 7 | 9 | Good | 4.00 | 9 | Good | 3.33 | 9 | Good | 5.00 |
|  | 8 | 9 | Good | 4.00 | 8 | Good | 4.50 | 8 | Good | 4.50 |
|  | 9 | 9 | Good | 4.67 | 9 | Good | 5.00 | 8 | Good | 4.50 |
|  | 10 | 8 | Good | 5.00 | 9 | Good | 5.00 | 8 | Good | 5.00 |
|  | 11 | 6 | Poor* | - | 5 | Poor | - | 5 | Poor | - |
|  | 12 | 8 | Good | 3.00 | 7 | Borderline | - | 4 | Poor | - |
|  | 13 | 5 | Borderline | - | 9 | Good | 3.67 | 4 | Poor | - |
|  | 14 | 7 | Borderline | - | 9 | Good | 4.33 | 6 | Borderline | - |
|  | 15 | 8 | Good | 4.00 | 8 | Good | 2.00 | 8 | Good | 2.00 |
|  | 16 | 8 | Good | 3.50 | 9 | Good | 5.00 | 7 | Borderline | - |
|  | 17 | 7 | Borderline | - | 9 | Good | 4.67 | 8 | Good | 4.50 |
|  | 18 | 6 | Poor* | - | 8 | Good | 5.00 | 5 | Borderline | - |
|  | 19 | 6 | Poor* | - | 6 | Poor* | - | 5 | Borderline | - |
|  | 20 | 9 | Good | 4.33 | 9 | Good | 4.00 | 8 | Good | 4.00 |
| Prognosis | 1 | 9 | Good | 4.67 | 8 | Good | 5.00 | 9 | Good | 4.67 |
|  | 2 | 8 | Good | 2.50 | 9 | Good | 4.67 | 8 | Good | 4.50 |
|  | 3 | 9 | Good | 5.00 | 9 | Good | 4.33 | 9 | Good | 4.67 |

*Where consensus on final accuracy rating was not reached (i.e. each grader provided a different rating), the lowest score (*‘poor’)* was assigned.

# Supplementary Table 3a: Demonstration of ChatGPT-3.5’s Ability to Self-Correct when Prompted

| **Topic: Treatment and Prevention** | | | | |
| --- | --- | --- | --- | --- |
| **Question:** | 1. **How effective is MiSight/ MiyoSmart/ Stellest/ Abiliti/ orthokeratology lenses at helping to prevent myopia/ myopia progression?** | | | |
| Initial Response: | MiSight, MiyoSmart, Stellest, Abiliti, and orthokeratology lenses are all options that have been shown to be effective in slowing down the progression of myopia in children.  **MiSight**: MiSight is a soft contact lens that is specifically designed to correct myopia and slow down its progression in children. Clinical studies have shown that it can slow the progression of myopia by up to 59% over three years.  **MiyoSmart**: MiyoSmart is another soft contact lens designed to correct myopia and slow down its progression. It features a unique concentric ring design that has been shown to be effective in slowing down myopia progression by up to 60%.  **Stellest**: Stellest is a spectacle lens that has a central area for distance vision and a peripheral area for near vision. Clinical studies have shown that it can slow down myopia progression by up to 67% over two years.  **Abiliti**: Abiliti is an implantable device that is surgically implanted in the eye. It uses low-level electrical pulses to help slow down the progression of myopia. Clinical studies have shown that it can reduce myopia progression by up to 59% over two years.  **Orthokeratology lenses**: Orthokeratology (Ortho-k) lenses are gas permeable contact lenses that are worn overnight to temporarily reshape the cornea. Studies have shown that they can reduce myopia progression by up to 50%.  It's important to note that the effectiveness of these treatments can vary depending on several factors, such as the severity of the myopia, the age of the child, and the length of treatment. Consult with an optometrist or ophthalmologist to determine the most appropriate treatment option for your child based on their specific situation. They can provide you with more information about the available options and help you make an informed decision. | | | |
| Initial Grades: | Sum of Accuracy Scores (Max 9): | 3 | Majority Consensus: | Poor |
| Self-Corrected Response: | **MiSight**: MiSight is a soft contact lens specifically designed for myopia control in children. It utilizes a center-distance design with peripheral myopic defocus, which has been shown to slow down the progression of myopia. Clinical studies have demonstrated its effectiveness in reducing myopia progression by approximately 59% over a three-year period.  **MiyoSmart**: MiyoSmart is another soft contact lens designed for myopia control. It incorporates a novel D.I.M.S. (Defocus Incorporated Multiple Segments) technology that creates myopic defocus in the peripheral retina. Clinical trials have shown it to be effective in reducing myopia progression by about 52% over a two-year period.  **Stellest**: Stellest is an eyeglass lens developed by Essilor. It utilizes a sophisticated optical design involving an array of micro-lenses to create myopic defocus in the peripheral vision. Studies have demonstrated its ability to slow down myopia progression by approximately 67% over a one-year period.  **Abiliti**: Abiliti is a new technology that involves an implantable device placed in the eye. It uses tiny microlenses to create a ring of peripheral myopic defocus, which is believed to help slow down myopia progression. However, at the time of my knowledge cutoff in September 2021, there was limited information available about its effectiveness and widespread availability.  **Orthokeratology (Ortho-K)**: Ortho-K involves the use of specially designed rigid gas-permeable contact lenses worn during sleep. These lenses temporarily reshape the cornea, providing clear vision during the day without the need for glasses or contact lenses. Ortho-K has shown potential for reducing myopia progression, but its long-term effects and individual variability in response require further research.  It's important to consult with an eye care professional to determine the most suitable treatment option based on your specific needs and circumstances. They will consider factors such as age, degree of myopia, and overall eye health to guide you in choosing an appropriate treatment for myopia control. | | | |
| Self-Corrected Grades: | Sum of Accuracy Scores (Max 9): | 4 | Majority Consensus: | Poor |
|  | | | | |
| **Question:** | 1. **How does MiSight/ MiyoSmart/ Stellest/ Abiliti/ orthokeratology lenses help to prevent myopia/ myopia progression?** | | | |
| Initial Response: | MiSight, MiyoSmart, Stellest, Abiliti, and orthokeratology lenses all work in different ways to help prevent myopia or slow down its progression.  **MiSight and MiyoSmart**: These are soft contact lenses that have a unique concentric ring design. The rings create a distance center and a peripheral zone that provides myopic defocus, which has been shown to slow down the elongation of the eye that causes myopia progression. The lenses also correct the child's vision while they wear them.  **Stellest**: Stellest is a spectacle lens that also uses myopic defocus to slow down myopia progression. The lens has a central area for distance vision and a peripheral area for near vision. The peripheral area is designed to create myopic defocus, which has been shown to slow down the elongation of the eye that causes myopia progression.  **Abiliti**: Abiliti is an implantable device that is surgically implanted in the eye. It uses low-level electrical pulses to stimulate the ciliary muscle, which controls the shape of the lens in the eye. This stimulation can help to reduce the elongation of the eye that causes myopia progression.  **Orthokeratology lenses**: Orthokeratology (Ortho-k) lenses are gas permeable contact lenses that are worn overnight to temporarily reshape the cornea. The reshaping of the cornea can help to correct myopia and slow down its progression. The lenses are worn overnight and removed in the morning, providing clear vision during the day without the need for eyeglasses or contact lenses.  Overall, these treatments all aim to provide clear vision while also creating myopic defocus, which has been shown to slow down the elongation of the eye that causes myopia progression. Consult with an optometrist or ophthalmologist to determine the most appropriate treatment option for your child based on their specific situation. | | | |
| Initial Grades: | Sum of Accuracy Scores (Max 9): | 3 | Majority Consensus: | Poor |
| Self-Corrected Response: | **MiSight**: MiSight is a type of soft contact lens approved by the U.S. Food and Drug Administration (FDA) for the correction of myopia and its progression in children aged 8 to 12 at the initiation of treatment. It uses a dual-focus design with alternating vision correction and treatment zones. The treatment zone creates myopic defocus on the peripheral retina, which is believed to slow down the progression of myopia over time.  **MiyoSmart**: MiyoSmart is another soft contact lens designed for myopia control in children. It utilizes a similar dual-focus design as MiSight but incorporates additional features. MiyoSmart lenses have a central correction zone for clear distance vision and multiple concentric rings in the treatment zone, which also induce myopic defocus in the periphery. Studies have shown that MiyoSmart can effectively slow down the progression of myopia.  **Stellest**: Stellest lenses are spectacle lenses developed for myopia management in children. They use a novel optical design called extended depth of focus (EDOF). This design creates myopic defocus in the peripheral retina while providing clear vision for both near and distance objects. By stimulating the peripheral retina, Stellest lenses aim to slow down the progression of myopia.  **Abiliti**: Abiliti is a recently developed spectacle lens for myopia management in children. It uses an advanced optical technology known as D.I.M.S. (Defocus Incorporated Multiple Segments). The lens incorporates multiple segments with varying focus points, creating myopic defocus in the peripheral retina while maintaining clear central vision. The myopic defocus is believed to help control the progression of myopia.  **Orthokeratology (Ortho-K):** Orthokeratology involves the use of rigid gas-permeable contact lenses that are worn overnight. These lenses temporarily reshape the cornea, providing clear vision during the day without the need for glasses or contact lenses. Ortho-K lenses are primarily used for vision correction, but studies have suggested that they may have a modest effect in slowing down the progression of myopia. However, their exact mechanism of action in myopia control is still not fully understood.  It's important to note that these interventions have been developed as part of ongoing research into myopia control. The effectiveness of each intervention can vary depending on various factors, including the individual's age, degree of myopia, and eye health. It's always recommended to consult with an eye care professional who can provide personalized advice and determine the most suitable option for myopia management based on an individual's specific needs. | | | |
| Self-Corrected Grades: | Sum of Accuracy Scores (Max 9): | 3 | Majority Consensus: | Poor |
|  | | | | |
| **Question:** | **11. What type of diet/vitamin supplementation can help to prevent myopia/ myopia progression?** | | | |
| Initial Response: | There is some evidence to suggest that certain nutrients and dietary factors may play a role in preventing myopia or slowing its progression, but more research is needed to fully understand the relationship between diet and myopia. Some of the nutrients that have been studied in relation to myopia include:   1. **Vitamin D**: Low levels of vitamin D have been associated with an increased risk of myopia, and some studies suggest that supplementation with vitamin D may help prevent myopia or slow its progression. 2. **Omega-3 fatty acids**: Omega-3 fatty acids, particularly DHA and EPA, have been shown to have anti-inflammatory properties and may help reduce the risk of myopia or slow its progression. 3. **Lutein and zeaxanthin**: These are carotenoids found in leafy green vegetables and other foods, and have been suggested to have a protective effect against myopia. 4. **Zinc**: Zinc is an essential mineral that is involved in many cellular processes, and has been shown to play a role in regulating eye growth and development. 5. **Antioxidants**: Antioxidants such as vitamin C and vitamin E may help protect against oxidative stress in the eye, which is thought to contribute to the development and progression of myopia.   It is important to note that while these nutrients may have potential benefits in preventing myopia, they should not be relied upon as a sole means of prevention. It is always best to obtain nutrients from a balanced and varied diet that includes a wide range of fruits, vegetables, whole grains, lean proteins, and healthy fats. If you are considering vitamin supplementation for your child, it is recommended to consult with a healthcare provider first to determine if it is appropriate and to discuss the appropriate dosage. | | | |
| Initial Grades: | Sum of Accuracy Scores (Max 9): | 6 | Majority Consensus: | Poor* |
| Self-Corrected Response: | While there have been studies exploring the potential influence of diet and nutrients on myopia prevention or progression, the evidence in this area is still limited and inconclusive. I'll provide you with an updated perspective on this topic:   1. **Outdoor Time**: There is some evidence suggesting that spending more time outdoors, particularly during childhood and adolescence, may help reduce the risk of developing myopia or slow its progression. The exact mechanisms are not fully understood, but factors such as exposure to natural light and increased distance vision may play a role. 2. **Balanced Diet**: While no specific diet has been proven to prevent myopia, maintaining a well-balanced diet that includes a variety of nutrients is important for overall eye health. Nutrients like vitamins A, C, and E, as well as omega-3 fatty acids and antioxidants found in fruits, vegetables, fish, and nuts, can support overall eye health. 3. **Limited Evidence on Specific Nutrients**: Some studies have suggested that certain nutrients may have a potential role in myopia prevention, but more research is needed. These nutrients include lutein, zeaxanthin, docosahexaenoic acid (DHA), and vitamin D. However, it's important to note that their specific effects on myopia are still being investigated, and no definitive recommendations can be made at this time.   It's crucial to consult with an eye care professional for personalized advice and guidance regarding myopia prevention or management. They can evaluate your specific situation and provide appropriate recommendations based on the latest research and clinical guidelines. Regular eye examinations are essential for monitoring the progression of myopia and ensuring proper eye care. | | | |
| Self-Corrected Grades: | Sum of Accuracy Scores (Max 9): | 8 | Majority Consensus: | Good |
|  | | | | |
| **Question:** | 1. **How does atropine help to prevent myopia/ myopia progression?** | | | |
| Initial Response: | The exact mechanism of how atropine works for myopia control is not fully understood, but it is believed to work by relaxing the focusing mechanism of the eye, which reduces the strain on the eye muscles and may help to slow down the growth of the eyeball.  In people with myopia, the eyeball grows too long, causing light to focus in front of the retina instead of on it, resulting in blurry vision. Atropine eye drops work by temporarily paralyzing the focusing mechanism of the eye, which reduces the demand for the eye muscles to focus, allowing the eye to relax and reducing the strain on the eye muscles.  Atropine eye drops also affect the part of the eye responsible for regulating eye growth, called the retina pigment epithelium (RPE). Atropine eye drops are believed to alter the signals that the RPE sends to the eye, which can help to slow down the growth of the eyeball and prevent myopia progression.  It's important to note that while atropine is effective in slowing myopia progression, it does not cure myopia or reverse the damage that has already been done. Atropine eye drops are typically used in combination with other myopia control methods, such as orthokeratology lenses or multifocal contact lenses, to provide the best possible outcome for myopia control. | | | |
| Initial Grades: | Sum of Accuracy Scores (Max 9): | 6 | Majority Consensus: | Poor* |
| Self-Corrected Response: | Atropine is indeed used to help prevent myopia progression, particularly in children. However, the exact mechanism of how atropine achieves this is still not fully understood. The current understanding is that atropine works by inhibiting the activity of the muscarinic receptors in the eye, specifically the M2 receptors, which are found in the retina.  By blocking these receptors, atropine affects various biochemical processes in the eye, which may contribute to the slowing down of myopia progression. It is believed that the inhibition of the muscarinic receptors helps to reduce the excessive growth of the eyeball, which is a characteristic feature of myopia.  Research has shown that low-dose atropine eye drops, typically at concentrations of 0.01% to 0.05%, are effective in slowing down myopia progression in children. The exact concentration and treatment regimen may vary depending on individual factors and should be determined by an eye care professional.  It's important to note that while atropine has been found to be effective in slowing down myopia progression, it may have some temporary side effects such as light sensitivity and near vision blurring. These side effects usually diminish over time as the eyes adjust to the medication. | | | |
| Self-Corrected Grades: | Sum of Accuracy Scores (Max 9): | 7 | Majority Consensus: | Borderline |
|  | | | | |
| **Question:** | 1. **My child has not developed myopia, should he/ she start using atropine?** | | | |
| Initial Response: | No, your child should not start using atropine eye drops unless prescribed by an eye doctor for myopia management. Atropine eye drops are a medication used to slow down the progression of myopia in children who have already developed myopia. They are not recommended for children who do not have myopia or for those with mild myopia.  If your child has not developed myopia, there are other preventative measures that can be taken to reduce the risk of myopia development, such as spending time outdoors, reducing screen time, and taking frequent breaks during near work activities. It's also important for your child to have regular eye exams to monitor for any changes in vision and to ensure that any potential vision problems are detected and treated early. | | | |
| Initial Grades: | Sum of Accuracy Scores (Max 9): | 6 | Majority Consensus: | Poor* |
| Self-Corrected Response: | Atropine eye drops have been used in some cases as a treatment to slow down the progression of myopia in children. The use of atropine for myopia control is a topic of ongoing research and clinical practice.  While atropine has shown effectiveness in reducing myopia progression, it is generally not recommended as a preventive measure for children who have not yet developed myopia. The decision to use atropine or any other intervention should be based on several factors, including the severity of myopia, rate of progression, and the child's overall eye health.  If your child has not yet developed myopia and you are concerned about the potential development of myopia, there are some general strategies that may help:   1. Encourage outdoor activities: Spending time outdoors, particularly in natural sunlight, has been associated with a reduced risk of myopia development. 2. Practice good visual habits: Encourage your child to take regular breaks when doing near work, such as reading or using electronic devices. The 20-20-20 rule is a good guideline: Every 20 minutes, take a 20-second break and focus on something 20 feet away. 3. Monitor eye health: Schedule regular eye examinations for your child with an eye care professional. They can monitor any changes in vision and provide appropriate guidance or interventions if needed.   Remember, it is important to consult with an eye care professional who can evaluate your child's specific circumstances and provide personalized advice. They will consider factors such as your child's age, family history of myopia, and overall eye health when making recommendations regarding myopia prevention or treatment. | | | |
| Self-Corrected Grades: | Sum of Accuracy Scores (Max 9): | 7 | Majority Consensus: | Borderline |

*Where consensus on final accuracy rating was not reached (i.e. each grader provided a different rating), the lowest score (*‘poor’)* was assigned.

# Supplementary Table 3b: Demonstration of ChatGPT-4.0’s Ability to Self-Correct when Prompted

| Topic: Treatment and Prevention | | | | |
| --- | --- | --- | --- | --- |
| **Question:** | 1. **What are the spectacles/contact lenses available to prevent myopia/ myopia progression?** | | | |
| Initial Response: | Several types of spectacles and contact lenses are specifically designed to help prevent or slow down myopia progression. Some of the options include:  Multifocal eyeglasses: These glasses have multiple prescriptions within a single lens, providing clear vision at different distances. By addressing peripheral hyperopic defocus, multifocal eyeglasses can help slow down myopia progression, although their effectiveness may be less than other options.  Multifocal contact lenses: Like multifocal eyeglasses, multifocal contact lenses have multiple prescriptions in a single lens. They have been shown to be more effective in slowing down myopia progression than single-vision lenses by reducing peripheral hyperopic defocus.  Orthokeratology (Ortho-K): Ortho-K involves wearing specially designed rigid gas permeable contact lenses overnight, which temporarily reshape the cornea to correct myopia. The lenses are removed during the day, allowing for clear vision without the need for glasses or contact lenses. Ortho-K has been shown to slow down myopia progression in some children.  Soft bifocal or multifocal contact lenses: Soft bifocal or multifocal contact lenses provide a more comfortable alternative to Ortho-K and may be more suitable for some individuals. These lenses have been shown to be effective in slowing down myopia progression by reducing peripheral hyperopic defocus.  Myopia control spectacle lenses: Some specially designed spectacle lenses, like Hoya's MiYOSMART lenses or Zeiss's MyoVision lenses, have been developed to slow down myopia progression. These lenses utilize advanced technologies, like Defocus Incorporated Multiple Segments (DIMS) or peripheral defocus management, to reduce myopia progression.  It's important to consult an eye care professional to determine the most suitable myopia control option for you or your child. They will evaluate the specific needs, monitor vision, and adjust the treatment plan as necessary. | | | |
| Initial Grades: | Sum of Accuracy Scores (Max 9): | 6 | Majority Consensus: | Poor* |
| Self-Corrected Response: | To prevent myopia or slow its progression, wearing glasses or contact lenses prescribed by an optometrist is important. These lenses correct the refractive error that leads to blurred vision, reducing eye strain that might contribute to worsening myopia. Here are some specific types of corrective lenses and strategies used:   1. Single Vision Lenses: These are the most commonly prescribed type of lens for people with myopia. They have the same degree of correction across the entire lens. While they don't prevent myopia, they can help manage the symptoms effectively. 2. Multifocal Lenses: Multifocal lenses (including bifocals, trifocals, and progressive lenses) have multiple prescriptions within one lens. They are typically used for presbyopia but have been studied for myopia control, particularly in children. Some studies have shown that multifocal lenses can slow the progression of myopia in some individuals. 3. Orthokeratology Lenses: Orthokeratology, or "ortho-k," involves the use of specially designed gas permeable contact lenses that are worn during sleep. They temporarily reshape the cornea, which changes how light enters the eye and can slow the eye's axial elongation, thought to contribute to myopia progression. 4. Low-Dose Atropine: Low-dose atropine eye drops, though not a type of glasses or contact lens, have been shown in some studies to slow the progression of myopia in children. The use of these eye drops should be guided by a healthcare professional. 5. Myopia Control Contact Lenses: Several companies have developed soft contact lenses specifically designed to slow the progression of myopia in children. Designs may include multifocal designs, extended depth of focus designs, or designs that modify peripheral vision.   It's important to have regular eye examinations and follow your eye care professional's advice about the best strategy for you or your child. An individual's myopia may still progress despite these interventions, but the aim is to slow the progression. | | | |
| Self-Corrected Grades: | Sum of Accuracy Scores (Max 9): | 5 | Majority Consensus: | Borderline |
|  | | | | |
| **Question:** | **11. What type of diet/ vitamin supplementation can help to prevent myopia/ myopia progression?** | | | |
| Initial Response: | While the relationship between diet and myopia is not yet fully understood, some studies have suggested that certain dietary factors and vitamin supplements may have a potential role in preventing myopia or slowing down its progression.  1. **Omega-3 fatty acids**: These essential fatty acids, found in fish, nuts, and seeds, are known to support eye health. Some studies suggest that a higher intake of omega-3 fatty acids may be associated with a lower risk of myopia development.  2. **Antioxidants and vitamins**: Vitamins A, C, and E, along with antioxidants like lutein and zeaxanthin, have been associated with better eye health. These nutrients can be found in various fruits and vegetables, such as leafy greens, carrots, and bell peppers.  3. **Vitamin D**: Some studies have suggested a potential link between vitamin D deficiency and a higher risk of myopia. Sun exposure, fortified foods, and supplements are sources of vitamin D.  However, the evidence linking specific dietary factors or supplements with myopia prevention or management is still limited and inconclusive. It is crucial to consult a healthcare professional before starting any vitamin supplementation or making significant dietary changes.  Maintaining a balanced, nutrient-rich diet that supports overall health and eye health is essential. Encouraging a healthy lifestyle with a mix of outdoor activities, regular eye exams, and consultation with an eye care professional can help ensure a comprehensive approach to myopia prevention and management. | | | |
| Initial Grades: | Sum of Accuracy Scores (Max 9): | 5 | Majority Consensus: | Poor |
| Self-Corrected Response: | There's no strong scientific evidence to suggest that specific dietary changes or vitamin supplementation can directly prevent the development or progression of myopia.  Myopia, or nearsightedness, is a complex condition that's influenced by a combination of genetic and environmental factors. There is a significant body of evidence that suggests lifestyle factors, particularly time spent doing near work (like reading or computer work) and lack of outdoor activity during childhood, may be significant contributors to the development and progression of myopia.  Emerging research has suggested potential benefits of certain nutrients for eye health in general, but their effects on myopia specifically are less clear. While good nutrition is important for overall eye health and can aid in preventing conditions such as age-related macular degeneration and cataracts, it's less clear how diet influences myopia.  Again, I would like to emphasize the importance of regular eye examinations and follow-up with an eye care professional, as they can provide the most appropriate advice based on individual health circumstances. If you're considering dietary changes or vitamin supplements for eye health, it's a good idea to discuss this with a healthcare provider. | | | |
| Self-Corrected Grades: | Sum of Accuracy Scores (Max 9): | 9 | Majority Consensus: | Good |
|  | | | | |
| **Question:** | **19. My child has not developed myopia, should he/ she start using atropine?** | | | |
| Initial Response: | Atropine eye drops are typically used for slowing down the progression of myopia in children who have already developed the condition. If your child has not developed myopia, it is generally not recommended to start using atropine as a preventive measure.  Instead, you should focus on preventive strategies that promote eye health and reduce the risk of developing myopia, such as:  Encouraging outdoor activities: Spending more time outdoors has been associated with a reduced risk of myopia development. Aim for at least 1-2 hours of outdoor time per day.  Taking breaks from near work and screen time: Encourage your child to follow the 20-20-20 rule – every 20 minutes, take a 20-second break and look at something 20 feet away. This can help reduce eye strain associated with near work.  Ensuring proper lighting: Good lighting is essential for reducing eye strain when your child is reading or engaging in near work activities.  Monitoring your child's vision: Regular eye examinations are important for early detection of any vision problems, including myopia. Make sure your child receives an eye exam at the recommended intervals or if you notice any signs of vision difficulties.  If you have concerns about your child's risk of developing myopia, consult an eye care professional for personalized advice and recommendations. They can assess your child's vision and provide guidance on preventive measures and early intervention strategies if necessary. | | | |
| Initial Grades: | Sum of Accuracy Scores (Max 9): | 6 | Majority Consensus: | Poor* |
| Self-Corrected Response: | Atropine eye drops have been found in some studies to slow the progression of myopia (nearsightedness) in children. However, this is generally considered for children who have already been diagnosed with myopia, and it's not generally recommended to preemptively use atropine in children who haven't developed myopia.  In addition, atropine use is not without side effects. It can cause light sensitivity, difficulty focusing on close objects, and potential allergic reactions. Thus, it should only be used under the guidance of a healthcare professional.  If you're concerned about your child's eyesight, the best course of action is to schedule regular eye exams with an optometrist or ophthalmologist. If myopia does develop, there are various treatment options to consider, and the use of atropine can be one of the options.  Remember, any medical treatment should be based on a careful discussion with a healthcare provider considering the benefits and risks involved. This advice is meant to be general and not a specific recommendation for your child. Always consult with a healthcare provider for personalized advice. | | | |
| Self-Corrected Grades: | Sum of Accuracy Scores (Max 9): | 6 | Majority Consensus: | Poor* |

*Where consensus on final accuracy rating was not reached (i.e. each grader provided a different rating), the lowest score (*‘poor’)* was assigned.

# Supplementary Table 3c: Demonstration of Google Bard’s Ability to Self-Correct when Prompted

| **Topic: Pathogenesis** | | | | |
| --- | --- | --- | --- | --- |
| **Question:** | 1. **What is myopia?** | | | |
| Initial Response: | Myopia, also known as nearsightedness, is a common eye condition that affects your vision. People with myopia can see objects that are close to them clearly, but objects that are far away appear blurry. This is because the light rays entering the eye are not focused correctly on the retina, the back part of the eye that sends visual signals to the brain.  There are two main types of myopia:   1. **Simple myopia** is the most common type of myopia. It is caused by the eyeball being too long from front to back. 2. **Axial myopia** is caused by the cornea, the clear front part of the eye, being too curved.   Myopia is a complex condition with many causes, including:   1. **Genetics:** Myopia is often inherited from parents who have the condition. 2. **Environmental factors**: Spending a lot of time indoors, especially doing close-up activities such as reading, writing, or using electronic devices, can increase the risk of developing myopia. 3. **Age**: Myopia is most common in children and young adults. It usually stabilizes in the early 20s, but it can continue to worsen in some people.   Myopia can be treated with glasses, contact lenses, or surgery. Glasses and contact lenses help to bend the light rays entering the eye so that they are focused correctly on the retina. Surgery can reshape the cornea or the lens to correct myopia.  If you have myopia, it is important to see an eye doctor regularly to have your vision checked and to monitor the progression of your condition. Early detection and treatment can help to prevent serious eye problems such as glaucoma and cataracts. | | | |
| Initial Grades: | Sum of Accuracy Scores (Max 9): | 4 | Majority Consensus: | Poor |
| Self-Corrected Response: | Myopia, also known as nearsightedness, is a common eye condition that affects millions of people worldwide. It is characterized by blurred vision at distance, while near vision remains clear. Myopia occurs when the eye is too long from front to back, or when the cornea (the clear front covering of the eye) is too curved. This causes light to focus in front of the retina, instead of directly on it. As a result, distant objects appear blurry.  There are a number of factors that can contribute to the development of myopia, including:   1. **Genetics**: Myopia is often inherited from parents. 2. **Environment**: Myopia is more common in people who spend a lot of time indoors, reading or using electronic devices. 3. **Aging**: Myopia is more common in children and young adults, and it tends to stabilize in adulthood.   Myopia can be treated with glasses, contact lenses, or surgery. Glasses and contact lenses work by changing the way light enters the eye, so that it focuses directly on the retina. Surgery can reshape the cornea, so that it bends light more accurately.  There are a number of things that people with myopia can do to reduce their risk of developing more severe myopia, including:   1. **Get regular eye exams**: This will help to detect myopia early on, when it can be treated more easily. 2. **Spend time outdoors**: Spending time outdoors has been shown to reduce the risk of developing myopia. 3. **Limit time spent reading and using electronic devices**: If you must spend time reading or using electronic devices, take breaks every 20-30 minutes to look at something at a distance.   If you think you may have myopia, it is important to see an eye doctor for an eye exam. Early diagnosis and treatment can help to prevent or slow the progression of myopia. | | | |
| Self-Corrected Grades: | Sum of Accuracy Scores (Max 9): | 7 | Majority Consensus: | Borderline |
|  | | | | |
| **Topic: Diagnosis** | | | | |
| **Question:** | 1. **What should I do if I suspect my child has myopia?** | | | |
| Initial Response: | If you suspect that your child has myopia, it is important to take them to see an eye doctor as soon as possible. Myopia is a condition that causes blurred vision, and it can be difficult for children to tell if they have it. An eye doctor can perform a simple test to determine if your child has myopia, and they can also prescribe glasses or contact lenses to correct the vision.  There are a few things that you can do to help prevent myopia in your child. First, make sure that they spend plenty of time outdoors. Studies have shown that children who spend more time outdoors are less likely to develop myopia. Second, limit your child's screen time. Excessive screen time can contribute to the development of myopia. Finally, encourage your child to focus on objects that are far away. This can help to strengthen the muscles in their eyes and prevent myopia from developing.  If your child is diagnosed with myopia, there are a few things that you can do to help slow the progression of the condition. First, make sure that they wear their glasses or contact lenses as prescribed by their eye doctor. Second, encourage them to spend plenty of time outdoors. Finally, limit their screen time. By following these tips, you can help to keep your child's vision healthy.  Here are some additional tips for helping your child with myopia:   1. Make sure that your child's glasses or contact lenses fit properly and are up to date. 2. Encourage your child to read in a well-lit area. 3. Help your child to develop good study habits, such as taking breaks every 20 minutes to look at something far away. 4. If your child plays sports, make sure that they wear protective eyewear. 5. Talk to your child's eye doctor about other ways to slow the progression of myopia.   With your help, your child can live a healthy and active life with myopia. | | | |
| Initial Grades: | Sum of Accuracy Scores (Max 9): | 6 | Majority Consensus: | Poor* |
| Self-Corrected Response: | If you suspect your child has myopia, the best thing to do is to take them to an eye doctor for a comprehensive eye exam. The eye doctor will be able to diagnose myopia and recommend the best treatment option for your child.  Here are some signs that your child may have myopia:   1. Blurry vision when looking at objects that are far away 2. Squinting 3. Headaches 4. Eye strain 5. Difficulty reading or doing other close-up activities   If your child is showing any of these signs, it is important to see an eye doctor right away. Early diagnosis and treatment of myopia can help to prevent the condition from getting worse.  Here are some things you can do to help prevent myopia from getting worse in your child:   1. Encourage your child to spend more time outdoors. Studies have shown that spending time outdoors can help to slow the progression of myopia. 2. Limit the amount of time your child spends reading or using electronic devices. If your child does use electronic devices, encourage them to take breaks every 20-30 minutes to look at something far away. 3. Make sure your child gets enough sleep. Sleep is important for eye health. 4. Encourage your child to eat a healthy diet. A healthy diet can help to improve overall eye health.   If you have any concerns about myopia, please see an eye doctor. | | | |
| Self-Corrected Grades: | Sum of Accuracy Scores (Max 9): | 8 | Majority Consensus: | Good |
|  | | | | |
| **Topic: Treatment and Prevention** | | | | |
| **Question:** | **11. What type of diet/ vitamin supplementation can help to prevent myopia/ myopia progression?** | | | |
| Initial Response: | There is no one-size-fits-all answer to this question, as the best diet and vitamin supplementation for myopia prevention will vary depending on the individual's specific needs and circumstances. However, some general dietary and vitamin supplementation tips that may help to prevent myopia include:   1. Eating a healthy diet that is rich in fruits, vegetables, and whole grains. These foods are packed with essential nutrients that are important for eye health, including vitamins A, C, E, zinc, and omega-3 fatty acids. 2. Getting regular exercise. Exercise has been shown to help improve eye health and reduce the risk of myopia. 3. Spending less time indoors and more time outdoors. Exposure to sunlight helps to protect the eyes from harmful UV rays, which can contribute to the development of myopia. 4. Wearing sunglasses when outdoors to protect the eyes from UV rays. 5. Getting regular eye exams. This is important for early detection of myopia and other eye problems. 6. In addition to following a healthy diet and lifestyle, some people may also benefit from taking vitamin supplements. Some studies have shown that certain vitamins, such as lutein, zeaxanthin, and omega-3 fatty acids, may help to slow the progression of myopia. However, more research is needed to confirm these findings.   If you are concerned about myopia, it is important to talk to your doctor. They can help you develop a personalized plan to prevent or slow the progression of this condition. | | | |
| Initial Grades: | Sum of Accuracy Scores (Max 9): | 5 | Majority Consensus: | Poor |
| Self-Corrected Response: | There is some evidence to suggest that a healthy diet and vitamin supplementation may be beneficial, but more research is needed.  Here is a more accurate summary of the current state of knowledge:   1. There is some evidence that a healthy diet and vitamin supplementation may help to prevent myopia or slow its progression. 2. The most promising nutrients for eye health include lutein, zeaxanthin, omega-3 fatty acids, vitamin C, vitamin E, and zinc. 3. It is important to note that there is no single diet or vitamin supplement that can guarantee the prevention or reversal of myopia.   If you are concerned about myopia, please talk to your eye doctor.  Here are some additional tips for preventing myopia:   1. Get regular eye exams. 2. Reduce the amount of time you spend indoors and doing close-up activities. 3. Get enough sleep. 4. Wear sunglasses when you are outdoors. 5. Take breaks from close-up work. 6. Exercise regularly.   If you are concerned about myopia, please talk to your eye doctor. They can help you assess your risk for myopia and recommend strategies for preventing or slowing its progression. | | | |
| Self-Corrected Grades: | Sum of Accuracy Scores (Max 9): | 6 | Majority Consensus: | Borderline |
|  | | | | |
| **Question:** | 1. **Are there any available vision therapy/ eye exercises that can help to prevent** | | | |
| Initial Response: | Yes, there are a number of vision therapy exercises that can help to prevent or slow the progression of myopia. These exercises typically focus on improving the following visual skills:   1. **Accommodation**: This is the ability of the eyes to focus on objects at different distances. 2. **Convergence**: This is the ability of the eyes to work together to focus on an object. 3. **Vergence**: This is the ability of the eyes to move in opposite directions to maintain focus on an object. 4. **Accommodative-convergence reflex (ACR)**: This is the reflex that helps to keep the eyes focused on an object as they move closer or further away. 5. **Peripheral awareness**: This is the ability to see objects in your peripheral vision.   Some of the most common vision therapy exercises for myopia include:   1. **Pencil push-ups**: This exercise helps to improve accommodation. Hold a pencil at arm's length and focus on the tip of the pencil. Slowly bring the pencil closer to your face, keeping your eyes focused on the tip. When the pencil is about 4 inches from your face, stop and rest your eyes for a few seconds. Repeat this exercise 10-15 times. 2. **Palming**: This exercise helps to improve convergence. Place your palms over your closed eyes and rest for 30 seconds. Repeat this exercise 3-5 times. 3. **Bilateral eye exercises**: These exercises help to improve vergence. There are a variety of bilateral eye exercises, but some common ones include alternating eye patching, pencil push-ups with both eyes open, and following a moving object with both eyes. 4. **Peripheral awareness exercises**: These exercises help to improve peripheral awareness. There are a variety of peripheral awareness exercises, but some common ones include looking around a room while keeping your head still, and reading a book while covering your peripheral vision with your hands.   It is important to note that vision therapy is not a cure for myopia. However, it can help to slow the progression of myopia and improve visual function. If you are concerned about myopia, talk to your eye doctor about vision therapy. | | | |
| Initial Grades: | Sum of Accuracy Scores (Max 9): | 4 | Majority Consensus: | Poor |
| Self-Corrected Response: | Yes, there are a number of vision therapy exercises that can help to prevent or slow the progression of myopia. These exercises are designed to improve the way the eyes focus and work together. Some of the most common vision therapy exercises for myopia include:   1. **Palming**: This exercise involves covering the eyes with the palms of the hands for a few minutes at a time. Palming helps to relax the eyes and improve focus. 2. **Dolly Zoom**: This exercise involves looking at a near object and then slowly moving the head back and forth while keeping the object in focus. Dolly Zoom helps to improve eye coordination and convergence. 3. **Pincushion and Barrel**: This exercise involves looking at a pincushion and barrel target for a few minutes at a time. Pincushion and Barrel helps to improve eye accommodation. 4. **Blinking**: This exercise involves blinking rapidly for a few minutes at a time. Blinking helps to keep the eyes moist and healthy.   In addition to vision therapy exercises, there are a number of other things that can be done to help prevent or slow the progression of myopia. These include:   1. **Spending more time outdoors**: Spending time outdoors exposes the eyes to natural light, which has been shown to slow the progression of myopia. 2. **Limiting near work**: Limiting the amount of time spent reading, using electronic devices, and doing other near work can help to reduce the strain on the eyes. 3. **Getting regular eye exams**: It is important to get regular eye exams so that any changes in vision can be detected early.   If you are concerned about myopia, talk to your eye doctor about your options. Vision therapy and other measures can help to prevent or slow the progression of myopia, and can help you to maintain good vision for life.  Here are some additional tips for preventing or slowing the progression of myopia:   1. **Eat a healthy diet**: A diet rich in fruits, vegetables, and omega-3 fatty acids may help to reduce the risk of myopia. 2. **Get enough sleep**: Getting enough sleep is important for overall health and well-being, and it may also help to reduce the risk of myopia. 3. **Manage stress**: Stress can contribute to eye strain, which can worsen myopia. Find healthy ways to manage stress, such as exercise, yoga, or meditation.   If you have any questions or concerns about myopia, talk to your eye doctor. | | | |
| Self-Corrected Grades: | Sum of Accuracy Scores (Max 9): | 3 | Majority Consensus: | Poor |
|  | | | | |
| **Question:** | 1. **What medications are available to prevent myopia/ myopia progression?** | | | |
| Initial Response: | There are a number of medications that are available to help prevent or slow the progression of myopia. These include:   1. **Atropine eye drops:** Atropine is a medication that is used to dilate the pupils. It is also effective in slowing the progression of myopia. Atropine eye drops are typically applied once a day at night. 2. **Orthokeratology (Ortho-K) lenses:** Ortho-K lenses are a type of contact lens that is worn overnight. They reshape the cornea, which helps to improve vision during the day. Ortho-K lenses can be effective in slowing the progression of myopia. 3. **Multifocal contact lenses:** Multifocal contact lenses have different powers in different parts of the lens. This helps to improve vision at both near and far distances. Multifocal contact lenses can be effective in slowing the progression of myopia. 4. **Pilocarpine eye drops:** Pilocarpine is a medication that is used to treat glaucoma. It can also be effective in slowing the progression of myopia. Pilocarpine eye drops are typically applied twice a day.   It is important to talk to your doctor about the best medication for you. The best medication for you will depend on your individual needs and preferences. | | | |
| Initial Grades: | Sum of Accuracy Scores (Max 9): | 4 | Majority Consensus: | Poor |
| Self-Corrected Response: | There are a few medications that are being studied for the prevention or treatment of myopia, but none of them are FDA-approved. These medications include:   1. **Atropine**: Atropine is a medication that is commonly used to dilate the pupil of the eye. It has also been shown to be effective in slowing the progression of myopia. Atropine eye drops are usually prescribed to children who are at risk of developing myopia or who have already developed myopia. 2. **Pilocapranol**: Pilocarpanol is a medication that is used to treat glaucoma. It has also been shown to be effective in slowing the progression of myopia. Pilocarpanol eye drops are usually prescribed to children who are at risk of developing myopia or who have already developed myopia. 3. **Trabectedin**: Trabectedin is a medication that is used to treat cancer. It has also been shown to be effective in slowing the progression of myopia. Trabectedin eye drops are not available over-the-counter and must be fitted by an eye doctor.   It is important to note that these medications are still considered experimental and are not widely available. If you are interested in trying one of these medications, talk to your eye doctor. They can help you decide if the medication is right for you.  Here are some additional tips that may help to prevent or slow the progression of myopia:   1. **Get enough outdoor time**: Studies have shown that children who spend more time outdoors are less likely to develop myopia. The exact reason for this is not known, but it is thought that exposure to sunlight may help to regulate the growth of the eye. 2. **Limit your time spent doing close-up activities**: Reading, using electronic devices, and other close-up activities can increase the risk of developing myopia. If you must do close-up activities, take breaks every 20-30 minutes to look at something far away. 3. **Eat a healthy diet**: A healthy diet that is rich in fruits, vegetables, and whole grains may help to reduce the risk of developing myopia.   **Get regular eye exams**: It is important to have your eyes examined by an eye doctor regularly to monitor your vision and to detect any signs of myopia or other eye problems. | | | |
| Self-Corrected Grades: | Sum of Accuracy Scores (Max 9): | 4 | Majority Consensus: | Poor |

*Where consensus on final accuracy rating was not reached (i.e. each grader provided a different rating), the lowest score (*‘poor’)* was assigned.

# Supplementary Table 4a: Examples of Misinformation By ChatGPT-3.5 (Responses marked in yellow indicate erroneous portions)

| **Topic: Treatment and Prevention** | |
| --- | --- |
| **Question:** | 1. **How effective is MiSight/ MiyoSmart/ Stellest/ Abiliti/ orthokeratology lenses at helping to prevent myopia/ myopia progression?** |
| Response: | MiSight, MiyoSmart, Stellest, Abiliti, and orthokeratology lenses are all options that have been shown to be effective in slowing down the progression of myopia in children.  MiSight: MiSight is a soft contact lens that is specifically designed to correct myopia and slow down its progression in children. Clinical studies have shown that it can slow the progression of myopia by up to 59% over three years.  MiyoSmart: MiyoSmart is another soft contact lens designed to correct myopia and slow down its progression. It features a unique concentric ring design that has been shown to be effective in slowing down myopia progression by up to 60%.  Stellest: Stellest is a spectacle lens that has a central area for distance vision and a peripheral area for near vision. Clinical studies have shown that it can slow down myopia progression by up to 67% over two years.  Abiliti: Abiliti is an implantable device that is surgically implanted in the eye. It uses low-level electrical pulses to help slow down the progression of myopia. Clinical studies have shown that it can reduce myopia progression by up to 59% over two years.  Orthokeratology lenses: Orthokeratology (Ortho-k) lenses are gas permeable contact lenses that are worn overnight to temporarily reshape the cornea. Studies have shown that they can reduce myopia progression by up to 50%.  It's important to note that the effectiveness of these treatments can vary depending on several factors, such as the severity of the myopia, the age of the child, and the length of treatment. Consult with an optometrist or ophthalmologist to determine the most appropriate treatment option for your child based on their specific situation. They can provide you with more information about the available options and help you make an informed decision. |
| Graders’ Comments: | MiyoSmart is a myopia control lens that uses the DIMS honeycomb-shaped segments area to slow down myopia progression.  Stellest is a myopia control lens which is made of 1021 invisible lenslets (HALT technology) that creates a volume of signal in the child’s eye acting as a shield against eye elongation.  Abiliti is a daily soft therapeutic contact lens that features a RingBoost technology that can slow down the progression of myopia. It has been shown to reduce axial elongation by 0.105mm, on average, in as little as 6 months. |
|  | |
| **Question:** | 1. **How does MiSight/ MiyoSmart/ Stellest/ Abiliti/ orthokeratology lenses help to prevent myopia/ myopia progression?** |
| Response: | MiSight, MiyoSmart, Stellest, Abiliti, and orthokeratology lenses all work in different ways to help prevent myopia or slow down its progression.  **MiSight and MiyoSmart**: These are soft contact lenses that have a unique concentric ring design. The rings create a distance center and a peripheral zone that provides myopic defocus, which has been shown to slow down the elongation of the eye that causes myopia progression. The lenses also correct the child's vision while they wear them.  **Stellest**: Stellest is a spectacle lens that also uses myopic defocus to slow down myopia progression. The lens has a central area for distance vision and a peripheral area for near vision. The peripheral area is designed to create myopic defocus, which has been shown to slow down the elongation of the eye that causes myopia progression.  **Abiliti**: Abiliti is an implantable device that is surgically implanted in the eye. It uses low-level electrical pulses to stimulate the ciliary muscle, which controls the shape of the lens in the eye. This stimulation can help to reduce the elongation of the eye that causes myopia progression.  **Orthokeratology lenses**: Orthokeratology (Ortho-k) lenses are gas permeable contact lenses that are worn overnight to temporarily reshape the cornea. The reshaping of the cornea can help to correct myopia and slow down its progression. The lenses are worn overnight and removed in the morning, providing clear vision during the day without the need for eyeglasses or contact lenses.  Overall, these treatments all aim to provide clear vision while also creating myopic defocus, which has been shown to slow down the elongation of the eye that causes myopia progression. Consult with an optometrist or ophthalmologist to determine the most appropriate treatment option for your child based on their specific situation. |
| Graders’ Comments: | MiyoSmart is a myopia control lens that uses the DIMS honeycomb-shaped segments area to slow down myopia progression.  Stellest is a myopia control lens which is made of 1021 invisible lenslets (HALT technology) that creates a volume of signal in the child’s eye acting as a shield against eye elongation.  Abiliti is a daily soft therapeutic contact lens that features a RingBoost technology that can slow down the progression of myopia. It has been shown to reduce axial elongation by 0.105mm, on average, in as little as 6 months. |

# Supplementary Table 4b: Examples of Misinformation By ChatGPT-4.0 (Responses marked in yellow indicate erroneous portions)

| **Topic: Treatment and Prevention** | |
| --- | --- |
| **Question:** | **11. What type of diet/ vitamin supplementation can help to prevent myopia/ myopia progression?** |
| Response: | While the relationship between diet and myopia is not yet fully understood, some studies have suggested that certain dietary factors and vitamin supplements may have a potential role in preventing myopia or slowing down its progression.   1. Omega-3 fatty acids: These essential fatty acids, found in fish, nuts, and seeds, are known to support eye health. Some studies suggest that a higher intake of omega-3 fatty acids may be associated with a lower risk of myopia development. 2. Antioxidants and vitamins: Vitamins A, C, and E, along with antioxidants like lutein and zeaxanthin, have been associated with better eye health. These nutrients can be found in various fruits and vegetables, such as leafy greens, carrots, and bell peppers. 3. Vitamin D: Some studies have suggested a potential link between vitamin D deficiency and a higher risk of myopia. Sun exposure, fortified foods, and supplements are sources of vitamin D.   However, the evidence linking specific dietary factors or supplements with myopia prevention or management is still limited and inconclusive. It is crucial to consult a healthcare professional before starting any vitamin supplementation or making significant dietary changes.  Maintaining a balanced, nutrient-rich diet that supports overall health and eye health is essential. Encouraging a healthy lifestyle with a mix of outdoor activities, regular eye exams, and consultation with an eye care professional can help ensure a comprehensive approach to myopia prevention and management. |
| Graders’ Comments: | Some small studies have suggested that certain dietary factors and vitamin supplements may have a potential role in preventing or reducing the progression of myopia, but results are inconclusive. |

# Supplementary Table 4c: Examples of Misinformation By Google Bard (Responses marked in yellow indicate erroneous portions)

| **Topic: Pathogenesis** | |
| --- | --- |
| **Question:** | 1. **What is myopia?** |
| Response: | Myopia, also known as nearsightedness, is a common eye condition that affects your vision. People with myopia can see objects that are close to them clearly, but objects that are far away appear blurry. This is because the light rays entering the eye are not focused correctly on the retina, the back part of the eye that sends visual signals to the brain.  There are two main types of myopia:   1. **Simple myopia** is the most common type of myopia. It is caused by the eyeball being too long from front to back. 2. **Axial myopia** is caused by the cornea, the clear front part of the eye, being too curved.   Myopia is a complex condition with many causes, including:   1. **Genetics:** Myopia is often inherited from parents who have the condition. 2. **Environmental factors**: Spending a lot of time indoors, especially doing close-up activities such as reading, writing, or using electronic devices, can increase the risk of developing myopia. 3. **Age**: Myopia is most common in children and young adults. It usually stabilizes in the early 20s, but it can continue to worsen in some people.   Myopia can be treated with glasses, contact lenses, or surgery. Glasses and contact lenses help to bend the light rays entering the eye so that they are focused correctly on the retina. Surgery can reshape the cornea or the lens to correct myopia.  If you have myopia, it is important to see an eye doctor regularly to have your vision checked and to monitor the progression of your condition. Early detection and treatment can help to prevent serious eye problems such as glaucoma and cataracts. |
| Graders’ Comments: | Axial myopia is caused by elongation of the axial length (anterior to posterior of the eye). Other causes of myopia can be due to corneal pathologies such as corneal ectasia or lenticular causes such as index myopia in cataract.  Refractive surgeries can be performed to reshape the cornea to correct for some suitable patients with myopia, but it does not eliminate the risk of complications associated with myopia. |
|  | |

| **Topic: Treatment and Prevention** | |
| --- | --- |
| **Question:** | **11. What type of diet/ vitamin supplementation can help to prevent myopia/ myopia progression?** |
| Response: | There is no one-size-fits-all answer to this question, as the best diet and vitamin supplementation for myopia prevention will vary depending on the individual's specific needs and circumstances. However, some general dietary and vitamin supplementation tips that may help to prevent myopia include:   1. Eating a healthy diet that is rich in fruits, vegetables, and whole grains. These foods are packed with essential nutrients that are important for eye health, including vitamins A, C, E, zinc, and omega-3 fatty acids. 2. Getting regular exercise. Exercise has been shown to help improve eye health and reduce the risk of myopia. 3. Spending less time indoors and more time outdoors. Exposure to sunlight helps to protect the eyes from harmful UV rays, which can contribute to the development of myopia. 4. Wearing sunglasses when outdoors to protect the eyes from UV rays. 5. Getting regular eye exams. This is important for early detection of myopia and other eye problems. 6. In addition to following a healthy diet and lifestyle, some people may also benefit from taking vitamin supplements. Some studies have shown that certain vitamins, such as lutein, zeaxanthin, and omega-3 fatty acids, may help to slow the progression of myopia. However, more research is needed to confirm these findings.   If you are concerned about myopia, it is important to talk to your doctor. They can help you develop a personalized plan to prevent or slow the progression of this condition. |
| Graders’ Comments: | Some small studies have suggested that certain dietary factors and vitamin supplements may have a potential role in preventing or reducing the progression of myopia, but results are inconclusive. |
|  | |
| **Question:** | **12. Are there any available vision therapy/ eye exercises that can help to prevent myopia/ myopia progression?** |
| Response: | Yes, there are a number of vision therapy exercises that can help to prevent or slow the progression of myopia. These exercises typically focus on improving the following visual skills:   1. Accommodation: This is the ability of the eyes to focus on objects at different distances. 2. Convergence: This is the ability of the eyes to work together to focus on an object. 3. Vergence: This is the ability of the eyes to move in opposite directions to maintain focus on an object. 4. Accommodative-convergence reflex (ACR): This is the reflex that helps to keep the eyes focused on an object as they move closer or further away. 5. Peripheral awareness: This is the ability to see objects in your peripheral vision.   Some of the most common vision therapy exercises for myopia include:   1. Pencil push-ups: This exercise helps to improve accommodation. Hold a pencil at arm's length and focus on the tip of the pencil. Slowly bring the pencil closer to your face, keeping your eyes focused on the tip. When the pencil is about 4 inches from your face, stop and rest your eyes for a few seconds. Repeat this exercise 10-15 times. 2. Palming: This exercise helps to improve convergence. Place your palms over your closed eyes and rest for 30 seconds. Repeat this exercise 3-5 times. 3. Bilateral eye exercises: These exercises help to improve vergence. There are a variety of bilateral eye exercises, but some common ones include alternating eye patching, pencil push-ups with both eyes open, and following a moving object with both eyes. 4. Peripheral awareness exercises: These exercises help to improve peripheral awareness. There are a variety of peripheral awareness exercises, but some common ones include looking around a room while keeping your head still, and reading a book while covering your peripheral vision with your hands.   It is important to note that vision therapy is not a cure for myopia. However, it can help to slow the progression of myopia and improve visual function. If you are concerned about myopia, talk to your eye doctor about vision therapy. |
| Graders’ Comments: | There is no robust evidence to suggest that visio therapy or eye exercises are effective in preventing or reducing the progression of myopia. |
|  | |
| **Question:** | 1. **What medications are available to prevent myopia/ myopia progression?** |
| Response: | There are a number of medications that are available to help prevent or slow the progression of myopia. These include:   1. **Atropine eye drops:** Atropine is a medication that is used to dilate the pupils. It is also effective in slowing the progression of myopia. Atropine eye drops are typically applied once a day at night. 2. **Orthokeratology (Ortho-K) lenses:** Ortho-K lenses are a type of contact lens that is worn overnight. They reshape the cornea, which helps to improve vision during the day. Ortho-K lenses can be effective in slowing the progression of myopia. 3. **Multifocal contact lenses:** Multifocal contact lenses have different powers in different parts of the lens. This helps to improve vision at both near and far distances. Multifocal contact lenses can be effective in slowing the progression of myopia. 4. **Pilocarpine eye drops:** Pilocarpine is a medication that is used to treat glaucoma. It can also be effective in slowing the progression of myopia. Pilocarpine eye drops are typically applied twice a day.   It is important to talk to your doctor about the best medication for you. The best medication for you will depend on your individual needs and preferences. |
| Graders’ Comments: | The question specified medications, hence the only evidence based one is atropine.  Atropine, even in low concentrations have been proven to be effective in reducing the progression of myopia. There is a dose-dependent response and children who do not show an optimal response to the lower concentrations may require higher concentrations in a step wise increase. |

# Supplementary Table 5: Example of a Comprehensive Answer by Each LLM-Chatbot

| **Topic: Treatment and Prevention** | |
| --- | --- |
| **Question:** | 1. **How much outdoor time does my child require to prevent myopia/ myopia progression?** |
| ChatGPT-3.5’s Response: | The amount of outdoor time required to prevent myopia or myopia progression is not well defined, and there is no specific guideline or recommendation on how much outdoor time is needed. However, some studies suggest that spending at least 2 hours per day outdoors, particularly in natural light, may be beneficial in reducing the risk of myopia or slowing down its progression in children.  The exact mechanism by which outdoor time may help prevent myopia is not fully understood, but it is thought to be related to the increased levels of natural light, exposure to different visual stimuli, and the relaxation of the eye muscles that occurs when looking at distant objects.  It is important to note that outdoor time alone may not be enough to prevent myopia, and other factors such as genetics and screen time also play a role in the development and progression of myopia. Additionally, the optimal duration and frequency of outdoor time may vary depending on individual factors such as age, ethnicity, and geographic location.  It is recommended to consult with an optometrist or ophthalmologist who specializes in pediatric eye care to determine the most appropriate treatment plan for your child based on their specific situation. |
| ChatGPT-4.0’s Response: | There is no specific amount of outdoor time that can guarantee the prevention of myopia or myopia progression in every child, as individual factors can vary. However, research suggests that spending more time outdoors is associated with a lower risk of developing myopia and may help slow down its progression.  A general recommendation, based on multiple studies, is to encourage children to spend at least 90 minutes to 2 hours per day outdoors. This can help reduce the risk of myopia onset and may slow down its progression. The protective effect of outdoor time is thought to be related to increased exposure to natural sunlight, which can stimulate the release of dopamine in the retina, reducing eye growth and the progression of myopia.  It is essential to remember that while increasing outdoor time can be beneficial, it may not entirely prevent myopia or its progression on its own. A comprehensive approach that considers genetic factors, lifestyle choices, and appropriate myopia management strategies under the guidance of an eye care professional is necessary for optimal outcomes. |
| Google Bard’s Response: | Studies have shown that spending at least 13 hours per week (just under 2 hours per day) outdoors can help to prevent myopia in children. This is thought to be due to the fact that bright sunlight helps to stimulate the production of dopamine, a neurotransmitter that is involved in eye growth.  For children who are already myopic, spending more time outdoors can help to slow the progression of the condition. A study published in the journal Ophthalmology found that children who spent an average of 76 minutes per day outdoors had a 50% lower risk of developing myopia than those who spent less time outdoors.  It is important to note that spending time outdoors is not a guarantee that your child will not develop myopia. However, it is a safe and effective way to reduce the risk of the condition.  Here are some tips for encouraging your child to spend more time outdoors:   1. Make outdoor activities a part of your family's routine. Go for walks, bike rides, or play catch in the yard. 2. Send your child to outdoor playdates or camps. 3. Encourage your child to spend time outdoors during the summer months. 4. Make sure your child wears sunglasses and sunscreen when they are outdoors.   By following these tips, you can help to keep your child's eyes healthy and prevent myopia. |

# Supplementary Table 7: Demonstration of ChatGPT4's self-correction capabilities using the Beta version of the Browse with Bing Plugin

| *Topic* | *Question* | *Summed Score* | | | | *Consensus-based rating* | | | |
| --- | --- | --- | --- | --- | --- | --- | --- | --- | --- |
|  |  | ***Initial*** | ***Self-Corrected*** | ***Bing Search 1^†^*** | ***Bing Search 2****^‡^* | ***Initial*** | ***Self-Corrected*** | ***Bing Search 1****^†^* | ***Bing Search 2****^‡^* |
| *Treatment and Prevention* | 1. What are the spectacles/contact lenses available to prevent myopia/ myopia progression? | 6 | 5 | 6 | 6 | Poor* | Borderline | Borderline | Borderline |
|  | 1. My child has not developed myopia, should he/ she start using atropine? | 6 | 6 | 5 | 6 | Poor* | Poor* | Borderline | Borderline |

*Where consensus on final accuracy rating was not reached (i.e., each grader provided a different rating), the lowest score (*‘poor’)* was assigned.
*^†^*Refers to responses generated by ChatGPT-4.0 during web searches.
*^‡^*Refers to evidence-based responses generated by ChatGPT-4.0 from web searches.

# Supplementary Table 7: ChatGPT-4.0’s, incorporated with Bing Search, Responses to Myopia-Related Questions

| **Topic: Treatment and Prevention** | |
| --- | --- |
| **Question:** | **4. What are the spectacles/contact lenses available to prevent myopia/ myopia progression?** |
| Response by ChatGPT4.0 incorporated with Bing Search *1^†^*: | 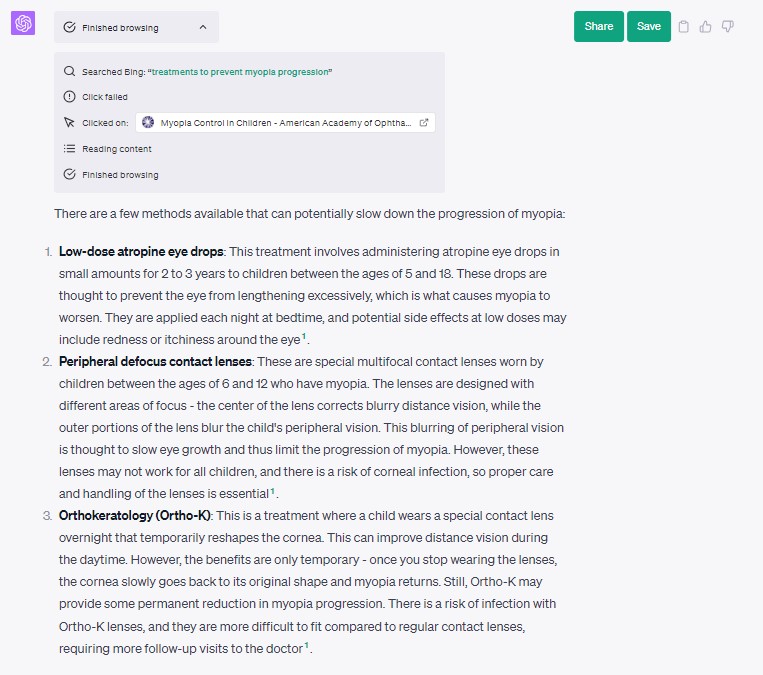 ^1^ <https://www.aao.org/eye-health/diseases/myopia-control-in-children> (Porter, Myopia control in children, 2022) |
| Response by ChatGPT4.0 incorporated with Bing Search 2*^‡^*: | 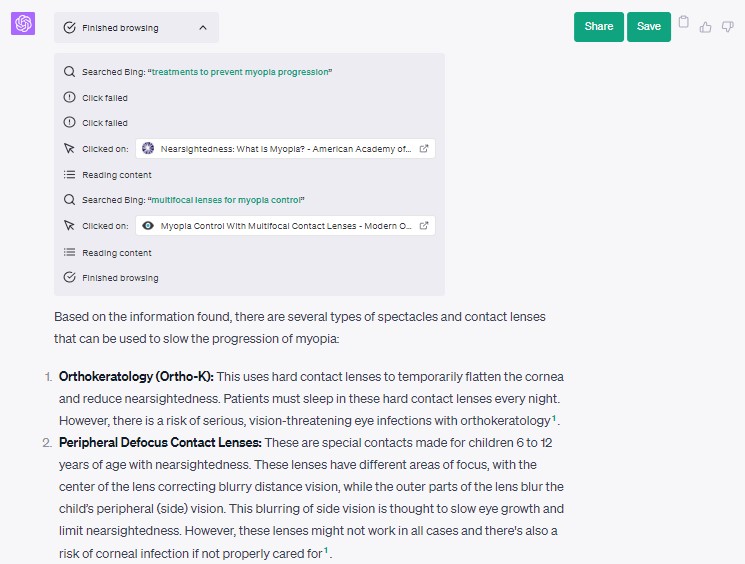  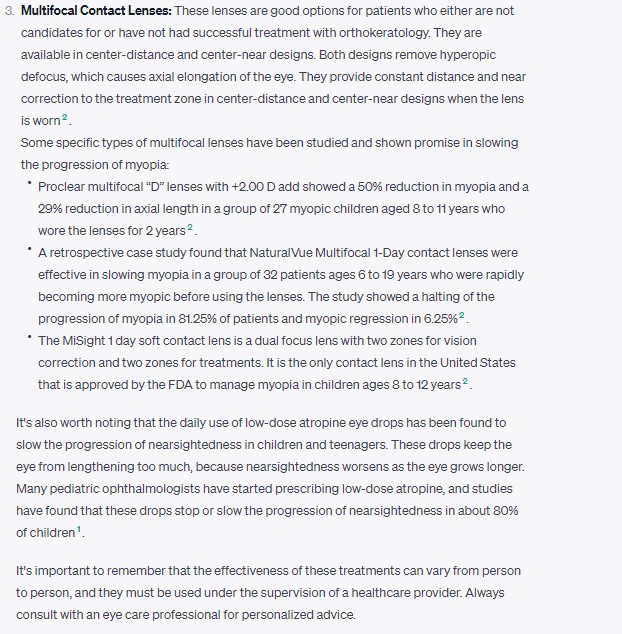 ^1^ <https://www.aao.org/eye-health/diseases/myopia-nearsightedness> (Turbert, Nearsightedness: What is myopia?, 2022)  ^2^ <https://modernod.com/articles/2020-may-june/myopia-control-with-multifocal-contact-lenses?c4src=article:infinite-scroll> (Achong-Coan, Myopia control with multifocal contact lenses, 2020) |
| **Question:** | **19. My child has not developed myopia, should he/ she start using atropine?** |
| Response by ChatGPT4.0 incorporated with Bing Search *1^†^*: | 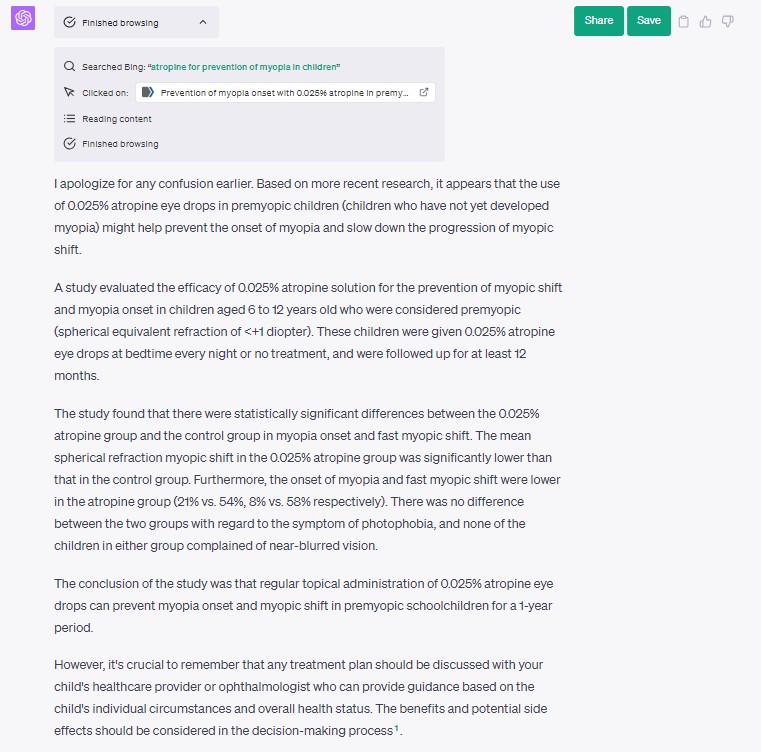  ^1^ <https://pubmed.ncbi.nlm.nih.gov/20698798/> (Fang et al., Prevention of myopia onset with 0.025% atropine in premyopic children, 2010) |
| Response by ChatGPT4.0 incorporated with Bing Search 2*^‡^*: | ^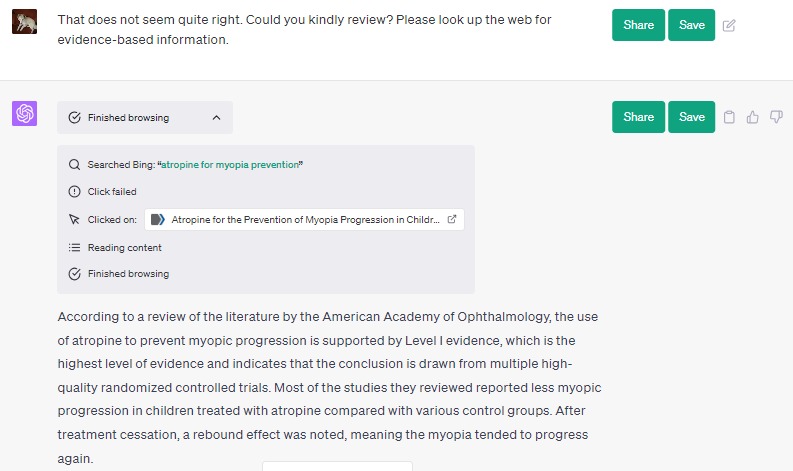^  ^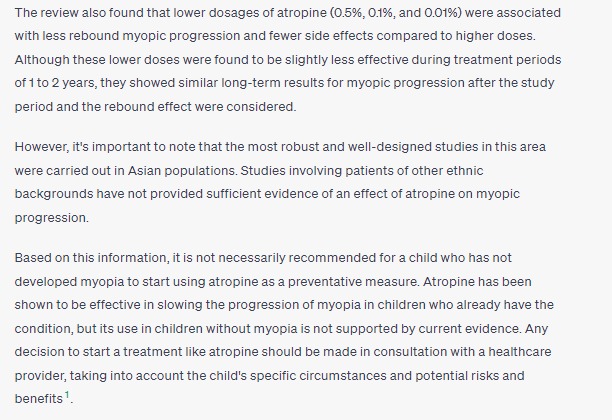^  ^1^ <https://pubmed.ncbi.nlm.nih.gov/28669492/> (Pineles et al., Atropine for the prevention of myopia progression in children, 2017) |

*^†^*Refers to responses generated by ChatGPT-4.0 during web searches.
*^‡^*Refers to evidence-based responses generated by ChatGPT-4.0 from web searches.
